# Vessel morphology depicted by three‐dimensional power Doppler ultrasound as second‐stage test in adnexal tumors that are difficult to classify: prospective diagnostic accuracy study

**DOI:** 10.1002/uog.22191

**Published:** 2021-02-01

**Authors:** P. Sladkevicius, L. Jokubkiene, D. Timmerman, D. Fischerova, C. Van Holsbeke, D. Franchi, L. Savelli, E. Epstein, R. Fruscio, J. Kaijser, A. Czekierdowski, S. Guerriero, M. A. Pascual, A. C. Testa, L. Ameye, L. Valentin

**Affiliations:** ^1^ Department of Obstetrics and Gynecology Skåne University Hospital Malmö Sweden; ^2^ Department of Clinical Sciences Malmö Lund University Malmö Sweden; ^3^ Department of Development and Regeneration, KU Leuven Leuven Belgium; ^4^ Department of Obstetrics and Gynecology and Leuven Cancer Institute University Hospitals Leuven Leuven Belgium; ^5^ Department of Obstetrics and Gynecology, First Faculty of Medicine Charles University and First Faculty of Medicine Prague Czech Republic; ^6^ Department of Obstetrics and Gynecology Ziekenhuis Oost Limburg Genk Belgium; ^7^ Preventive Gynecology Unit, Division of Gynecology European Institute of Oncology Milan Italy; ^8^ Gynecology and Reproductive Medicine Unit, S. Orsola‐Malpighi Hospital University of Bologna Bologna Italy; ^9^ Department of Clinical Science and Education Karolinska Institute, Södersjukhuset Stockholm Sweden; ^10^ Department of Obstetrics and Gynecology, San Gerardo Hospital University of Milan‐Bicocca Monza Italy; ^11^ Department of Obstetrics and Gynecology Ikazia Hospital Rotterdam Rotterdam The Netherlands; ^12^ 1st Department of Gynecological Oncology and Gynecology Medical University of Lublin Lublin Poland; ^13^ Department of Obstetrics and Gynecology University of Cagliari, Policlinico Universitario Duilio Casula, Monserrato Cagliari Italy; ^14^ Department of Obstetrics, Gynecology and Reproduction Hospital Universitari Dexeus Barcelona Spain; ^15^ Department of Gynecological Oncology Catholic University of the Sacred Heart Rome Italy; ^16^ Jules Bordet Institute Université Libre de Bruxelles Brussels Belgium

**Keywords:** Doppler, ovarian neoplasm, three‐dimensional ultrasound, ultrasonography, vascular morphology

## Abstract

**Objectives:**

To assess whether vessel morphology depicted by three‐dimensional (3D) power Doppler ultrasound improves discrimination between benignity and malignancy if used as a second‐stage test in adnexal masses that are difficult to classify.

**Methods:**

This was a prospective observational international multicenter diagnostic accuracy study. Consecutive patients with an adnexal mass underwent standardized transvaginal two‐dimensional (2D) grayscale and color or power Doppler and 3D power Doppler ultrasound examination by an experienced examiner, and those with a ‘difficult’ tumor were included in the current analysis. A difficult tumor was defined as one in which the International Ovarian Tumor Analysis (IOTA) logistic regression model‐1 (LR‐1) yielded an ambiguous result (risk of malignancy, 8.3% to 25.5%), or as one in which the ultrasound examiner was uncertain regarding classification as benign or malignant when using subjective assessment. Even when the ultrasound examiner was uncertain, he/she was obliged to classify the tumor as most probably benign or most probably malignant. For each difficult tumor, one researcher created a 360° rotating 3D power Doppler image of the vessel tree in the whole tumor and another of the vessel tree in a 5‐cm^3^ spherical volume selected from the most vascularized part of the tumor. Two other researchers, blinded to the patient's history, 2D ultrasound findings and histological diagnosis, independently described the vessel tree using predetermined vessel features. Their agreed classification was used. The reference standard was the histological diagnosis of the mass. The sensitivity of each test for discriminating between benign and malignant difficult tumors was plotted against 1 – specificity on a receiver‐operating‐characteristics diagram, and the test with the point furthest from the reference line was considered to have the best diagnostic ability.

**Results:**

Of 2403 women with an adnexal mass, 376 (16%) had a difficult mass. Ultrasound volumes were available for 138 of these cases. In 79/138 masses, the ultrasound examiner was uncertain about the diagnosis based on subjective assessment, in 87/138, IOTA LR‐1 yielded an ambiguous result and, in 28/138, both methods gave an uncertain result. Of the masses, 38/138 (28%) were malignant. Among tumors that were difficult to classify as benign or malignant by subjective assessment, the vessel feature ‘densely packed vessels’ had the best discriminative ability (sensitivity 67% (18/27), specificity 83% (43/52)) and was slightly superior to subjective assessment (sensitivity 74% (20/27), specificity 60% (31/52)). In tumors in which IOTA LR‐1 yielded an ambiguous result, subjective assessment (sensitivity 82% (14/17), specificity 79% (55/70)) was superior to the best vascular feature, i.e. changes in the diameter of vessels in the whole tumor volume (sensitivity 71% (12/17), specificity 69% (48/70)).

**Conclusion:**

Vessel morphology depicted by 3D power Doppler ultrasound may slightly improve discrimination between benign and malignant adnexal tumors that are difficult to classify by subjective ultrasound assessment. For tumors in which the IOTA LR‐1 model yields an ambiguous result, subjective assessment is superior to vessel morphology as a second‐stage test. © 2020 The Authors. Ultrasound in Obstetrics & Gynecology published by John Wiley & Sons Ltd on behalf of International Society of Ultrasound in Obstetrics and Gynecology.

**CONTRIBUTION**

**What are the novel findings of this work?**

Vessel morphology, depicted by three‐dimensional power Doppler ultrasound, differs between benign and malignant adnexal masses that are difficult to classify as such by an experienced ultrasound examiner using subjective assessment of ultrasound images or by the International Ovarian Tumor Analysis (IOTA) logistic regression model‐1 (LR‐1).

**What are the clinical implications of this work?**

Vessel morphology, as depicted by three‐dimensional power Doppler ultrasound, may slightly improve discrimination between benign and malignant adnexal tumors judged to be difficult to classify by subjective assessment. For tumors in which the IOTA LR‐1 model yields an ambiguous result, subjective assessment is superior to vessel morphology as a second‐stage test.

## INTRODUCTION

Subjective assessment of ultrasound findings (also called pattern recognition) by an experienced ultrasound examiner is the best ultrasound method for discriminating between benign and malignant adnexal masses^1^, ^2^. However, even an experienced ultrasound examiner may find up to 10% of tumors impossible to classify confidently as benign or malignant using pattern recognition (termed ‘difficult tumors’)^3^, ^4^, ^5^. The 10% risk cut‐off of the International Ovarian Tumor Analysis (IOTA) logistic regression model‐1 (LR‐1) has almost as good an ability to discriminate between benign and malignant tumors as has subjective assessment^6^, ^7^, but it has been suggested that a risk of malignancy calculated by LR‐1 of 8.3% to 25.5% represents an ambiguous risk^8^. The tumor marker CA 125 is clearly inferior to subjective assessment for discriminating between benign and malignant adnexal masses^9^ and has no role in classifying difficult tumors^3^, ^4^, ^5^, ^10^. Subjective assessment of ultrasound images is superior to computed tomography for discriminating between benign and malignant adnexal masses, while the role of magnetic resonance imaging is still unclear^11^, ^12^, ^13^. A logistic‐regression model for calculating the risk of malignancy in tumors not classifiable as benign or malignant by an experienced ultrasound examiner using subjective assessment has been published, but its ability to discriminate between benign and malignant tumors was not superior to that of subjective assessment^4^.

For tumors that are difficult to classify as benign or malignant using subjective assessment or using the IOTA LR‐1 model, a second‐stage test capable of correctly classifying difficult tumors as benign or malignant would be valuable. A possible second‐stage test is three‐dimensional (3D) power Doppler ultrasound examination of the vascular tree of tumors^14^.

The aims of this study were to assess whether vessel morphology, as depicted by 3D power Doppler ultrasound, differs between benign and malignant difficult adnexal masses, and whether vessel morphology improves discrimination between benign and malignant masses when used as a second‐stage test in difficult adnexal masses.

## PATIENTS AND METHODS

### Study population

Our study population comprised those patients in the IOTA‐3 study who had a difficult adnexal tumor (defined below)^15^. The IOTA‐3 study is a prospective observational international multicenter cross‐sectional diagnostic accuracy study (study protocol available in Appendix S1), which has been described in detail elsewhere^15^. Patients were recruited into IOTA‐3 between October 2009 and May 2012 in 18 centers in six countries (Sweden, Belgium, Italy, Poland, Spain and the Czech Republic). These centers were either oncology referral centers (i.e. tertiary referral centers with a specific gynecological oncology unit) or other hospitals or units with a special interest in gynecological ultrasound. The centers and type of center are listed after the main text. Ethical approval was obtained from the Ethics Committee of the University Hospitals Leuven (B32220095331/S51375) as well as from the local ethics committees of all participating centers.

Patients referred to one of the participating centers for an ultrasound examination and found to have an adnexal mass were eligible for inclusion in IOTA‐3. Consecutive patients with at least one adnexal mass judged not to be a functional cyst, examined with transvaginal ultrasound by an experienced ultrasound examiner, were included in IOTA‐3, provided that they gave written and/or oral informed consent before the ultrasound scan. If more than one mass was detected, the mass with the most complex ultrasound morphology was used for statistical analysis. When masses with similar morphology were observed, the largest mass or the one most easily accessible with ultrasound was used. Criteria for excluding patients from IOTA‐3 were pregnancy at the time of the ultrasound examination, surgical removal of the mass more than 120 days after the ultrasound examination, data inconsistencies that persisted after final manual data checks and cases with incomplete histology.

### Data collection

A dedicated, secure electronic data‐collection system was developed for the IOTA‐3 study (IOTA‐3 Study Screen; astraia Software, Munich, Germany). Patients automatically received a unique identifier. Data security was ensured by encrypting all data communications. Data integrity and completeness were ensured by client‐side checks in the system supplied by astraia and by final data cleaning by a group of biostatisticians and expert ultrasound examiners. The astraia software automatically calculated the risk of malignancy using the IOTA LR‐1 model^6^, and the result was displayed on the computer screen.

### Ultrasound examination

All patients included in the IOTA‐3 study underwent a standardized transvaginal ultrasound examination by a gynecologist or radiologist who was highly experienced in gynecologic ultrasound^15^, using high‐end ultrasound systems. Grayscale and color or power Doppler ultrasound was used to obtain information on more than 40 ultrasound variables to characterize each adnexal mass. The standardized ultrasound examination technique and the IOTA terminology used to describe the ultrasound images have been described elsewhere^16^. After completing the ultrasound examination, the examiner classified each mass as benign or malignant on the basis of his/her subjective assessment of the grayscale and color or power Doppler ultrasound findings. In addition, the examiner stated his/her level of confidence by classifying each mass as certainly benign, probably benign, uncertain, probably malignant or certainly malignant. This means that, even when the examiner was uncertain whether the tumor was benign or malignant, he/she was obliged to classify it as most probably benign or most probably malignant. The ultrasound information was entered prospectively into the electronic data‐collection system (see above), and was locked at the time of the examination and could not be changed thereafter. The decision regarding surgery for adnexal tumors was made by the referring physician, based on clinical information, such as symptoms, age, operative risk and coexisting disease, and on the ultrasound report, which was written using the results of subjective assessment.

In addition to performing the two‐dimensional (2D) ultrasound examination as described above, ultrasound examiners in centers with access to a Voluson 730 Expert or GE E8 ultrasound system (GE Healthcare, Zipf, Austria) with a 5–9‐MHz or a 6–12‐MHz vaginal transducer were asked to acquire 3D power Doppler ultrasound volumes of all adnexal masses. The power Doppler and 3D settings are described in Appendix S2. The examiners were instructed to include the whole tumor in the volume or, if this was not possible because the tumor was too large, to acquire several volumes to ensure that all parts of the tumor were captured. Patients were asked to lie still during the acquisition and, if necessary, to hold their breath. Volumes from difficult tumors, i.e. tumors in which the ultrasound examiner was uncertain whether the tumor was benign or malignant based on subjective assessment, or those in which the IOTA LR‐1 model gave an ambiguous risk of malignancy (8.3% to 25.5%)^8^, were sent to Skåne University Hospital, Malmö, on compact discs, for analysis. The same methodology of analyzing the 3D volumes as described previously was used^14^, and is outlined briefly below.

### Analysis of 3D volumes and audio‐video interleave (AVI) files

For each tumor, 360° rotating 3D power Doppler images (AVI‐files) of the vessel tree in the whole tumor as well as in a 5‐cm^3^ spherical volume selected from the most vascularized part of the tumor were prepared by the second author (L.J.) using the Virtual Organ Computer‐aided AnaLysis imaging program (4D‐View, version 7.0, GE Healthcare) on a personal computer. Subjective assessment was used to select the most vascularized part of the tumor^14^. Before creating the rotating image of the vessel tree, color transparency was adjusted to optimize the delineation of the vessels.

**Figure 1:**
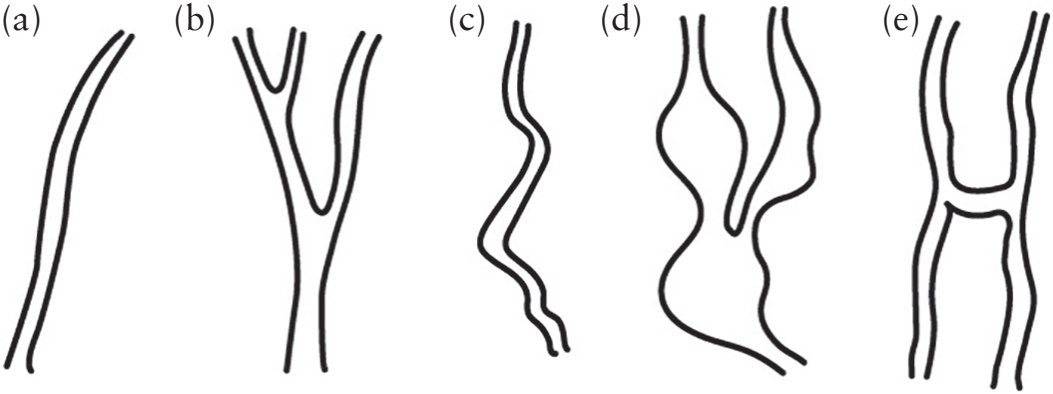
Schematic diagrams illustrating vascular features evaluated in adnexal masses in our study: (a) straight vessel with no branching; (b) branching vessel; (c) tortuous vessel; (d) vessel with changes in diameter; and (e) bridges (short, straight connections between two nearby vessels). Reproduced from Sladkevicius *et al*.^14^.

**Figure 2:**
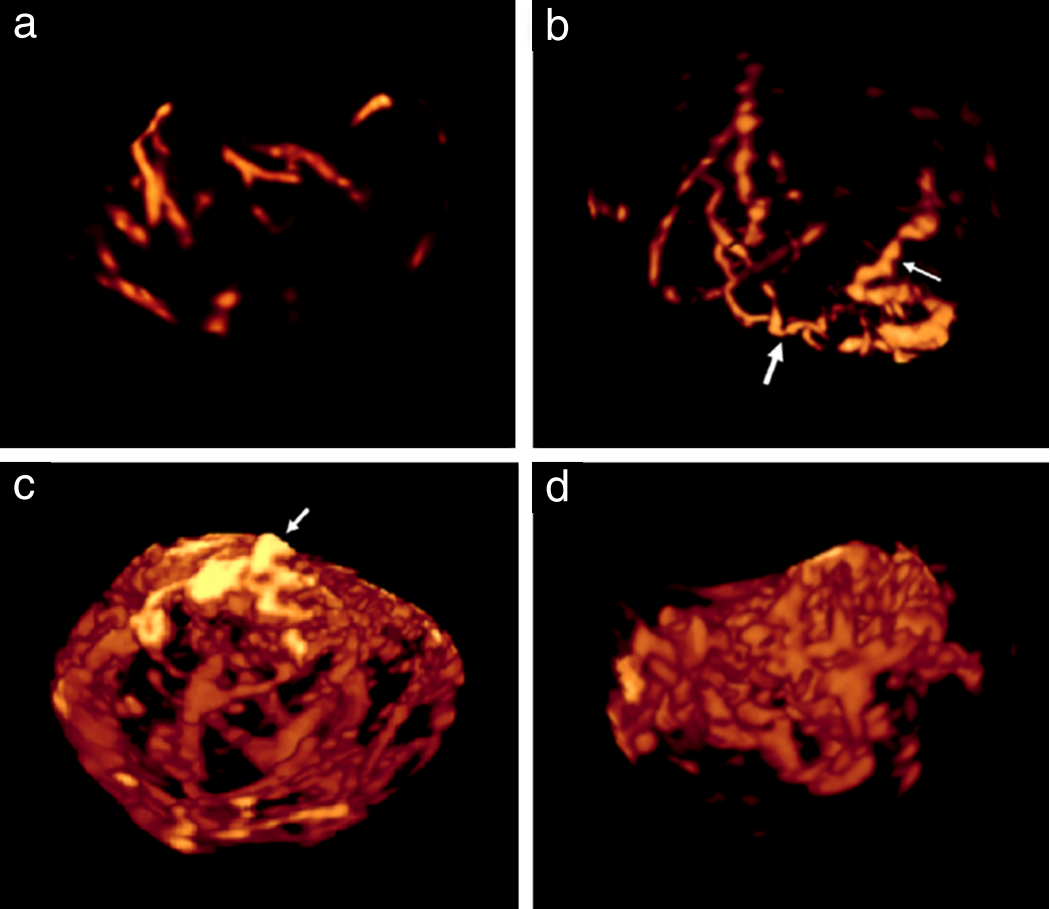
Three‐dimensional power Doppler ultrasound images of vessel tree in whole volume of ovarian tumors, showing: (a) dispersed (as opposed to densely packed), straight (as opposed to tortuous), branching vessels in benign mucinous cystadenoma; (b) dispersed, branching, tortuous vessels (thick arrow) with changes in diameter (thin arrow) in mucinous borderline tumor; (c) densely packed, branching, tortuous vessels with changes in diameter and color splashes (arrow shows splashes) in functional cyst; and (d) densely packed, branching, tortuous vessels with changes in diameter in ovarian clear‐cell cancer. Corresponding rotating images of vessel trees are shown in Videoclips S1–S4.

**Figure 3:**
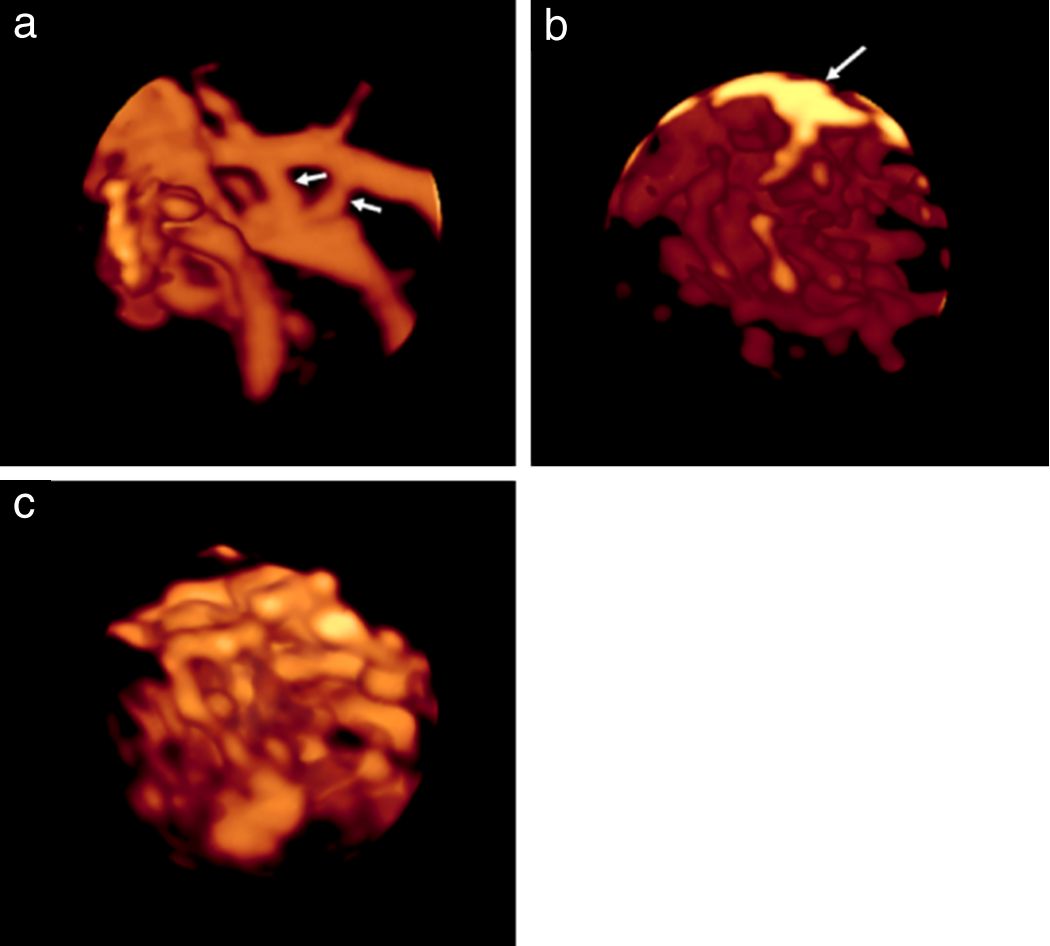
Three‐dimensional power Doppler ultrasound images of vessel tree in 5‐cm^3^ samples of ovarian tumors, showing: (a) bridges (arrows), i.e. straight connections between two nearby vessels, in struma ovarii; (b) branching, tortuous vessels with changes in diameter and color splashes (arrow) in benign ovarian fibroma; (c) branching, tortuous vessels with changes in diameter in ovarian endometrioid carcinoma (color splashes are seen in rotating image of same tumor in Videoclip S7). Corresponding rotating images of vessel trees are shown in Videoclips S5–S7.

All AVI files were then analyzed independently by two members of the Malmö research team (L.V., P.S.) who had no knowledge of patient history, 2D ultrasound findings or histological diagnosis. The vessel tree in the whole tumor volume, as well as in the 5‐cm^3^ sample, was characterized using the same classification as described previously (Figure 1): branching, i.e. division of a vessel into two or more branches; changes in diameter, i.e. changes in vessel width from narrow to wide and from wide to narrow; ‘splashes’, i.e. areas of color in contrast to clearly separate vessels; tortuosity; areas with densely packed vessels; and ‘bridges’, i.e. straight connections between two nearby vessels. The presence of bridges was assessed only in the 5‐cm^3^ samples and the presence of densely packed vessels was assessed only in the whole tumor volume^14^. In cases of disagreement between the two observers, consensus was reached by discussion, and the agreed classification was used for statistical analysis; this was done to decrease the risk of bias introduced by relying on one single observer. Ultrasound images of the vascular features are shown in Figures 2 and 3, and Videoclips S1–S7.

### Reference standard

The reference standard was the histological classification of the excised mass as malignant or benign. Histological examination was carried out at the local centers. Central pathology review was not performed because, in a previous IOTA study, no clinically significant differences between local and central pathology reports were observed^6^. Malignant tumors were classified according to the criteria recommended by the International Federation of Gynecology and Obstetrics^17^. Borderline ovarian tumors were classified as malignant. The pathologists were blinded to the ultrasound findings.

### Statistical analysis

SAS version 9.4 (SAS Institute, Cary, NC, USA) was used for statistical analysis. The statistical significance of differences in proportions was determined using the χ‐square test or Fisher's exact test and that of differences in continuous data using Student's *t*‐test or the Mann–Whitney *U*‐test, as appropriate.

We evaluated the discriminative ability of each vascular morphology feature, of subjective assessment and of the IOTA LR‐1 model when using the recommended 10% risk cut‐off for malignancy^6^, and expressed it as sensitivity, specificity and positive and negative likelihood ratios. The sensitivity of each test was plotted against 1 – specificity on a receiver‐operating‐characteristics (ROC) diagram; the test with the point furthest from the reference line was considered to have the best discriminative ability.

We estimated interobserver reliability (agreement beyond chance) in the assessment of the vascular tree of the tumors by calculating Cohen's κ^18^. κ values of 0.81–1.0 were taken to indicate excellent reliability, 0.61–0.80 good reliability and 0.41–0.60 moderate reliability^19^. *P* < 0.05 was regarded as statistically significant and was corrected for multiple testing using the permutation method^20^.

### Sample‐size calculation

We aimed to collect information on 300 difficult masses, of which we expected a minimum of 30% (*n* = 90) to be malignant^3^, ^8^. Ninety malignancies would give us reasonable 95% CIs around the point estimates for sensitivity. Because about 7% of all adnexal masses are difficult to classify as benign or malignant using subjective assessment^4^, and because the IOTA LR‐1 model may yield an ambiguous test result in 10% of all adnexal masses^8^, we estimated that we needed to examine about 2000 women with an adnexal mass (Appendix S1).

The study is reported using the Standards for Reporting Diagnostic Accuracy studies (STARD) guidelines^21^.

## RESULTS

In total, 2541 women with an adnexal mass were enrolled for inclusion in IOTA‐3, of whom 138 were excluded. Reasons for exclusion were > 120 days between ultrasound examination and surgery (*n* = 66), pregnancy (*n* = 31), data errors that could not be solved by contacting the principal investigators (*n* = 28) and incomplete final histology (*n* = 13). The final IOTA‐3 dataset included 2403 patients^15^, of whom 376 (16%) had a difficult tumor; an uncertain result regarding malignancy was obtained for 168 (7%) tumors on subjective assessment, for 259 (11%) tumors using the LR‐1 model and for 51 (2%) tumors using both methods. Serous and mucinous cystadenomas/cystadenofibromas, fibromas and borderline tumors were substantially more common among the difficult tumors than among the other ones, while endometriomas, benign teratomas (dermoid cysts), primary ovarian cancers and metastases in the ovaries from another primary tumor were substantially less common, with 2‐ to 3‐fold differences in prevalence (Tables S1 and S2). The distribution of histological diagnoses was similar in tumors in which the ultrasound examiner was uncertain about the diagnosis on subjective assessment and in those in which LR‐1 yielded an ambiguous result, with the exception that benign teratomas and endometriomas were more common in the latter (Table S1). Unilocular solid tumors, multilocular solid tumors and papillary projections were substantially more common in difficult tumors than in the others, while unilocular cysts, ground‐glass echogenicity of cyst fluid, and color scores of 1 and 4 were substantially less common, with 2‐ to 3‐fold differences in prevalence (Tables S3 and S4). The ultrasound characteristics of tumors that the examiner found difficult to classify on subjective assessment were similar to those in which LR‐1 yielded an ambiguous result, with the exception that ascites was more common and unilocular cysts were less common in tumors that the examiner found difficult to classify on subjective assessment (Table S3).

**Figure 4:**
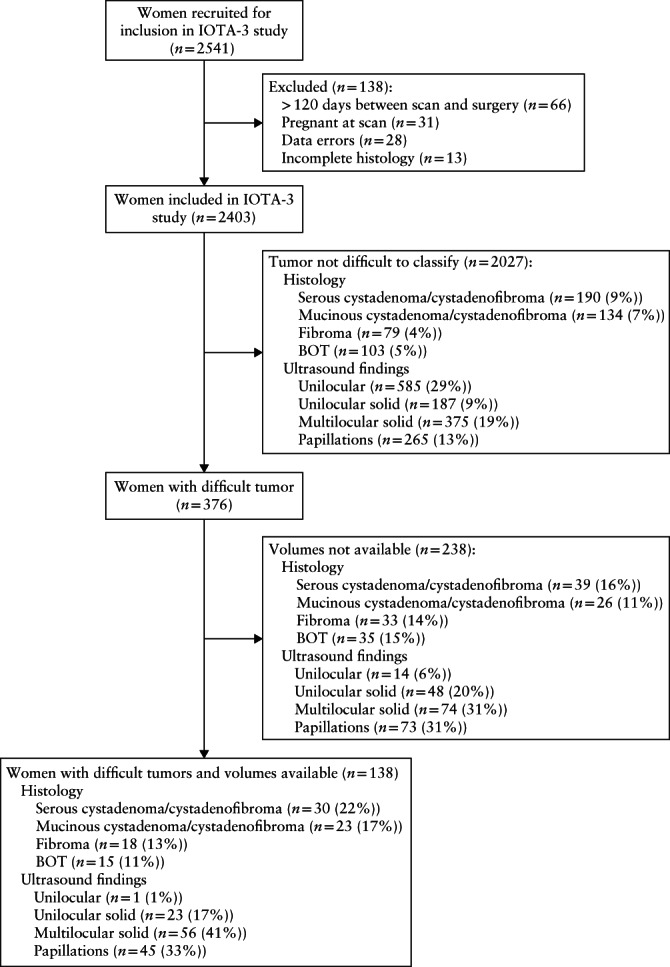
Flowchart summarizing recruitment of patients with adnexal tumor that was difficult to classify as benign or malignant. Prevalence of histological diagnoses and ultrasound features that differed most between tumors that were and those that were not difficult to classify are shown. BOT, borderline ovarian tumor.

3D ultrasound volumes were available for 138 of the 376 difficult tumors. Six centers did not provide volumes for any of their difficult masses (0/55), four centers provided volumes for more than 80% (73/87), four centers for between 40% and 57% (47/96), three centers for between 11% and 18% (16/107) and one center for 6% (2/31). In three centers, the reason for providing volumes for none or only a small proportion of the difficult tumors (6/78) was that the volumes stored in the ultrasound system had been deleted and there was no back‐up, and in one center the GE ultrasound system required was not always available for research. One center (with 33 difficult tumors) did not have access to a Voluson 730 Expert or GE E8 ultrasound system and so could not provide any volumes. Seven centers reported forgetfulness or transient technical problems to be the explanation for not providing volumes for all their difficult masses, and six centers gave no explanation. The number of patients and the proportion of difficult tumors contributed by each center are shown in Table S5, and patient flow is described in Figure 4.

The available volumes from the 138 difficult tumors included 79 (57%) masses in which subjective assessment gave an uncertain result, 87 (63%) cases in which LR‐1 gave an ambiguous result and 28 (20%) cases in which both methods gave an uncertain result. The histological diagnoses of the 138 difficult tumors with available volumes are shown in Table 1. One hundred (72%) tumors were benign and 38 (28%) were malignant. The distribution of histological diagnoses was similar to that in the 238 difficult tumors for which tumor volumes were not available, with the exception that the proportion of serous and mucinous cystadenomas/cystadenofibromas was slightly higher among the tumors with available volumes (Figure 4, Table S6). The clinical background data and 2D ultrasound findings for the 138 difficult tumors with available volumes are described in Table 2. They were similar to those in the 238 difficult tumors for which volumes were not available, with the following exceptions: unilocular cysts, incomplete septa, tender mass on scan and color score of 1 were less common in the 138 difficult tumors for which tumor volumes were available, while multilocular solid tumors and tumors with color score of 3 or 4 were more common (Figure 4, Table S7).

**Table 1:** Histological diagnoses of 138 adnexal tumors that were difficult to classify as benign or malignant and for which ultrasound volumes were available

Diagnosis	*n* (%)
Benign tumor	100 (72)
Endometrioma	7 (5)
Teratoma	5 (4)
Simple cyst or parasalpingeal cyst	3 (2)
Functional cyst	3 (2)
Hydrosalpinx or salpingitis	3 (2)
Peritoneal pseudocyst	2 (1)
Abscess	2 (1)
Fibroma	18 (13)
Serous cystadenoma/cystadenofibroma	30 (22)
Mucinous cystadenoma/cystadenofibroma	23 (17)
Rare benign tumor*	4 (3)
Borderline tumor	15 (11)
Stage I	14 (10)
Stage II	1 (1)
Stage III or IV	0 (0)
Primary invasive tumor	22 (16)
Stage I	7 (5)
Stage II	1 (1)
Stage III	8 (6)
Stage IV	0 (0)
Rare malignant tumor†	6 (4)
Metastasis in ovary from another primary tumor	1 (1)

* Rare benign tumors included one Brenner tumor and three cases of struma ovarii.

† Rare malignant tumors included three granulosa cell tumors, one immature teratoma, one Sertoli cell tumor and one gastrointestinal stromal tumor.

**Table 2:** Clinical background and ultrasound characteristics of 138 adnexal tumors that were difficult to classify as benign or malignant and for which ultrasound volumes were available

Characteristic	Value
Clinical variables	
Age (years)	54 ± 17
Postmenopausal	73 (53)
Hysterectomy	9 (7)
Hormonal replacement therapy	13 (9)
Personal history of ovarian cancer	4 (3)
Family history of ovarian cancer	3 (2)
CA 125 (U/mL)	20 (4–1302)*
Grayscale ultrasound variables	
Largest diameter (mm)	69 (10–310)
Bilateral tumors	22 (16)
Ascites	5 (4)
Type of mass	
Unilocular	1 (1)
Unilocular solid	23 (17)
Multilocular	32 (23)
Multilocular solid	56 (41)
Solid	26 (19)
Number of locules in cases of multilocular or multilocular solid tumor	
2	10/88 (11)
3	8/88 (9)
4	8/88 (9)
5–10	23/88 (26)
> 10	39/88 (44)
Tender mass on ultrasound examination	9 (7)
Echogenicity of cyst fluid	
Anechoic	36 (26)
Low level	47 (34)
Ground glass	10 (7)
Hemorrhagic	3 (2)
Mixed	16 (12)
No cyst fluid	26 (19)
Papillary projections	45 (33)
Flow in papillations	20/45 (44)
Number of papillations	2 (1–≥ 4)
Height of papillations (mm)	7 (3–45)
Mass with solid component	105 (76)
Largest diameter of largest solid component (mm)	24 (3–180)
Incomplete septum	2 (1)
Irregular walls	77 (56)
Shadows	20 (14)
Doppler ultrasound variables	
Color score	
1	10 (7)
2	44 (32)
3	70 (51)
4	14 (10)

Data are given as mean ± SD, *n* (%), *n*/*N* (%) or median (range).

* Data available for 117 cases.

**Figure 5:**
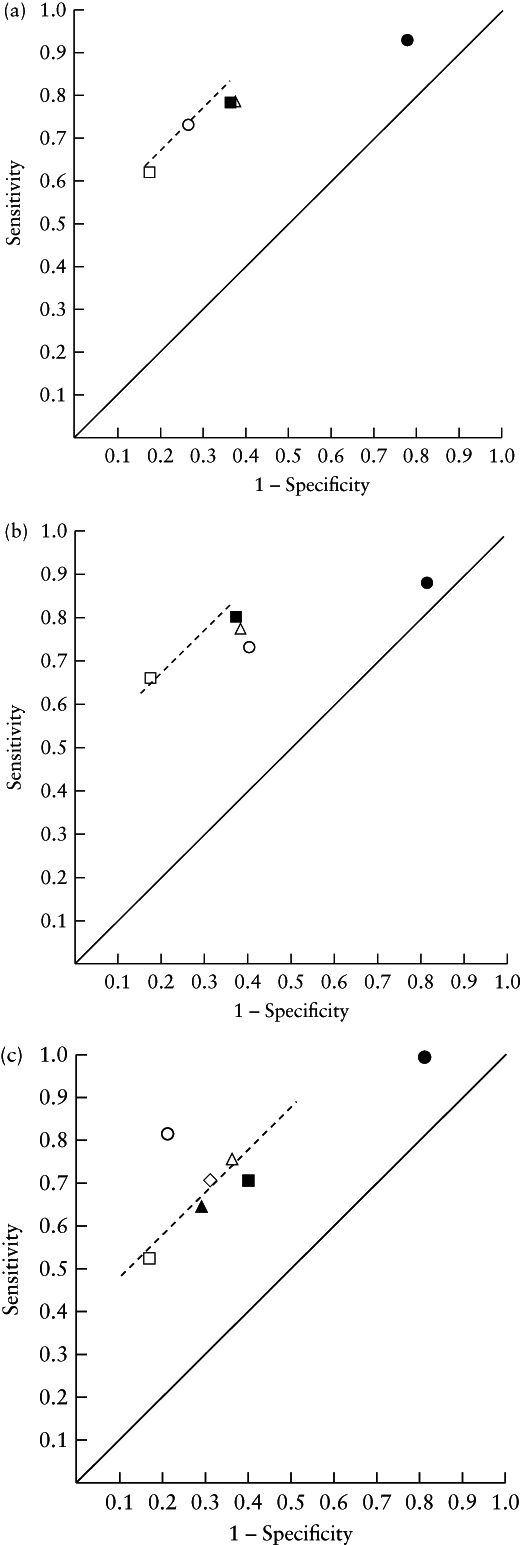
Ability of subjective ultrasound assessment (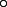), International Ovarian Tumor Analysis (IOTA) logistic regression model‐1 (LR‐1) (10% risk cut‐off) (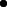) and best performing three‐dimensional power Doppler vessel morphology features, to discriminate between benign and malignant difficult adnexal tumors, in: (a) all 138 difficult tumors; (b) 79 difficult tumors in which ultrasound examiner was uncertain whether tumor was benign or malignant when using subjective assessment; and (c) 89 difficult tumors in which IOTA LR‐1 model yielded ambiguous result (risk of malignancy, 8.3% to 25.5%). Vessel morphology features: 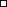, densely packed; 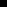, tortuous vessels in biopsy sample; 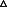, changes in diameter in biopsy sample; 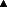, tortuous vessels in whole tumor volume; 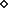, changes in diameter in whole tumor volume.

The ability of subjective assessment, the IOTA LR‐1 model and the vascular features to discriminate between benign and malignant difficult tumors, in terms of sensitivity and specificity, is shown in Table 3 (95% CIs are shown in Table S8). All vessel features differed significantly between benign and malignant difficult tumors. Branching vessels, densely packed vessels, changes in diameter, tortuous vessels, color splashes and bridges between vessels were more common in the malignant than in the benign difficult tumors. However, none of the vessel features discriminated well between benign and malignant difficult tumors. Figure 5 shows plots of sensitivity against 1 − specificity for subjective assessment, for the IOTA LR‐1 model when using a 10% risk cut‐off and for the vessel features with the best discriminative ability. Subjective assessment was the best method for discriminating between benign and malignant masses in the total study population of 138 difficult masses, followed by densely packed vessels in the whole tumor volume and tortuous vessels in the tumor biopsy sample. Among the 79 tumors that were difficult to classify as benign or malignant using subjective assessment, densely packed vessels in the whole tumor volume and tortuous vessels in the tumor biopsy sample had the best discriminative ability. For those tumors in which the IOTA LR‐1 model yielded an ambiguous result, subjective assessment had the best discriminative ability, while changes in diameter in the tumor biopsy sample had the second best.

Interobserver agreement and reliability with regard to vessel morphology are shown in Table 4. Interobserver reliability was moderate or good, with Cohen's κ values ranging from 0.55 to 0.77. Agreement was best for the assessment of densely packed vessels, branching vessels and tortuous vessels in the whole tumor volume (Cohen's κ, 0.77, 0.71 and 0.69, respectively) and for branching vessels in the 5‐cm^3^ tumor biopsy sample (Cohen's κ, 0.70).

**Table 3:** Ability of vessel morphology on three‐dimensional power Doppler ultrasound (US), subjective US assessment and International Ovarian Tumor Analysis logistic regression model‐1 (LR‐1) to discriminate correctly between benign and malignant difficult adnexal tumors

Diagnostic method	Sensitivity (% (*n*/*N*))	Specificity (% (*n*/*N*))	LR+	LR–	*P* *
US examiner uncertain or LR‐1 result ambiguous (*n* = 138)					
Whole tumor vessel morphology					
Branching vessels	89 (34/38)	33 (33/100)	1.34	0.32	0.009
Densely packed vessels	63 (24/38)	83 (83/100)	3.72	0.44	< 0.001
Diameter changes in vessels	66 (25/38)	68 (68/100)	2.06	0.50	< 0.001
Color splashes	50 (19/38)	78 (78/100)	2.27	0.64	0.002
Tortuous vessels	66 (25/38)	70 (70/100)	2.19	0.49	< 0.001
Biopsy vessel morphology					
Branching vessels	84 (32/38)	34 (34/100)	1.28	0.46	0.04
Diameter changes in vessels	79 (30/38)	63 (63/100)	2.13	0.33	< 0.001
Color splashes	53 (20/38)	69 (69/100)	1.70	0.69	0.02
Tortuous vessels	79 (30/38)	64 (64/100)	2.19	0.33	< 0.001
Bridges between vessels	42 (16/38)	81 (81/100)	2.22	0.72	0.007
Subjective assessment	74 (28/38)	74 (74/100)	2.83	0.36	< 0.001
LR‐1 (10% risk cut‐off)	92 (35/38)	23 (23/100)	1.20	0.34	0.03
US examiner uncertain (*n* = 79)					
Whole tumor vessel morphology					
Branching vessels	89 (24/27)	33 (17/52)	1.32	0.34	0.06
Densely packed vessels	67 (18/27)	83 (43/52)	3.85	0.40	< 0.001
Diameter changes in vessels	63 (17/27)	67 (35/52)	1.93	0.55	0.01
Color splashes	52 (14/27)	83 (43/52)	3.00	0.58	0.003
Tortuous vessels	63 (17/27)	63 (33/52)	1.72	0.58	0.02
Biopsy vessel morphology					
Branching vessels	85 (23/27)	35 (18/52)	1.30	0.43	0.07
Diameter changes in vessels	78 (21/27)	62 (32/52)	2.02	0.36	0.001
Color splashes	59 (16/27)	71 (37/52)	2.05	0.57	0.009
Tortuous vessels	81 (22/27)	67 (35/52)	2.49	0.28	< 0.001
Bridges between vessels	44 (12/27)	79 (41/52)	2.10	0.71	0.03
Subjective assessment	74 (20/27)	60 (31/52)	1.83	0.44	0.004
LR‐1 (10% risk cut‐off)	89 (24/27)	19 (10/52)	1.10	0.58	0.34
LR‐1 result ambiguous (*n* = 87)					
Whole tumor vessel morphology					
Branching vessels	94 (16/17)	30 (21/70)	1.35	0.20	0.06
Densely packed vessels	53 (9/17)	83 (58/70)	3.09	0.57	0.004
Diameter changes in vessels	71 (12/17)	69 (48/70)	2.25	0.43	0.005
Color splashes	47 (8/17)	77 (54/70)	2.06	0.69	0.07
Tortuous vessels	65 (11/17)	71 (50/70)	2.27	0.49	0.01
Biopsy vessel morphology					
Branching vessels	76 (13/17)	31 (22/70)	1.12	0.75	0.77
Diameter changes in vessels	76 (13/17)	64 (45/70)	2.14	0.37	0.005
Color splashes	47 (8/17)	69 (48/70)	1.50	0.77	0.26
Tortuous vessels	71 (12/17)	60 (42/70)	1.77	0.49	0.03
Bridges between vessels	35 (6/17)	81 (57/70)	1.90	0.80	0.19
Subjective assessment	82 (14/17)	79 (55/70)	3.84	0.23	< 0.001
LR‐1 (10% risk cut‐off)	100 (17/17)	19 (13/70)	1.23	†	0.06

* *P*‐value for difference in rate of vascular feature between benign and malignant tumors.

† Not possible to calculate. LR‐1 model uses 10% risk cut‐off to predict malignancy^6^, ^7^. Correction for multiple testing not done because this is an exploratory analysis. LR+, positive likelihood ratio; LR–, negative likelihood ratio.

**Table 4:** Interobserver agreement for assessment of vessel morphology by three‐dimensional power Doppler ultrasound in 138 difficult adnexal tumors

Variable	% agreement (95% CI)	Kappa (95% CI)
Whole tumor		
Branching	88 (82–93)	0.71 (0.58–0.84)
Densely packed	90 (84–94)	0.77 (0.65–0.88)
Diameter changes	78 (71–84)	0.55 (0.41–0.69)
Color splashes	81 (74–87)	0.57 (0.42–0.71)
Tortuous	85 (78–90)	0.69 (0.56–0.81)
Tumor biopsy		
Branching	88 (81–92)	0.70 (0.57–0.83)
Diameter changes	78 (70–84)	0.55 (0.41–0.69)
Color splashes	86 (79–90)	0.67 (0.54–0.80)
Tortuous	83 (75–88)	0.65 (0.52–0.78)
Bridges	86 (80–91)	0.63 (0.47–0.78)

## DISCUSSION

We have shown that vessel morphology, as seen on 3D power Doppler ultrasound, differs between benign and malignant difficult adnexal tumors. Branching vessels, changes in diameter, color splashes, tortuous vessels, densely packed vessels and bridges between vessels were more common in malignant than in benign difficult tumors. However, none of the vascular features discriminated well between benign and malignant difficult tumors. Our findings confirm that interobserver reliability with regard to vessel morphology depicted by 3D power Doppler is moderate to good^14^_._

To the best of our knowledge, there are no published studies exploring the ability of the morphology of tumor vessels depicted by 3D power Doppler ultrasound to discriminate between benign and malignant difficult tumors. A strength of our study is that vessel morphology was assessed by observers who were blinded to clinical information, 2D grayscale or color Doppler ultrasound findings and histological diagnosis of the tumors. This means that our results regarding evaluation of the vessel tree are unbiased and reflect the true discriminative capacity of vessel morphology. A limitation of our study is possible selection bias, because not all the participating centers provided 3D volumes of all their difficult tumors. The histology and ultrasound features differed slightly between the difficult tumors included (i.e. those with tumor volumes available) and those not included (i.e. those with tumor volumes not available) in that the proportion of serous and mucinous cystadenomas/cystadenofibromas, multilocular solid tumors and tumors with a color score of 3 or 4 was higher among the difficult tumors that were included, while the proportion of unilocular cysts was lower (Figure 4, Tables S6 and S7). However, the histological diagnoses and ultrasound features of the difficult tumors with available volumes were fairly similar to those of all difficult tumors included in IOTA‐3 (Table S1). Therefore, our study sample should be reasonably representative of all difficult adnexal tumors. The small number of difficult masses with available volumes is another limitation, making our estimates of sensitivity and specificity imprecise (Table S8). However, because this was an exploratory study, we believe that this is acceptable.

Our results confirm those of a previous study demonstrating that serous and mucinous cystadenomas/cystadenofibromas, fibromas and borderline tumors are difficult to classify as benign or malignant, and that unilocular solid and multilocular solid tumors and tumors with papillary projections are over‐represented among difficult tumors^4^. The proportion of tumors that the ultrasound examiner found difficult to classify based on subjective assessment in the current study is the same as that in the previously published study (168/2403 (7%) *vs* 244/3511 (7%))^4^.

To the best of our knowledge, the ability of vessel morphology depicted by 3D ultrasound to discriminate between benign and malignant adnexal masses or to decrease diagnostic errors has been explored in only one published study^14^, which included 104 adnexal masses reasonably representative of a general population of tumors scheduled for surgery. Similar to our findings, all vascular features differed between benign and malignant tumors, but the discriminated much better between benign and malignant tumors than in the current study^14^. This is not surprising because, in the cited study, the tumors were more heterogeneous with fewer overlapping ultrasound features. The authors found that adding a vascular‐feature variable to a logistic‐regression model including only grayscale ultrasound variables improved the discriminative ability of the model only minimally (increase in area under the ROC curve from 0.98 to 0.99) because the grayscale model itself performed extremely well. The authors concluded that, in an ordinary population of ovarian tumors, 3D power Doppler ultrasound examination adds little to grayscale imaging. However, they hypothesized that 3D power Doppler ultrasound with assessment of the morphology of tumor vessels might be useful in difficult tumors.

Our results show that, if the IOTA LR‐1 model gives an ambiguous risk estimate (8.3% to 25.5%), then subjective assessment by an experienced ultrasound examiner is superior to using vessel morphology depicted by 3D power Doppler ultrasound as a second‐stage test. However, if a mass cannot be confidently classified as benign or malignant by an experienced ultrasound examiner using subjective assessment, then assessing vessel morphology with 3D power Doppler ultrasound could be useful. Both densely packed vessels in the whole tumor volume and tortuous vessels in a 5‐cm^3^ tumor biopsy can be used for discrimination (Figure 5). We recommend using densely packed vessels because interobserver reliability was better for this variable than for tortuous vessels in a tumor biopsy. On the other hand, it is more time consuming to analyze a whole tumor volume than a tumor biopsy^22^. In our experience, for a highly experienced ultrasound examiner, it takes a minimum of 2.5 min to create a rotating 3D image of the vascular tree of a whole tumor, while it takes a minimum of 1 min to create one for a 5‐cm^3^ biopsy selected from the most vascularized part of the tumor. When assessing vessel morphology, it is important to be aware of the pitfalls of Doppler ultrasound and to ensure that Doppler settings are correct. If the tumor is far away from the ultrasound probe, it might not be possible to detect Doppler signals from the whole or parts of the tumor. This limits the clinical usefulness of vessel morphology for classifying tumors as benign or malignant. Another limiting factor is the subjectivity of the method. Evaluation of vessel morphology, including ‘densely packed vessels’, is based on pattern recognition and is therefore difficult to standardize or define.

Correct classification of adnexal masses as benign or malignant is a requirement for optimal management, i.e. conservative management with follow‐up examinations, surgery in a local hospital or referral to a center specialized in gynecological oncology^23^. However, some tumors are difficult to classify confidently as benign or malignant. Vessel morphology as depicted by 3D power Doppler ultrasound shows limited ability to discriminate between benign and malignant difficult tumors. It remains to be seen whether new biomarkers, immune cells, proteins or genetic information can improve the classification of difficult tumors as benign or malignant.

## Supporting information


**Appendix S1** International Ovarian Tumor Analysis (IOTA) Phase 3 study protocol

**Appendix S2** Power Doppler and three‐dimensional (3D) ultrasound settings used in study

**Videoclips S1–S7** Three‐dimensional 360° rotating power Doppler ultrasound images of vessel tree in: benign mucinous cystadenoma, showing dispersed (as opposed to densely packed), straight (as opposed to tortuous), branching vessels (Videoclip S1; still image of same tumor is Figure 2a); mucinous borderline tumor, showing dispersed, branching, tortuous vessels with diameter changes (Videoclip S2; still image of same tumor is Figure 2b); functional cyst, showing densely packed, branching, tortuous vessels with diameter changes and color splashes (Videoclip S3; still image of same tumor is Figure 2c); ovarian clear cell cancer, showing densely packed, branching, tortuous vessels with diameter changes (Videoclip S4; still image of same tumor is Figure 2d); 5‐cm^3^ sample of struma ovarii showing bridges, i.e. straight connections between two nearby vessels (Videoclip S5; still image of same tumor is Figure 3a); 5‐cm^3^ sample of ovarian fibroma, showing branching, tortuous vessels with diameter changes and color splashes (Videoclip S6; still image of same tumor is Figure 3b); and 5‐cm^3^ sample of ovarian endometrioid carcinoma, showing branching, tortuous vessels with diameter changes and color splashes (Videoclip S7; still image of same tumor is Figure 3c).

**Tables S1 and S2** Histological diagnoses of 2403 adnexal tumors, according to: whether tumor was difficult to classify and availability of ultrasound volumes (Table S1) and whether tumor was difficult to classify as benign or malignant (Table S2)

**Tables S3 and S4** Clinical and ultrasound characteristics of 2403 adnexal tumors, according to: whether tumor was difficult to classify and availability of ultrasound volumes (Table S3) and whether tumor was difficult to classify as benign or malignant (Table S4)

**Table S5** Number of patients and proportion of difficult adnexal tumors contributed by each center

**Tables S6 and S7** Histological diagnoses (Table S6) and clinical and ultrasound characteristics (Table S7) of 376 difficult adnexal tumors, according to whether ultrasound volumes were available

**Table S8** Ability (with 95% CI) of vessel morphology on 3D power Doppler, subjective ultrasound assessment and IOTA logistic regression model 1 to discriminate correctly between benign and malignant difficult adnexal tumors

## References

[1] Valentin L , Hagen B , Tingulstad S , Eik‐Nes S. Comparison of ‘pattern recognition’ and logistic regression models for discrimination between benign and malignant pelvic masses: a prospective cross validation. Ultrasound Obstet Gynecol 2001; 18: 357–365. 11778996 10.1046/j.0960-7692.2001.00500.x

[2] Timmerman D . The use of mathematical models to evaluate pelvic masses; can they beat an expert operator? Best Pract Res Clin Obstet Gynaecol 2004; 18: 91–104. 15123060 10.1016/j.bpobgyn.2003.09.009

[3] Valentin L , Ameye L , Jurkovic D , Metzger U , Lécuru F , Van Huffel S , Timmerman D. Which extrauterine pelvic masses are difficult to correctly classify as benign or malignant on the basis of ultrasound findings and is there a way of making a correct diagnosis? Ultrasound Obstet Gynecol 2006; 27: 438–444. 16526098 10.1002/uog.2707

[4] Valentin L , Ameye L , Savelli L , Fruscio R , Leone FP , Czekierdowski A , Lissoni AA , Fischerova D , Guerriero S , Van Holsbeke C , Van Huffel S , Timmerman D . Adnexal masses difficult to classify as benign or malignant using subjective assessment of gray‐scale and Doppler ultrasound findings: logistic regression models do not help. Ultrasound Obstet Gynecol 2011; 38: 456–465. 21520475 10.1002/uog.9030

[5] Valentin L , Ameye L , Savelli L , Fruscio R , Leone FP , Czekierdowski A , Lissoni AA , Fischerova D , Guerriero S , Van Holsbeke C , Van Huffel S , Timmerman D . Unilocular adnexal cysts with papillary projections but no other solid components: is there a diagnostic method that can classify them reliably as benign or malignant before surgery? Ultrasound Obstet Gynecol 2013; 41: 570–581. 22915541 10.1002/uog.12294

[6] Timmerman D , Testa AC , Bourne T , Ferrazzi E , Ameye L , Konstantinovic ML , Van Calster B , Collins WP , Vergote I , Van Huffel S , Valentin L ; International Ovarian Tumor Analysis Group . Logistic regression model to distinguish between the benign and malignant adnexal mass before surgery: a multicenter study by the International Ovarian Tumor Analysis Group. J Clin Oncol 2005; 23: 8794–8801. 16314639 10.1200/JCO.2005.01.7632

[7] Timmerman D , Van Calster B , Testa AC , Guerriero S , Fischerova D , Lissoni AA , Van Holsbeke C , Fruscio R , Czekierdowski A , Jurkovic D , Savelli L , Vergote I , Bourne T , Van Huffel S , Valentin L. Ovarian cancer prediction in adnexal masses using ultrasound‐based logistic regression models: a temporal and external validation study by the IOTA group. Ultrasound Obstet Gynecol 2010; 36: 226–234. 20455203 10.1002/uog.7636

[8] Daemen A , Valentin L , Fruscio R , Van Holsbeke C , Melis GB , Guerriero S , Czekierdowski A , Jurkovic D , Ombelet W , Rossi A , Vergote I , Bourne T , De Moor B , Timmerman D. Improving the preoperative classification of adnexal masses as benign or malignant by second‐stage tests. Ultrasound Obstet Gynecol 2011; 37: 100–106. 20814878 10.1002/uog.8813

[9] Van Calster B , Timmerman D , Bourne T , Testa AC , Van Holsbeke C , Domali E , Jurkovic D , Neven P , Van Huffel S , Valentin L. Discrimination between benign and malignant adnexal masses by specialist ultrasound examination versus serum CA125. J Natl Cancer Inst 2007; 99: 1706–1714. 18000221 10.1093/jnci/djm199

[10] Valentin L , Jurkovic D , Van Calster B , Testa A , Van Holsbeke C , Bourne T , Vergote I , Van Huffel S , Timmerman D. Adding a single CA125 measurement to ultrasound imaging performed by an experienced examiner does not improve preoperative discrimination between benign and malignant adnexal masses. Ultrasound Obstet Gynecol 2009; 34: 345–354. 19585547 10.1002/uog.6415

[11] Valentin L . Imaging in gynecology. Best Pract Res Clin Obstet Gynaecol 2006; 20: 881–906. 16904942 10.1016/j.bpobgyn.2006.06.001

[12] Kaijser J , Vandecaveye V , Deroose CM , Rockall A , Thomassin‐Naggara I , Bourne T , Timmerman D . Imaging techniques for the pre‐surgical diagnosis of adnexal tumours. Best Pract Res Clin Obstet Gynaecol 2014; 28: 683–695. 24780415 10.1016/j.bpobgyn.2014.03.013

[13] Pereira PN , Sarian LO , Yoshida A , Araújo KG , Silva ACB , de Oliveira Barros RH , Jales RM , Derchain S . Improving the performance of IOTA simple rules: sonographic assessment of adnexal masses with resource‐effective use of a magnetic resonance scoring (ADNEX MR scoring system). Abdom Radiol (NY) 2020; 45: 3218–3229. 31482379 10.1007/s00261-019-02207-9

[14] Sladkevicius P , Jokubkiene L , Valentin L. Contribution of morphological assessment of the vessel tree by three‐dimensional ultrasound to a correct diagnosis of malignancy in ovarian masses. Ultrasound Obstet Gynecol 2007; 30: 874–882. 17943717 10.1002/uog.5150

[15] Testa A , Kaijser J , Wynants L , Fischerova D , Van Holsbeke C , Franchi D , Savelli L , Epstein E , Czekierdowski A , Guerriero S , Fruscio R , Leone FP , Vergote I , Bourne T , Valentin L , Van Calster B , Timmerman D . Strategies to diagnose ovarian cancer: new evidence from phase 3 of the multicentre international IOTA study. Br J Cancer 2014; 111: 680–688. 24937676 10.1038/bjc.2014.333 PMC4134495

[16] Timmerman D , Valentin L , Bourne TH , Collins WP , Verrelst H , Vergote I ; International Ovarian Tumor Analysis (IOTA) Group . Terms, definitions and measurements to describe the sonographic features of adnexal tumors: a consensus opinion from the International Ovarian Tumor Analysis (IOTA) Group. Ultrasound Obstet Gynecol 2000; 16: 500–505. 11169340 10.1046/j.1469-0705.2000.00287.x

[17] Heintz APM , Odicino F , Maisonneuve P , Beller U , Benedet JL , Creasman WT , Ngan HYS , Pecorelli S . Carcinoma of the Ovary, 25th Annual Report on the Results of Treatment in Gynecological Cancer. Int J Gynecol Obstet 2003; 83 (Suppl 1): S135–S166.

[18] Cohen J. A coefficient of agreement for nominal scales. Educ Psychol Meas 1960; 20: 37–46.

[19] Brennan P , Silman A. Statistical methods for assessing observer variability in clinical measures. BMJ 1992; 304: 1491–1494. 1611375 10.1136/bmj.304.6840.1491 PMC1882212

[20] Westfall PH , Wolfinger RD . Multiple tests with discrete distributions. Am Stat 1997; 51: 3 *–* 8.

[21] Cohen JF , Korevaar DA , Altman DG , Bruns DE , Gatsonis CA , Hooft L , Irwig L , Levine D , Reitsma JB , de Vet HC , Bossuyt PM . STARD 2015 guidelines for reporting diagnostic accuracy studies: explanation and elaboration. BMJ Open 2016; 6: e012799. 10.1136/bmjopen-2016-012799 PMC5128957 28137831

[22] Jokubkiene L , Sladkevicius P , Valentin L. Does three‐dimensional power Doppler ultrasound help in discrimination between benign and malignant ovarian masses? Ultrasound Obstet Gynecol 2007; 29: 215–225. 17201017 10.1002/uog.3922

[23] Froyman W , Landolfo C , De Cock B , Sladkevicius P , Testa AC , Van Holsbeke C , Domali E , Fruscio R , Epstein E , Dos Santos Bernardo MJ , Franchi D , Kudla MJ , Chiappa V , Alcazar JL , Leone FPG , Buonomo F , Hochberg L , Coccia ME , Guerriero S , Deo N , Jokubkiene L , Kaijser J , Coosemans A , Vergote I , Verbakel JY , Bourne T , van Calster B , Valentin L , Timmerman D . Risk of complications in patients with conservatively managed ovarian tumours (IOTA5): a 2‐year interim analysis of a multicentre, prospective, cohort study. Lancet Oncol 2019; 20: 448–458. 30737137 10.1016/S1470-2045(18)30837-4

