# Supplementary material for: Vessel morphology depicted by three‐dimensional power Doppler ultrasound as second‐stage test in adnexal tumors that are difficult to classify: prospective diagnostic accuracy study
Source: Ultrasound Obstet Gynecol. 2021 Feb 1;57(2):324–34. doi: 10.1002/uog.22191 (PMC7898332; doi:10.1002/uog.22191)
Supplement: Supplementary file 1 — Appendix S1 International Ovarian Tumor Analysis (IOTA) Phase 3 study protocol [file UOG-57-324-s001.pdf]

International Ovarian Tumour Analysis (IOTA) Phase 3

A multicentre study on the pre-operative characterisation of ovarian tumours based on artificial intelligence models.

STUDY CO-ORDINATOR

Dirk Timmerman, MD, PhD
Department of Obstetrics and Gynaecology, University Hospitals Leuven,
Herestraat 49, B-3000 Leuven, BELGIUM.
Telephone: + 32 16 344201 (office) Fax: + 32 16 344205
+ 32 16 344215 (secretary) Mobile + 32 497 896025
E-mail: dirk.timmerman@uzleuven.be

STEERING COMMITTEE

Tom Bourne (Imperial College, University College London)
Dirk Timmerman (University Hospitals, Leuven)
Antonia C. Testa (Università Cattolica di Sacro Cuore, Roma)
Lil Valentin (University of Lund / Malmö)
Sabine Van Huffel (ESAT-SISTA, K.U. Leuven)
Ignace Vergote (University Hospitals, Leuven)

LIST OF PRINCIPAL INVESTIGATORS AND PARTICIPATING CENTRES (AND CENTRE CODE)

LPO (i.e. centre code)
Artur Czekierdowski, MD, PhD
Professor, 1st Department of Gynecology, Medical University in Lublin
16 Staszica Str., 20-081 Lublin, POLAND
Telephone: +4881 532 78 47 Fax: +4881 532 06 08
E-mail: ginonkol@am.lublin.pl
E-mail: a.czekierdowski@am.lublin.pl

LSW
Elisabeth Epstein, MD, PhD
Department of Obstetrics and Gynaecology, Lund University,
Lund, SWEDEN
Telephone: +46-46-171000
E-mail: Elisabeth.Epstein@med.lu.se

PCR
Daniela Fischerová, MD
Oncogynecological Center Department of Obstetrics and Gynecology
General Faculty Hospital of Charles University
Apolinarska 18, Prague 2, CZECH REPUBLIC
Telephone: +42 0603285097 (mobile)

E-mail: daniela.fischerova@seznam.cz

CIT
Dorella Franchi, MD
Dt.ssa Ginecologic Oncology Unit, Division of Gynecology,
IEO, Milano, ITALY
E-mail: dorella.franchi@ieo.it

GIT
Stefano Greggi, MD, PhD
Director Gynecologic Oncology
Instituto Nat del Tumori, Fondazione Pascale
Via Mariano Semmola, 1
80121 Napoli, ITALY
Telephone: +39 81 5903417 Fax: +39 81 5903851
E-mail: s.greggi@tin.it

SIT
Stefano Guerriero, MD, PhD
Department of Obstetrics and Gynaecology, University of Cagliari,
Ospedale San Giovanni di Dio
Via Ospedale 46, 09124, Cagliari, Sardinia, ITALY
Telephone: +39 070 6092467 Fax: +39 070 668575
E-mail: gineca.sguerriero@tiscali.it

BCH
Jingzhang, MD
Professor, Ultrasound Department, Chinase PLA General Hospital
No.28 Fuxing Road, Beijing, P.R. of CHINA 100853
Telephone: +86 010 88626007 Fax: +86 010 88626007
E-mail: zjbch@vip.sina.com
E-mail: zjbch@hotmail.com

KUK
Davor Jurkovic, MD, PhD, MRCOG
Consultant, Department of Obstetrics and Gynaecology,
University College Hospital, London, UK
e-mail davor.jurkovic@kcl.ac.uk

MIT
Francesco P.G. Leone, MD, PhD
DSC L. Sacco, Università di Milano, Milano, ITALY.
Telephone: +39 02 39042264 Fax: +39 02 3565061
E-mail: f.leone@hsacco.it

OIT
Robert Fruscio, MD
Andrea A. Lissoni, MD
Clinica Ostetrica e Ginecologica, Ospedale S. Gerardo, Università di Milano Bicocca
via Solferino 16, Monza I-20052, ITALY
E-mail: andreaalberto.lissoni@unimib.it

OCA
Henry Muggah, MD, FRCSC
Professor, Department of Obstetrics and Gynaecology, McMaster University, St. Joseph's Hospital
301 James Street South, 2nd Floor, Fontbonne Building, Hamilton, Ontario L8N 4A6, CANADA
Telephone: +1 905 521 6041 Fax: +1 905 521 6089
E-mail: muggah@mcmaster.ca

NIT
Dario Paladini, MD.
Professor, Obstetrics and Gynecology
Università degli Studi di Napoli "Federico II" (University Federico II of Naples)
Via Petrarca, 72, 81022, Napoli, ITALY
Telephone: +39 081 7462951 Fax: +39 081 7463255
Telephone: +39 339 2461688 (mobile)
E-mail: paladini@unina.it

UDI
Alberto Rossi, MD
Clinica Ostetrica Ginecologica
Università degli Studi di Udine
Piazza Misericordia, 33100 – UDINE – Italy
Telephone +39 349 5606530
E-mail: roalbert@tiscali.it

BIT
Luca Savelli, MD
Reproductive Medicine Unit, Department of Obstetrics and Gynecology
University of Bologna, Via Massarenti, 13, 40138 Bologna, ITALY
Telephone: +39 051 6364424 (work) Fax: +39 051 6360892
Via Mengoli 31/4, 40138 Bologna, ITALY (home) Telephone: +39 347 4248767 (mobile)
E-mail: savelliluca@libero.it E-mail: savelli@aosp.bo.it

RIT
Antonia Carla Testa, MD
Unità Operativa di Ginecologia Oncologica

Salvatore Mancuso, MD, PhD
Head of the Department of Obstetrics and Gynaecology
Istituto di Clinica Ostetrica e Ginecologica, Università Cattolica di Sacro Cuore
Largo Agostino Gemelli 8, Roma, ITALY.
Telephone: +39 06 30154979 Mobile: +39 339 7044256 Fax: +39 06 35510031
E-mail: atesta@rm.unicatt.it, foc.ovest@libero.it

LBE

Dirk Timmerman, MD, PhD
Professor and clinical head, Department of Obstetrics and Gynaecology,
University Hospitals KU Leuven,
Herestraat 49, B-3000 Leuven, BELGIUM.
Telephone: + 32 16 344215 (office) Fax: + 32 16 344205 (office)
E-mail: dirk.timmerman@uz.kuleuven.ac.be

Caroline Van Holsbeke, MD,
Department of Obstetrics and Gynaecology, University Hospitals KU Leuven
Herestraat 49, B-3000 Leuven.
Telephone: + 32 16 343642 (office) Fax: + 32 16 344205 (office)
E-mail: caroline.vanholsbeke@uz.kuleuven.ac.be

VIT

Diego Trio, MD
Dottor, Department of Obstetrics and Gynecology
Mauro Busacca, MD
Professor, Department of Obstetrics and Gynecology
Macedonio Melloni Hospital, University of Milan,
Via Melloni, 52, 20122 Milano, ITALY
Fax: +39 02 710645
E-mail: diego.trio@libero.it

MSW

Lil Valentin, MD, PhD
Professor, Obstetrics and Gynecology
Malmö University Hospital, SE20502 Malmö, SWEDEN. (Lövviksg 7B, S21374 Malmö)
Telephone: +46 40 949726 (home) Fax: +46 40 949726 (home)
Telephone: +46 40 332149 or 332094 (office) Fax: +46 40 962600 (office)
E-mail: lil.valentin@obst.mas.lu.se
E-mail: lil.valentin@med.lu.se

GBE

Caroline Van Holsbeke, MD
Willem Ombelet, MD, PhD, Head of Department
Department of Obstetrics and Gynaecology,
Ziekenhuis Oost-Limburg, Genk (ZOL)
Schiepse Bos 6, 3600 Genk, BELGIUM

Tel: +32 89 327524 Fax: +32 89 327920
Email: caroline.van.holsbeke@skynet.be

<u>OTHER CONTRIBUTORS</u>

Lieveke Ameye, M.Sc., PhD
Departement of electrical engineering (ESAT SCD-SISTA), K.U.Leuven
KasteelparkArenberg 10, B-3001 Heverlee-Leuven, BELGIUM.
Telephone: +32 477 794332 (mobile) Fax: + 32 16 321970 (office)
Email: lieveke.ameye@hotmail.com
Email: lieveke.ameye@esat.kuleuven.be

Anneleen Daemen, M.Sc.
Department of electrical engineering (ESAT SCD-SISTA), K.U. Leuven
Kasteelpark Arenberg 10, B-3001 Heverlee-Leuven, BELGIUM.
Telephone: +32 473 529251 (mobile) Fax: + 32 16 321970 (office)

Lieven De Clercq, M.Sc.
Department of electrical engineering (ESAT SCD-SISTA), K.U. Leuven
Kasteelpark Arenberg 10, B-3001 Heverlee-Leuven, BELGIUM.
Telephone: + 32 16 321143 (office) Fax: + 32 16 321970 (office)
E-mail: lieven.declercq@esat.kuleuven.be

Bart De Moor, M.Sc., PhD
Professor, Head of BIOI, Department of electrical engineering (ESAT SCD-SISTA), K.U. Leuven
Kasteelpark Arenberg 10, B-3001 Heverlee-Leuven, BELGIUM.
Telephone: + 32 16 321715 (office) Fax: + 32 16 321970 (office)
E-mail: bart.demoor@esat.kuleuven.be

Ben Van Calster, M.Sc., PhD
Department of electrical engineering (ESAT SCD-SISTA), K.U. Leuven
Kasteelpark Arenberg 10, B-3001 Heverlee-Leuven, BELGIUM.
Telephone: + 32 498 857512 (mobile) 32 16 321970 (office)
E-mail: ben.vancalster@esat.kuleuven.be

Sabine Van Huffel, M.Sc., PhD
Professor, Head of the BIOMED research group
Department of electrical engineering (ESAT SCD-SISTA), K.U. Leuven
Kasteelpark Arenberg 10, B-3001 Heverlee-Leuven, BELGIUM.
Telephone: + 32 16 321703 (office) Fax: + 32 16 321970 (office)
E-mail: sabine.vanhuffel@esat.kuleuven.be

Joan Veldman, MD
Erika Werbrouck, MD
Gynaecologists, Research fellows,
Department of Obstetrics and Gynaecology, University Hospitals KU Leuven,
Herestraat 49, B-3000 Leuven, BELGIUM.
Telephone: + 32 16 343642 (office) Fax: + 32 16 344205 (office)

E-mail: joan.veldman@uzleuven.be and erika.werbrouck@uzleuven.be

Ignace Vergote, MD, PhD
Professor, Head of Department of Obstetrics and Gynaecology, University Hospitals KU Leuven, Herestraat 49, B-3000 Leuven, BELGIUM.
Telephone: + 32 16 344636 (office) Fax: + 32 16 344629 (office)
E-mail: ignace.vergote@uzleuven.be

IOTA website : http://www.iota-group.org

Summary

The aim is to prospectively test and implement previously developed mathematical algorithms for the pre-operative classification of adnexal masses. IOTA phase 3 is a prospective multicentre study. The information obtained will be used to define the optimal management of patients presenting with adnexal tumours. 2,000 patients, with at least one adnexal mass, will be recruited and studied within 3 months before investigative surgery. Medical and family histories will be recorded. Transvaginal ultrasonography with colour Doppler imaging will be used to derive indices of tumour form and blood flow. A sample of peripheral venous blood will be taken for the analyses of serum CA-125 and other tumour markers and in certain centres additional blood will be taken and stored appropriately to test proteomic pattern analysis. Findings at surgery and the histological classification of excised tissues as malignant or benign (and by cell type) will be used as outcome measures. Conventional and novel algorithms (including proteomic patterns), which can be used to effectively classify difficult adnexal masses will be tested prospectively in centres throughout the world.

1. Study objectives

The main objective of this project is to improve preoperative diagnosis and subsequent management of patients with adnexal tumours by advanced algorithms in order to decrease morbidity and costs and in order to improve survival of patients with ovarian cancer. In particular, we aim to validate the added value of mathematical models as new diagnostic tool in the prediction of ovarian cancer in clinical practice. First of all, we aim to prove their enhanced diagnostic performance and generalized applicability as a first stage examination. In cases where prediction is unreliable, we aim to further improve the predictive performance of this diagnostic tool with second stage tests. As a side effect, a consistent large database of prospectively collected clinical, biochemical and ultrasound data will be generated for research into disease processes, as well as for the development and validation of other advanced algorithms.
To achieve these objectives the project is divided in work packages each having its own sub-objective.

1. Prospective external validation of predictive mathematical models and pattern recognition to distinguish between malignant and benign adnexal masses (Responsible person: D Timmerman; all centres)
2. Intravenous ultrasound contrast agents: quantitative analysis of contrast uptake and washout in tumour (Responsible person: A Testa; RIT, LBE, MSW, LSW,GBE, MIT, UDI). Not in UK centres.
3. Proteomic analysis (D Timmerman; only in LBE, MSW, GBE)
4. New set of tumour markers (D Timmerman; all centres with ethical approval and informed consent)
5. Validation of 3D power Doppler (L Valentin; all centres with GE Voluson equipment: LBE, GBE, SIT, LPO, MSW, RIT, CIT, KUK/UCH, NIT, MIT, LSW)
6. Validation of a new model, based on grey scale ultrasound and colour Doppler information, specifically designed for "difficult" tumours (all centres)

Schematic representation of the diagnostic algorithm applied in IOTA Phase 3:

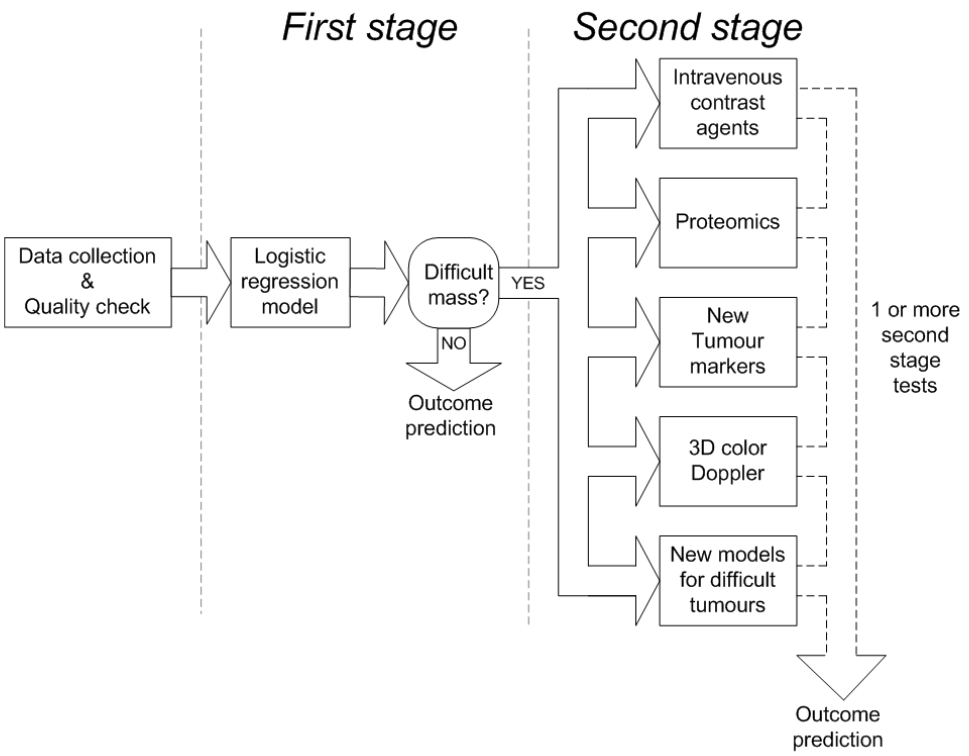

2. Background

At present malignant ovarian tumours are diagnosed at an advanced stage in 75 % of the cases and they result in the highest mortality figures of all gynaecological cancers. An estimated 22,430 new cases of ovarian cancer are expected to be diagnosed in 2007 in the United States, according to the American Cancer Society, with about 15,280 deaths. Worldwide there are more than 190,000 new cases of ovarian cancer each year, according to the International Agency for Research on Cancer. These figures underscore the need for effective tests to diagnose ovarian cancer at early stages and to improve management of patients with adnexal tumours.

The **IOTA study (International Ovarian Tumour Analysis)** is a multicentre collaborative project for the pre-operative characterisation of ovarian tumours based on predictive computer models.

The **first phase** of IOTA was conducted between 1999 and 2002. Several new mathematical models were developed based on the prospectively collected data of 1066 patients with a persisting adnexal tumour in 9 European Centres. Between 2002 and 2005 three centres continued the prospective collection in order to be able to perform an internal validation of mathematical models developed in IOTA phase 1. In this so-called IOTA **phase 1b** study a new dataset of 507 new patients was prospectively collected in 3 out of the 9 original IOTA centres. All models proved to perform excellent with area under the ROC curves of more than 0.94.

The **second phase** of IOTA consisted of an external validation of the models and this was conducted between 2005 and 2007. The diagnostic algorithms were prospectively validated on 2,093 patients with adnexal tumours in 19 centres in Belgium, Italy, UK, Sweden, Poland, Czech Republic, Canada, and China. A first analysis showed that overall performance of the logistic regression models was excellent (area under the ROC curve 0.94). A subgroup of “uncertain” tumours needs a reliable second stage test in order to help even experienced ultrasound examiners.

The **third phase** of the IOTA study is planned for 2009.

Rationale

Diagnostic and therapeutic relevance of predicting ovarian cancer preoperatively:

The pre-operative assessment of adnexal tumours remains a major challenge for clinicians. Advances in surgery have provided more treatment options, but their potential usefulness depends upon a prior assessment of the mass using non-invasive procedures. It is often difficult to determine pre-operatively the nature (benign or malignant) of ovarian tumours. However, this knowledge is essential for obtaining a reliable diagnosis and for an appropriate management, both of which will influence the outcome for the patient and the medical costs. In case of benign functional cysts a laparoscopy or any other surgical intervention should be avoided in order not to create unnecessary morbidity and impaired fertility. On the contrary, most benign non-functional tumours can be treated laparoscopically or with a low transverse abdominal incision. Patients with a suspect adnexal mass require a series of expensive and unpleasant staging examinations and in most cases also an exploratory laparotomy through a median incision is indicated, because the rupture of a stage 1 ovarian cancer during the operation may worsen the prognosis. This procedure should only be performed by a surgeon with proper skills and experience in debulking surgery, since the amount of residual malignant tissue after primary surgery is one of the most important prognostic factors in ovarian cancer. Furthermore, appropriate pre-, per-, and postoperative measures should be taken.

During recent years several techniques have been introduced to differentiate between benign and malignant ovarian lesions. These techniques include serum tumour markers, transvaginal ultrasonography with a variety of morphological scoring systems, or sometimes combined with colour Doppler sonography, computerised tomography (CT scan), and magnetic resonance imaging (MRI). Despite numerous publications on each of these techniques, an insufficient amount of data is available to provide for a rational clinical management.

Previous studies to distinguish between benign and malignant ovarian tumours were small and single-centre studies. The IOTA group first published a consensus statement on terms, definitions and measurements, which is now widely used. The IOTA phase 1 and phase 2 studies were by far the largest multicentre studies ever conducted in this area and they received international scientific attention and wide media coverage. The third phase is now needed to validate the use of second stage tests in order to provide reliable classifications in cases where the presently developed mathematical models result in uncertain diagnoses.

Official approval by the Ethical Committee

The multicentre project IOTA phase 3 will be submitted to the Ethical Committee of the University Hospitals Leuven as main investigating centre as well as in each participating centre.
Second stage tests are not uniformly performed in all centres and therefore each participating centre will provide appropriate patient information leaflets and request specific ethical approval to their Ethical Committee.

Insurance policy

This multicentre international study is initiated by the University Hospitals Leuven, Belgium. Each participating centre outside Belgium is fully responsible for optimal patient care and its own patient management in agreement with local laws. Each centre is also responsible for all legal aspects and for its own insurance of all matters related to this study.

Financial Support

The IOTA phase 3 project is supported by an Applied Biomedical Research grant (Toegepast Biomedisch Onderzoek, TBM) from the Flanders Institute for Scientific and Technological Research: IWT Flanders, Belgium (IWT-TBM 070706). This grant covers costs of central data collection, proteomic analysis, analysis of new tumour markers and statistical analyses.

There is no financial recompensation for principal investigators nor patients.

3. Design

Number of patients / tumours

2,000 patients with at least one histologically examined adnexal mass will be recruited for the third part of the IOTA trial (i.e. the prospective testing of new mathematical models and second stage tests).

Outcome measures

The histological classification of removed tissue and the findings at surgery.

Duration

In **year 1**, the IOTA phase 3 study protocol will be implemented in the different participating centres. Data collection with minor built-in quality checks will occur similarly as in IOTA phase 2. At the end of year 1, complete data of 1,000 patients will be available.
In **year 2**, data collection will continue until the target sample size of 2,000 patients is reached. At the end of data registration, a detailed data quality control will be applied. All mathematical models developed in IOTA phase 1 and 2 will be prospectively validated as 1st stage test. At the end of year 2, the predictive performance of two 2nd stage tests in case of difficult tumours will also be validated: 3D power Doppler and a logistic regression model using grey scale ultrasound that was specifically constructed for difficult tumours.
In **year 3**, the validation of the three remaining 2nd stage tests will be investigated: intravenous ultrasound contrast agents, proteomic analysis and new tumour markers. During the third year different publications each highlighting different second stage tests will be prepared as well as overall study reports in high impact medical journals (e.g. JCO, J Natl Cancer Inst…)
No problems are expected with the data collection, data quality control and data storage, and data analysis since these will be similar to the successful IOTA phase 2 study and because of the existing expertise in the IOTA group.

4. Patient entry

Inclusion criteria

All patients assessed with transvaginal ultrasound by the principal investigators (or their appropriately trained medical staff) and found to have an apparent persistent extrauterine pelvic mass* (from this point termed: mass), provided that

1. the patient is fit for surgery
2. the patient gives informed consent
3. the patient has at least one remaining ovary

- *: a persistent extrauterine pelvic mass is defined as a mass judged by ultrasonography to be of adnexal origin, and not consistent with normal physiology
- Patients with bilateral tumours will be included in the study. Both tumours are examined. The worst case mass based on morphological characteristics (according to the sonographer) is recorded first (a list of examples is attached). If both masses are morphologically equal, the largest one is recorded first.

Exclusion criteria

- Patients with more than one lesion in the same ovary or in two different ovaries will be included in the data collection, but in case of two different non-physiological conditions (based on the pathology report) the results will be excluded from the main analysis of the data. The reason for this approach is to avoid several difficulties: e.g. to assess the Doppler signals of different masses separately, and to know the contribution of each mass in particular elevation of serum CA 125 levels).
- Pregnancy is not an exclusion criterium, but the data from pregnant patients are analysed separately, because the physiology is different (e.g. different colour Doppler findings and higher serum CA 125 levels).
- Preferably surgery is scheduled within one week after ultrasonography. If more than 90 days have passed before the patient has been operated upon, the patient is excluded for analysis, except if a new scan has been performed prior to surgery.

Consent / information leaflet

Information leaflets are at the discretion of the participating centres.
Approval of the local Ethical Committee for clinical studies is necessary.
Written informed consent is necessary for storing blood samples for later analysis, and for the intravenous contrast study.

Collection of clinical data

Family history:	Number of first degree relatives with ovarian cancer (0-...)
	Number of first degree relatives with breast cancer (0-...)
Medical history:	Personal history of ovarian or breast cancer
	Age (years); Parity (number of deliveries)
	Hysterectomy (yes/no)
	Menopausal status (pre-(1) or postmenopausal (3))
	Years after menopause;
	Day of cycle. Hormonal therapy (yes, no).
	Pelvic pain during the scan: “is the mass painful?” (yes/no)

5. Diagnostic methods

Ultrasonography

Ultrasound variables / definitions

All ultrasound variables are included in the dedicated software, which shows the requested parameters. In the database 0 always means NO and 1 always means YES.
The adnexal lesion is that part of an ovary or of an adnexal mass that is judged by ultrasonography to be not consistent with normal physiology. This can be a persistent unilocular cyst, surrounded by normal looking ovarian stroma with some follicles. In this case the whole ovary containing the cyst is the ‘ovary’, whereas the unilocular cyst is the ‘lesion’. Both are measured and the cyst is described as being ‘unilocular’ and not ‘unilocular-solid’. In other cases the lesion is separate from the ovary (e.g. hydrosalpinx). Again, both ovary and lesion are measured separately. In other cases no normal ovarian stroma is seen. In these cases the lesion and the ovary are undistinguishable and the measurement of lesion and ovary will be the same.

Measurements (in mm): The ovary in two perpendicular planes
The lesion in two perpendicular planes
The volume of the tumor is calculated from the three diameters in two perpendicular planes

- The presence of ascites (*i.e.fluid outside* the pouch of Douglas) is noted (yes/no).
- Fluid in the pouch of Douglas is measured in a sagittal plane (the largest anteroposterior diameter is given).
  (see Figure)

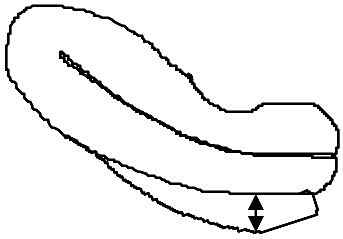

- A septum is defined as a thin strand of tissue running across the cyst cavity from one internal surface to the contralateral side. The thickness of the thickest septum is measured where it appears to be at its widest (other than at its interface at the internal surface of the cyst wall)

It is preferable to measure a septum which is perpendicular to the ultrasound beam

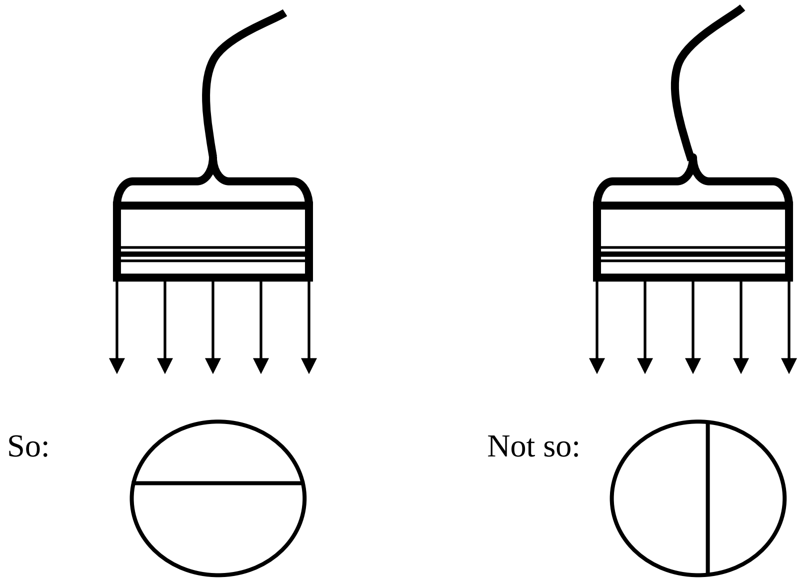

- An incomplete septum (as seen in hydrosalpinges) is defined as a thin strand of tissue running across the cyst cavity from one internal surface to the contralateral side, but which is not complete in some scanning planes. Its presence is noted. If a cyst only has incomplete septa and no real septa, it is unilocular, despite the fact that in certain sections the cyst appears to be multilocular.
- Solid means echogenicity suggesting the presence of tissue (e.g. the myometrium, the ovarian stroma, myomas, fibromas). Methods to distinguish between blood clots and the presence of solid tissue are the use of colour Doppler and to look for internal movement when gently pushing to the structure with the transducer. The presence of flow (with the appropriate settings) is diagnostic for solid tissue. The absence of flow is not informative. In cases of doubt whether it is a blood clot or a solid area, call it solid.
- Solid papillary projections are defined as any solid projections into the cyst cavity from the cyst wall greater than or equal to 3 mm in height

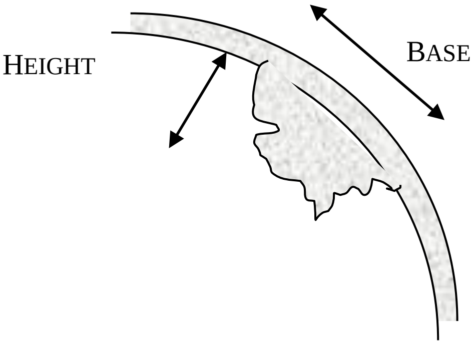

If it is unsure whether solid papillary projections or an incomplete septum are present, the 'worse case scenario' is used. E.g. 'cogwheel excrescences' and 'beads-on-a-string' (as seen in hydrosalpinges) should be classified as papillary excrescences if their height is greater than or equal to 3 mm. The 'white ball' in a dermoid, however, should not be classified as a solid papillary projection.

The 'sludge' on the internal walls of endometriotic cysts is not regarded as a papillary projection. In these cases the internal walls are usually 'irregular'.

- The number of separate papillary projections is noted (1/2/3/more).
- The presence of flow within some of these projections is noted (yes/no).
- Solid papillary projections are described as being 'smooth' or 'irregular' (e.g. cauliflower-like).

In some cases it is difficult to judge whether it is a papillary projection and from which point to measure the projection. In these cases it may be helpful to use an imaginary line as shown in the following schematic drawing:

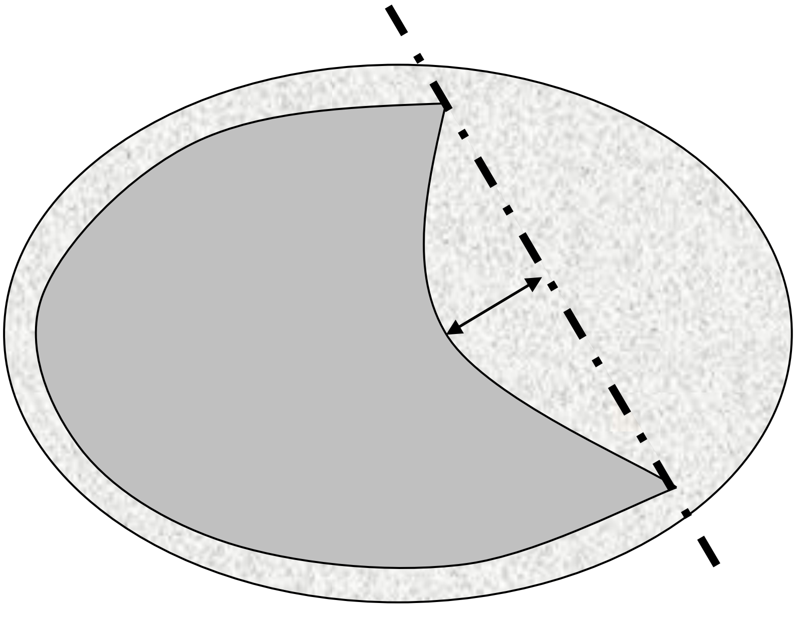

All lesions are qualitatively classified into one of 5 categories:

1. unilocular (a unilocular cyst without septa and without solid parts or papillary structures). Normal ovarian stroma is not regarded as ‘solid’ (e.g. a peritoneal cyst, containing a normal ovary, is unilocular and not unilocular-solid).

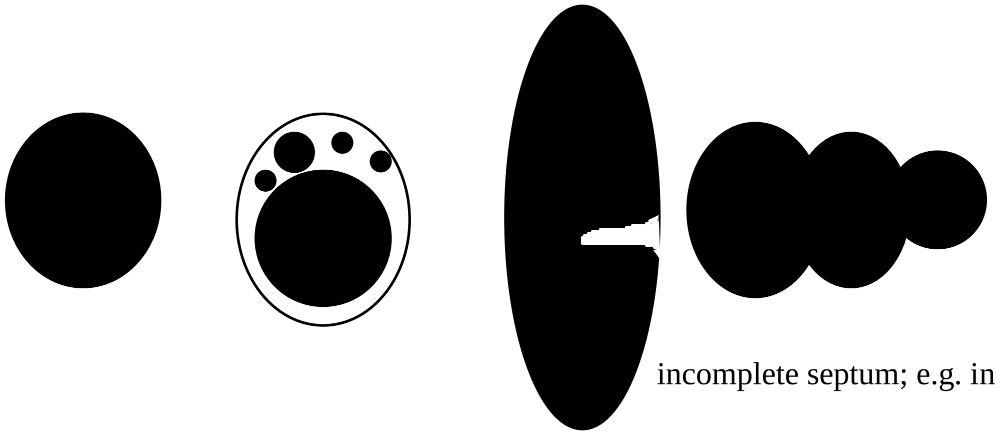

incomplete septum; e.g. in hydrosalpinx)

2. unilocular cyst with solid component (a unilocular cyst with a measurable solid component or at least one papillary structure). This category may include pyo- or hydrosalpinges with the so-called ‘beads-on-a-string’ or ‘cogwheel’ appearance if ≥ 3 mm. If the solid part contains very small cysts the mass might be unilocular-solid (see below).

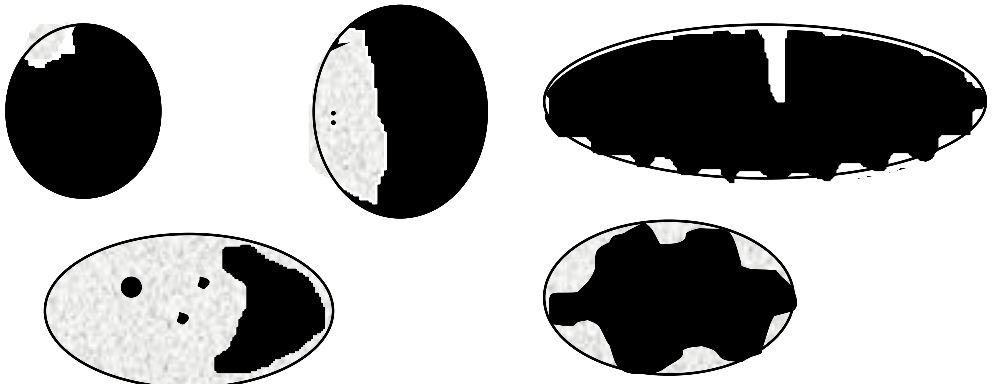

3. multilocular (a cyst with at least one septum but no measurable solid components or papillary projections). The ‘lesion’ is measured as indicated by the arrows.

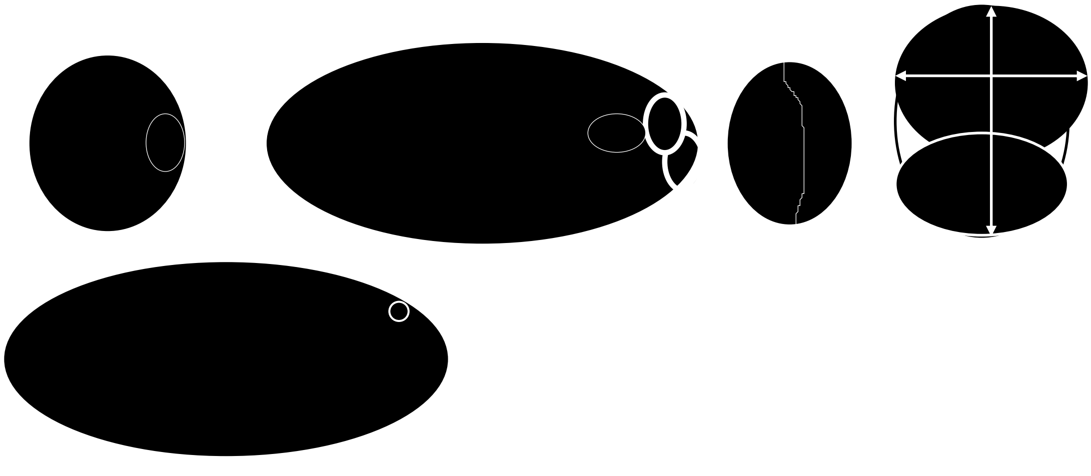

4. <u>multilocular with solid component</u> (a multilocular cyst with a measurable solid component or at least one papillary structure)

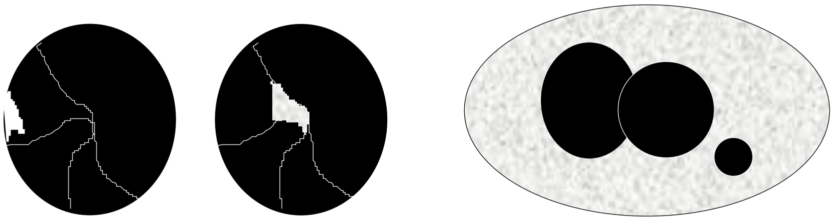

5. <u>solid</u> (a tumour where the solid components comprise 80% or more of the tumour when assessed in a two-dimensional section).

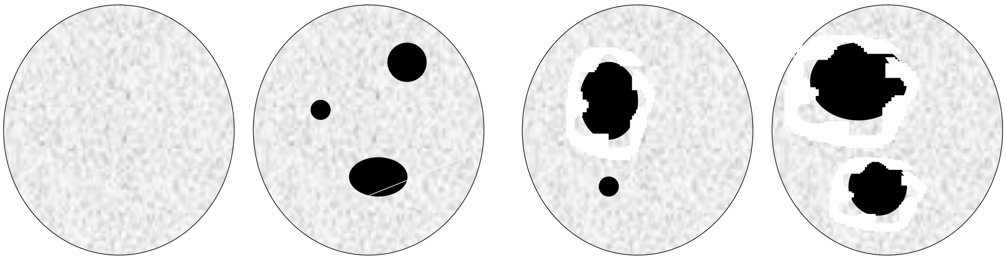

(solid tumour with an irregular cyst wall)

A solid tumour may contain papillary projections protruding into the small cysts.

6. <u>not classifiable</u> because of poor visualization (e.g. strong acoustic shadowing due to calcifications or as seen in certain dermoids *('tip of the ice-berg' sign)*

QUANTITATIVE ASSESSMENT OF MORPHOLOGY

- In cystic-solid tumours the largest solid component is measured separately (in three perpendicular planes). The solid component is noted as being smooth or irregular (e.g. cauliflower-like). In some cases a solid papillary projection is the largest solid component and thus the papillary projection is recorded both as papillary projection and as solid component.
- The internal wall is also noted as being smooth or irregular.

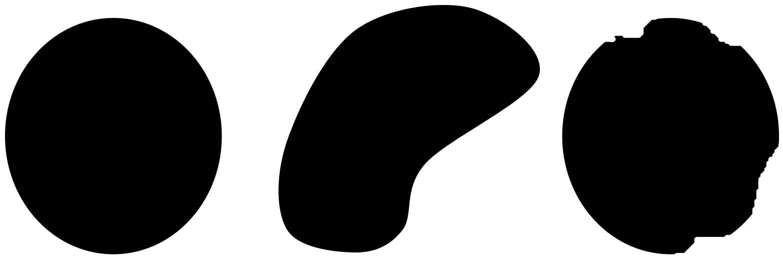

Smooth Smooth Irregular

If there is a solid papillary projection, then the wall is irregular by definition.

- The external wall of cyts is not looked at.
- In cases of solid tumours the description of the internal wall being smooth or irregular is usually not applicable but the outline of the tumour is described as smooth or irregular.
- If there is any irregularity in either the inner wall of any cyst or in the outer wall of a solid tumour or on the surface of a solid component, the lesion is described as ‘irregular’.

- The dominant feature of the cystic contents is described as anechoic (black), low-level echogenic (homogeneous low level echogenic as seen in mucinous tumours), 'ground glass' appearance (homogeneously dispersed echogenic cystic contents, as often seen in endometriotic cysts), hemorrhagic (with internal thread-like structures, representing fibrin strands; it is possible to describe the echogenicity as star-shaped, cobweb-like or jelly-like) or mixed echogenic (as often seen in teratomas) (see images attached).

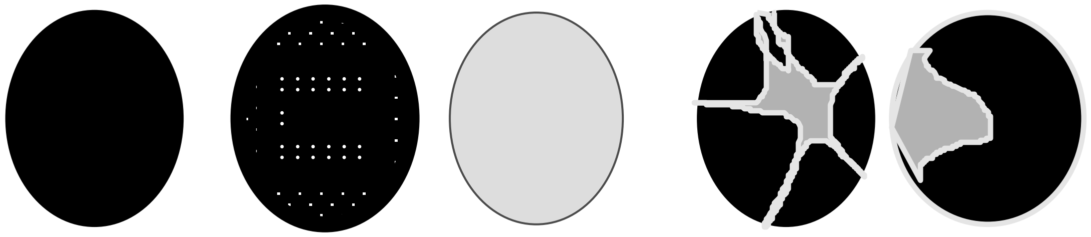

Anechoic   Low level   Ground glass   Hemorrhagic   Hemorrhagic

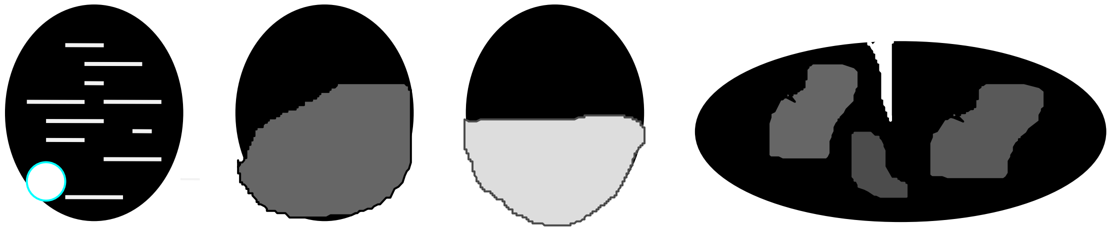

Mixed   Mixed (old blood-fluid level or fat-fluid level)   Mixed (e.g. abscess)

- The presence of acoustic shadows, defined as loss of acoustic echo behind a sound-absorbing structure, is noted as well. Solid tumours are identified by the appearance of the internal texture, by the absence of internal movement when moving the transducer or by colour Doppler imaging (presence of central flow).
- In solid tumours the dominant feature of any cystic contents is described only if it can be assessed.
- Acoustic streaming, defined as the bulk movement of fluid due to a sound field. It results in movement of fluid particles in the direction of the ultrasound beam away from the transducer ("absent" or "present", mandatory new variable for phase II)
- 'Ovarian crescent sign', defined as the presence of normal ovarian tissue adjacent to an adnexal tumour. ("absent" or "present", mandatory new variable for phase 3)
- Ultrasound evidence of metastases (e.g. "omental cake" or peritoneal tumoural implants). ("absent" or "present", mandatory new variable for phase 3)

Colour Doppler imaging and blood flow indices

Subsequently, the entire tumor is surveyed by CDI. The power, gain and pulse repetition frequency are initially adjusted for maximum sensitivity of low blood flow states. The lowest velocity signals are filtered out by gradually increasing the pulse repetition frequency and flow analysis is concentrated on the highest velocity signals. A subjective semiquantitative assessment of the amount of blood flow (area and colour scale) within the septa, cyst walls, or solid tumor areas is made: a score of 1 is given when no blood flow can be found in the lesion; a score of 2 is given when only minimal flow can be detected; 3 is given when moderate flow is present and 4 is given when the adnexal mass appears highly vascular with marked blood flow using colour Doppler. This colour score refers only to colour Doppler image and not to Doppler shift spectrum. It is only given once (for the tumour as a whole). Multiple photographic prints are made of relevant structures and Doppler signals.

Quality control

Several informative images of all adnexal masses should be made. Preferably, these are stored digitally. Photographs or video are acceptable as well.

<u>6. Subjective assessment</u>

After ultrasonographic examination of the mass the investigator gives his subjective assessment of the mass:

A: Malignant (1) or benign (0)?
B: Probability of malignancy: 1 = benign
(=level of certainty) 2 = probably benign
3 = uncertain
4 = probably malignant
5 = malignant
C: Self impression: presumed histological diagnosis (e.g. dermoid, serous cystadenoma, endometrioma…)

<u>7. Serum tumour markers</u>

Sample collection / treatment / storage

Ideally, CA 125 measurements are centralised, using the CA 125 II immunoradiometric assay (Centocor, Malvern, PA) on frozen samples, but we accept local measurements of serum CA 125 levels if it is difficult to store serum samples.
In some centres additional blood samples will be taken for use in proteomics pattern analyses. Written informed consent will be obtained for this purpose.

<u>8. Histopathology and staging</u>

<u>Surgical investigation</u>

Surgery is indicated in case of a persistent mass after 6-12 weeks. In cases of symptomatic adnexal masses, suspected malignancy or at patient’s request immediate surgery may be performed. Premenopausal patients with a persistent unilocular smooth cyst < 5 cm and postmenopausal patients with a persistent unilocular smooth cyst < 3 cm may be suitable for follow-up.
Surgery is performed either laparoscopically or transabdominally, depending on the surgeon’s clinical judgement. Cytology of cyst fluid or fine needle biopsies are not sufficient for histological classification. If cyst fluid has been obtained, the cytological classification is noted.

Tissue collection

Preferably the whole tumour should be removed. However, representative biopsies may be sufficient (e.g. in advanced ovarian cancer or endometrioma).

Sampling

All surgically removed tumours should be extensively sampled for histological examination. The number of prepared blocks is dependent of the size and the nature of the tumour.

Tumour classification

Tumours are classified according to the criteria recommended by the International Federation of Gynecology and Obstetrics (FIGO). In malignant tumours the degree of differentiation is included.

9. Data collection

Clinical report forms: not necessary, because the dedicated software in Astraia (www.astraia.com) will be used for direct retrieval of the collected data.

10. Statistical analysis

New mathematical models have been constructed in IOTA phase 1 and 2. In phase 3 these models will prospectively be evaluated in terms of sensitivity and specificity and overall accuracy (i.e. the proportion of the sum of true positives and true negatives over the total number of cases). Independent risk factors for malignancy will be expressed as odds. The models will be compared by constructing receiver operating characteristic (ROC) curves.
In addition new models based on new variables will be constructed. The data will be thoroughly inspected using univariate analyses (contingency tables and basic descriptive statistics) and multivariate analyses (principal component analysis, biplots, scattermatrices, canonical correlation analysis and logistic regression) to explore the data and to detect multicollinearity and outliers. Manual checks of any outliers will be performed to eliminate mistakes that had occurred during submission of the data.

11. Study supervision

Central supervision: the Steering Committee is responsible for the protocol, quality control, interim analyses of the data and final analysis and reporting of the study.

Local supervision: the Principal Investigators are responsible for the data collection in their centres.

Dirk Timmerman is responsible for the co-ordination of the IOTA project and the contact between the centres.

Sabine Van Huffel and Bart De Moor are responsible for the data management and the development of new algorithms, in collaboration with B. Van Calster, L. Ameye, A. Daemen O. Gevaert, V. Van Belle, L. De Clercq, P. Antal, and J. Vandewalle.

12. Publication policy

The steering committee is responsible for publication of the data in scientific journals. As such the members are co-authors in all resulting clinically relevant papers, to which they made significant contributions. By the time of the final analysis the principal investigators have to have contributed at least 50 cases to the study. They are co-authors, according to the number of patients they contributed to the study (depending on the journal's restriction of the number of co-authors).

Purely mathematical papers without clinical relevance related to the study data are published by S. Van Huffel, B De Moor and co-workers at ESAT with reference to the IOTA group and the inclusion of as many as possible of the clinical contributors.

The Katholieke Universiteit Leuven represented by its department K.U.LEUVEN RESEARCH & DEVELOPMENT, having its office in 3000 Leuven, Minderbroedersstraat 8A – box 5105, Belgium, VAT number BE 419.052.173 holds intellectual property rights that might result from the IOTA 3 project.

Detailed Research Description

State of the art in the literature

It is of the utmost importance to have preoperatively a correct idea of the benign or malignant character of an adnexal mass because this will influence not only the whole treatment strategy but also the prognosis of the patient [1]. If an adnexal mass is most likely to be benign we could suffice with minimal invasive surgery or even conservative treatment with no need for referral to a gynaecologic oncologist. In this way the duration of hospitalisation and revalidation will be limited and morbidity will be minimal in comparison with surgery after midline laparotomy [2,3]. On the other hand, whenever a mass is considered to be malignant, laparoscopic surgery is contra-indicated because spilling of the cyst content during surgery may worsen the prognosis of the patient [1] and the patient should be referred to a gynaecologic oncologist for proper staging and debulking of the tumour [4].

O1: **Prospective external validation of predictive mathematical models and pattern recognition to distinguish between malignant and benign adnexal masses**

In the past several scoring systems and mathematical models using ultrasound variables have been developed for the preoperative prediction of probability of malignancy of an adnexal mass. However, before these models can be used in daily practice, they should be tested prospectively on a new population. Some models have been tested prospectively on small data sets, but results were disappointing [5-9]. We recently published the results of prospective testing of 17 scoring systems and mathematical models for the calculation of the risk of malignancy in adnexal masses on a large dataset collected by the International Ovarian Tumour Analysis (IOTA) collaborative group, the IOTA phase 1 dataset [10]. Unfortunately, most models and scoring systems performed worse than was originally reported. Of the 17 models tested, only the previously published neural networks, the vector machine models and one of the logistic regression models had good performance on prospective testing [10].
The primary aim of the IOTA group was to prospectively collect clinical information and standardised data from ultrasound examinations in a large number of patients with adnexal masses in order to create new scoring systems and mathematical models to distinguish between benign and malignant adnexal tumours. The multicentre approach was chosen as the most likely method to achieve a model which might be more effectively applicable to prospective studies in different clinic populations.

O2: **Validation of intravenous contrast agents: quantitative analysis of contrast uptake and washout in tumour**

Accurate preoperative diagnosis of ovarian tumours is essential for planning appropriate patient management and improves patient outcome if a malignancy is present. Transvaginal ultrasonography is at present the most effective method for early diagnosis of ovarian tumours, with a sensitivity of 80-85%. A characteristic of malignant ovarian tumours is the presence of neovascularisation which allows the tumour to grow. Changes in vessels may be visualised before tumour detection. An ultrasound technique that can be used to describe ovarian vascularisation is colour Doppler sonography [5,11-14]. In recent years, the depiction of intratumoural vessels with colour and power Doppler sonography has improved, but visualisation of vessels smaller than 0.1 mm in diameter remains impossible. The use of intravascular contrast agents increases the signal-to-noise ratio, resulting in an improved detection of low-volume blood flow. In this way signals from vessels less than 200 μm in diameter can be depicted. Orden et. al [15] showed a difference in degree, onset and duration of Doppler US enhancement between malignant and benign adnexal lesions after the injection of microbubble contrast agents.

O3: **Validation of proteomic analysis**

Since ovarian cancer is clinically quiet, it is often called the “silent killer”. Therefore ovarian cancer diagnosis would greatly benefit from more advanced technologies that can be applied for diagnostic purposes. In 2002 Petricoin et al. [16] applied Surface-Enhanced Laser Desorption and Ionization (SELDI) mass spectrometry to assess differences in the spectra of benign and malignant ovarian masses. They used 50 serum samples with ovarian cancer and 50 serum samples without ovarian cancer and used an iterative search algorithm to develop a

pattern that distinguishes benign from malignant. Next, this model was tested on 116 new serum samples, 50 from women with ovarian cancer and 66 from unaffected women or non-malignant ovarian masses. Using SELDI mass spectrometry this resulted in a pattern based on the amplitudes at key mass-to-charge (m/z) values of 534, 989, 2111, 2251 and 2465. This pattern had a sensitivity of 100% and specificity of 95% with a positive predictive value of 94%. However, no identification of the key m/z values was performed, therefore this pattern constitutes a black box model (i.e. no biological interpretation is possible). The authors also claimed that this model can be used for ovarian cancer screening.

The results of this study lead to a significant increase in research on mass spectrometry-based proteomics applied for cancer diagnosis and prognosis. However, the approach of Petricoin et al. [16] is highly questionable [17] and initiated a debate in the literature [18-24]. These concerns show that the approach by Petricoin and colleagues has several disadvantages and may not be ready for clinical use. However, mass spectrometry-based technology should definitely not be dismissed as an interesting concept [25] and has several advantages when more effort is invested in the use of high quality instrumentation and processing. The use of more advanced technologies such as high pressure liquid chromatography and MALDI-based mass spectrometry can deliver substantially better sensitivity and specificity, with the added possibility of obtaining more biological insight via protein identification.

O4: **Validation of a new set of tumour markers**

Serum CA 125 is a glycoprotein and at present it is the tumour marker with the highest sensitivity for ovarian cancer. Recently different other tumour markers for diagnosis of ovarian cancer have been discovered. Some of the most promising include CA 15-3, CA 72-4, CEA, macrophage colony-stimulating factor (M-CSF), HE4, mesothelin, osteopontin, kallikrein(s), and soluble EGF receptor. However, external validaton is needed to test the value of these and other markers in patients with adnexal tumours [26].

O5:**Validation of 3D power Doppler**

Several strategies can be followed for the preoperative sonographic assessment of adnexal masses. In general, the first step is the ultrasonographic evaluation using subjective evaluation or pattern recognition. In the past, some studies have shown that expert sonologists can reach a sensitivity of 98% and a specificity of 90% solely by using their subjective evaluation [27-28]. Unfortunately, this is not the performance of the less experienced sonographer [27]. A number of different scoring systems and mathematical models have been developed to help the less experienced sonographer in the discrimination between the benign or malignant character of an adnexal mass [29-33]. The first models only used grey scale ultrasound variables. Later on 2D colour Doppler became available and several studies investigated the benefit of this new technique. A disadvantage of the technique is that it is highly dependent on the ultrasound equipment that is used and the blood vessel that is interrogated. The first reports on velocity and resistance indices were promising but later on it became clear that there was too much overlap between benign and malignant masses [34-35].

In a study from Valentin et al. where 173 cases were examined using grey-scale and colour Doppler examination, only 5 extra cases were classified correctly after the use of colour Doppler [36]. Probably this is also one of the reasons that mathematical models that included these Doppler parameters did not perform well when tested prospectively [10,37].

Advantage of 3D Power Doppler examination in comparison with 2D power Doppler examination. Introduction of three-dimensional (3D) power Doppler ultrasound has made it possible to assess vascularisation in a whole organ or tumour in a more objective way.

The vascularisation index (VI) is the ratio of colour voxels to all voxels in the region of interest expressed as a percentage. It reflects the density of vessels in the volume analyzed. The flow index (FI) is the sum of weighted colour voxels divided by the number of all colour voxels in the region of interest, and it reflects the number of blood corpuscles in the vessels of the volume. The vascularisation-flow index (VFI) is the sum of weighted colour Doppler voxels divided by all voxels in the region of interest. It reflects both the density of vessels and the number of blood corpuscles flowing in the vessels of the volume.

Another opportunity is to be able to look at the vascular tree of a tumour. Some authors showed that the morphology of vessels is different in benign vs. malignant tumours. Vessels in a malignant tumour tend to be more branched and tortuous [38]. Although several reports have showed that 3D power Doppler can quite reliably

distinguish between benign and malignant masses [13,39-41], Jokubkiene et al. and Guerriero et al. demonstrated that 3D power Doppler added little to the correct diagnosis of malignancy in an unselected ordinary population and was not superior to grey-scale imaging [42-43]. Jokubkiene et al. compared the performance of logistic regression models using grey-scale variables only, subjective evaluation and logistic regression models that added 3D power Doppler variables as well. These data showed that 3D power Doppler added little benefit to the use of grey scale only [42].

O6: **Validation of a new model, based on grey scale ultrasound and colour Doppler information, specifically designed for "difficult" tumours**

An experienced ultrasound examiner using a good ultrasound system can be expected to correctly discriminate between benign and malignant adnexal masses in 9 of the 10 cases [5,27,28]. The reported sensitivity and specificity with regard to malignancy in the studies cited were around 90%. For less experienced operators, the sensitivity and specificity were 86% and 80% [27]. The main IOTA logistic regression model applicable to all tumours [44] could be of considerable help to these less experienced clinicians, but does not outperform experienced ultrasonographers. But in approximately 1 in 10 cases even an experienced ultrasound examiner is likely to fail to make a confident and correct diagnosis [45]. Until now, no successful model has been constructed in these "uncertain" cases. Before a method capable of distinguishing between benignity and malignancy in such difficult pelvic masses is found (the new method might next to grey scale ultrasound or colour Doppler also involve ultrasound contrast (O2), proteomics (O3), new tumour markers (O4) or 3D power Doppler (O5)), we must accept that some women will need to undergo an unnecessary operation – or perhaps an unnecessarily extensive operation – because of our inability to reliably exclude malignancy before surgery.

State of the art in the consortium and comparative advantage

O1: **Prospective external validation of predictive mathematical models and pattern recognition to distinguish between malignant and benign adnexal masses**

IOTA phase 1. During IOTA phase 1 we collected more than 50 ultrasound, demographic and clinical variables of 1,066 patients with an adnexal mass. A histopathologic diagnosis was available for all patients. Patients were collected in 9 different international centres where the ultrasound examination was performed by one of the IOTA expert sonologists following a standardised protocol. In this way we obtained a large and multicentre database that is more likely to resemble the general population [44].
Based on this dataset 12 mathematical models were developed (2 logistic regression models [44], 1 scoring system (unpublished data), 3 neural networks [46] and 6 kernel based models [47] (3 support vector machine models (SVM) and 3 relevance vector machine models (RVM)). On the test set of IOTA phase 1 all models obtained an area under the ROC curve (AUC) of more than 0.92.
IOTA phase 1b. In IOTA phase 1b a new dataset of 507 new patients was prospectively collected in 3 of the 9 IOTA centres. All models proved to perform excellent with again AUCs of more than 0.94 (unpublished data).
IOTA phase 2. The aim of IOTA phase 2 was to test the IOTA models in new centres with different population characteristics and different levels of ultrasound experience. More than 2,000 patients have been included in 20 centres throughout the world. In a preliminary analysis the main logistic regression model (1) was tested and in every centre the AUC was above 0.87. When tested on the whole dataset the AUC was above 0.93. In IOTA phase 1 and 2 we found that both for the expert sonologist using pattern recognition (subjective impression) as for the mathematical models, approximately 8% of the adnexal masses were difficult to classify as benign or malignant and lied close to the decision boundary. Models that were built especially for the classification of these difficult masses showed disappointing results [45].
IOTA phase 3 . During IOTA phase 3, the same centres that participated in IOTA phase 2 will prospectively include a new dataset of more than 2,000 patients but will now be able to evaluate the prediction of the main logistic regression model immediately after performing the ultrasound scan.

O2: **Intravenous contrast agents: quantitative analysis of contrast uptake and washout in tumour**

We evaluated the efficacy of contrast-dedicated ultrasound technology, contrast-tuned (CnTI) imaging and using the second-generation contrast agent SonoVue (Bracco International BV, Amsterdam, the Netherlands), in

comparison with the standard ultrasound examination in ovarian tumours [48-49]. Eighty-nine patients were enrolled in the study in 4 different clinical centres. The study included 40 uncertain pervic adnexal masses, 10 pelvic masses indicative of recurrence of gyneacologic tumours, 26 uterine pathologic features and 13 cervical lesions. Pictures of the intralesional microvascularization after SonoVue injection differed dramatically from those obtained during colour Doppler examination. By use of the CnTI technology, it was possible to improve the ability of the operator to distinguish benign from malignant lesions.
Preliminary results indicate an area under the ROC curve (AUC) of 0.90 for peak intensity of contrast enhanced signals as opposed to 0.79 for subjective assessment to discriminate between malignant and benign or borderline tumours. An AUC of 0.85 and 0.82 is obtained to separate benign from malignant and borderline tumours for the area under the intensity curve and subjective assessment, respectively. Within this project the preliminary results can be checked on a larger sample set, permitting to fine-tune the decision thresholds and to create more complex models.

O3: **Validation of proteomic analysis**

Pilot study. We adopted the MALDI approach to benefit from the potential diagnostic power of mass spectrometry-based proteomics. We have started a pilot study consisting of 39 serum samples from 20 patients with benign ovarian masses and 19 patients with malignant ovarian masses. The patients in the malignant group consist of 3 stage I tumours, 15 stage III tumours and 1 stage IV tumour. In this first phase the goal is to detect interesting m/z ranges that display differential patterns in benign and malignant ovarian cancer (PILOTMODEL). Subsequently, a second phase consisting of identification of proteins and/or peptides within these mass ranges will be initiated. The use of MALDI-TOF offers a wide range of advantages compared to the SELDI platform described earlier. It provides a better resolution and wider mass range compared to the SELDI platform. Secondly MALDI offers the possibility of identifying interesting peaks at key m/z values by subsequent fragmentation which is impossible with SELDI. The resulting mass spectral patterns can be submitted to mass spectrum interpretation engines such as MASCOT for comparison with known proteins and peptides in databases, returning the most probable biomolecule responsible for the mass spectral measurement.
Proteomics as second stage test: innovative aspects. In this project we aim to expand the pilot study: firstly, by increasing the number of patients analysed with mass spectrometry as a second stage test and secondly, by validating the PILOTMODEL on a larger set of samples. This entails the following innovative aspects: Firstly, by increasing the number of patients analysed more robust analysis can be performed. In both benign and malignant ovarian tumours different subgroups exist. By expanding the number of samples analysed, these effects can be taken into account to look for proteins and/or peptides which are present in all benign samples and absent in all malignant samples and vice versa. Secondly, the MALDI-TOF/TOF work-flow that was applied in the pilot study and that will be used in this project, has not been previously used in this context. Finally, SCD/BIOI has gained much experience in the analysis of high dimensional data, both in pre-processing as in the actual modelling. Therefore, the most recent advances in mathematical modelling in medical informatics and bioinformatics will be used to develop mathematical models that can discriminate between benign and malignant ovarian cancer. For this purpose SCD/BIOI can rely on previously developed models and techniques on clinical and microarray data.

O4: **Validation of a new set of tumour markers**

We prospectively assessed the value of serum CA-125 [50-51]. These studies challenged the current clinical practice, where CA-125 is an integral part of the preoperative work up of patients with adnexal masses. These studies proved for the first time that a single measurement of serum CA-125 does not add to the diagnostic confidence of experienced ultrasound examiners nor to the diagnostic performance of logistic regression models to distinguish between benign and malignant tumours. Therefore, we need to test combinations of new tumour markers that might really improve the classification of difficult tumours. A pilot study on proteomics has been performed in our consortium and this was first presented at the ESGO 2007 meeting (European Society of Gynaecologic Oncology) [52]. External validaton is now needed to test the value of combinations of novel tumour markers with clinical and ultrasound variables in other patients with adnexal tumours.

O5: **Validation of 3D power Doppler**

2D pulsed Doppler information did not improve mathematical models developed to distinguish between benign and malignant adnexal masses. In the already validated IOTA models none of the velocity or resistance Doppler parameters were selected as independent variables [10,31,44,46,47]. Only the colour score showed to be an independent variable. This is a semiquantitative score between 1 and 4 that gives the score of 1 when the tumour shows no vascularisation and 4 if the tumour is highly vascularised [12]. The drawback is the fact that the 2D power Doppler ultrasound result is related to the blood vessel that is examined. It does not offer the investigator an overall image of the vascularization in the tumour, this in contrast to 3D power Doppler. Several IOTA group members published papers on the use of 3D power Doppler to distinguish between benign and malignant adenexal tumours [42,43]. Therefore, 3D power Doppler ultrasound will be offered as a second stage test to the investigators in order to assess the added value of this examination to the clinician's preoperative diagnosis.

O6: **Validation of a new model, based on grey scale ultrasound and colour Doppler information, specifically designed for "difficult" tumours**

Using subjective evaluation of grey scale and Doppler ultrasound findings, an experienced ultrasound examiner using a good ultrasound system can correctly classify ovarian tumours as benign or malignant in most cases. The experts reached in IOTA 1 and IOTA 1b a sensitivity of 88% (234/266) and 90% (129/143), respectively, and a specificity of 95% (762/800) and 93% (338/364) ([44], unpublished data). In 8% of the cases, the ultrasound examiner found it difficult to discriminate between benign and malignant tumours (90/1066 in IOTA 1, 39/507 in IOTA 1b) [45]. Borderline tumours were over-represented among these "uncertain" masses, being three times more common among the "uncertain" masses than among the other ones. Of those borderline tumours, 60% (33/55) were correctly classified (i.e., classified as malignant) by the expert, compared to 95% (762/800) of the benign tumours, 96% (162/169) of the primary invasive tumours and 93% (39/42) of the metastatic tumours [45]. A logistic regression model has been constructed using data of the 54 "uncertain" cases with papillary projections in IOTA 1, but its performance was not stable: the area under the ROC curve was 0.88 on the development set, but dropped to 0.73 on a independent test set. Combining all data available in IOTA 1, 1b and 2 (IOTA 2 data collection just recently closed), we now have full preoperative information of 3,500 tumours including 250 cases which were difficult to peoperatively predict according to the investigators. Moreover, tumours for which the output of the main logistic regression is close to the decision boundary can also be regarded as difficult to classifiy. Using all available grey scale ultrasound and colour Doppler data of difficult tumours in IOTA 1, 1b and 2, a logistic regresison model has specifically been developed for these difficult masses, with an area under the ROC curve of 0.85 on both development and test set (unpublished data).
In IOTA Phase 3, the investigators will have the preoperative prediction based on this new logistic regression model. Besides this, they can also apply ultrasound contrast (O2), proteomics (O3), new tumour markers (O4) or 3D power Doppler (O5)) depending on their preference. This will make it possible to define an optimal algorithm to preoperatively identify maligancy among the small but yet important proportion of ovarian masses for which until now malignancy could not be ruled out, not even by a highly experienced ultrasonographer.

4. Detailed research description & Work Plan

Introduction: IOTA Phase 3

The work Plan is entirely focused to IOTA Phase 3 data collection and validation of the complete diagnostic algorithm. The aim of IOTA Phase 3 is to incorporate the IOTA mathematical models that predict the character of an adnexal mass in daily clinical practice using centre-specific cut-offs and to evaluate second stage tests in patients with adnexal masses that are difficult to classify in order to obtain more reliable results. For clarity of exposition we repeat here the objectives.

The objective of IOTA Phase 3

The main objective of this project is to improve preoperative diagnosis and subsequent management of patients with adnexal tumours by advanced algorithms in order to decrease morbidity and costs and in order to improve survival of patients with ovarian cancer. In particular, we aim to validate the added value of mathematical models as a new diagnostic tool in the prediction of ovarian cancer in clinical practice. First of all, we aim to prove their enhanced diagnostic performance and generalized applicability as a first stage examination. In cases where

prediction is unreliable, we aim to further improve the predictive performance of this diagnostic tool with second stage tests.
To achieve these objectives the project is divided in work packages each having its own sub-objective.

WP1: Prospective external validation of predictive mathematical models and pattern recognition to distinguish between malignant and benign adnexal masses
WP2: Validation of intravenous ultrasound contrast agents: quantitative analysis of contrast uptake and washout in tumor
WP3: Validation of proteomic analysis
WP4: Validation of a new set of tumour markers
WP5: Validation of 3D power Doppler
WP6: Validation of a new model, based on grey scale ultrasound and colour Doppler information, specifically designed for "difficult" tumours

The Work Plan now describes each of these Work Packages and details how each objective is achieved. The link between the different work packages is made clear in the schematic representation of the diagnostic algorithm. Realisation of the work plan should result in optimising all parameters of this diagnostic scheme with respect to diagnostic performance.

Work package 1:

Objective 1: **Prospective external validation of predictive mathematical models and pattern recognition to distinguish between malignant and benign adnexal masses**

The aim of this work package is to validate the IOTA mathematical models that predict the character of an adnexal mass in daily clinical practice using centre-specific cut-offs and to select those patients that are difficult to classify or to diagnose (i.e. with uncertain subjective assessment or uncertain predictive value) for a second stage examination. This work package is further subdivided in 6 tasks.

Task 1.1: Study protocol
The detailed study protocol for IOTA Phase 3 will be approved by the IOTA Steering Committee and then submitted to all relevant Ethical Committees for clinical studies.
Patients will give informed consent prior to participate in this study. Blood samples are only taken after obtaining written informed consent from each patient.

Task 1.2: Data collection and management
For the data collection of IOTA phase 1 and IOTA phase 2, a dedicated, secure data collection system was developed. A unique identifier was generated automatically for each patient's record based on the identifier for the centre, the patient's birthday and the date of the ultrasound scan. Clinicians at each centre could only view or update patients records from their own centre. In IOTA phase 1, the data collection was performed web-based, the investigators needed to login and to complete online a case report from. Data security was ensured by not recording the patient's name and by encrypting all data communication using a 54-bit SSL (Secure Socket Layer) certificate. For IOTA phase 2, an Astraia software application has been developed (www.astraia.com) which enables the researchers to complete the data offline in an electronical case report form. And afterwards, they transferred it to a central database located in Belgium. Since this second method was approved much more user-friendly by the clinicians, the data collection of IOTA phase 3 will be similarly executed.
Each participating centre inserts the clinical and ultrasound data in a specifically designed Astraia study screen. No missings are allowed during data submission, except for the CA-125 marker. A subjective assessment whether the tumour seems benign or malignant as well as the degree of certainty is given by the ultrasound examiner and this assessment is frozen in the local Astraia database before the results of the mathematical model become available to the examiner.

VARIABLES needed to test mathematical models in IOTA PHASE 3

Variable	Acronym	LR	BMLP	BPER	LS-SVM/ RVM	MC1	MC2
Personal history of ov. cancer	Pershistovca	X	X	X	X	X	X
Current use of hormonal therapy	Hormtherapy	X	X		X		
Age	Age	X	X	X	X	X	X
Diameters of the lesion	LesD1-3	X	X	X		X	X
Pain during examination	Pain	X	X				
Ascites	Ascites	X	X	X	X	X	X
Blood flow within papillary proj.	Papflow	X	X	X	X	X	X
Locularity	Locularity	X	X	X	X	X	X
Diameters of solid component	SolidD1-3	X	X	X	X	X	X
Irregular internal cyst walls	Wallregularity	X	X	X	X	X	X
Acoustic shadows	Shadows	X	X	X	X		X
Color Doppler score	Colscore	X	X	X	X		
Number of papillary projections	Papnr		X				X
Diameters of the ovary	OvD1-3				X		
Bilateral tumors	Bilateral					X	X
Suspected origin (ovary vs other)	Origin				X		

LR (logistic regression):
- LR1
- LR2
- objLR
- regLR

BMLP (Bayesian multi-layer perceptron):
- BMLP11-2a
- BMLP11-2b

BPER (Bayesian perceptron):
- BPER11

LS-SVM/RVM (Bayesian least squares support vector machines/relevance vector machines):
- BLSSVMlin (using linear kernel)
- BLSSVMrbf (using radial basis function kernel)
- BLSSVMaddrbf (using additive RBF kernel)
- RVMlin
- RVMrbf
- RVMaddrbf

MC1 and MC2 (Multi-class models):
- MLR (multi-category logistic regression)
- LR-PC (binary LR models combined using pairwise coupling)
- LR-PC2 (similar, but different inputs)
- BLSSVM-PC (binary Bayesian LS-SVMs + PC)
- KLR-PC (binary kernel logistic regression models + PC)
- MKLR (multi-class KLR)

The data are stored locally and by clicking “send data from FTP” all data are automatically and anonymously transferred to the central server in Leuven, where the datamanager checks them.

Task 1.3: Data quality checks and storage
In Leuven a full protocol has been developed for quality checks: 1) cross checks, e.g. in multilocular cysts you may not find papillary structures and 2) manual verification of the outliers. This protocol has already succesfully been applied to the data of IOTA phase 1 and 2.
Once the data has passed all quality checks, it is stored in the central database.

Task 1.4: Prospective external model validation
Once the data have been collected, all models of IOTA phase 1 and 2 (i.e. logistic regression models, scoring systems, neural networks, support and relevance vector machines) will be validated prospect-ively. The AUC, sensitivity, specificity, positive and negative predictive values will be computed.

Task 1.5: Patient selection for Second stage tests
The IOTA 2 dataset enables us to describe the population characteristics of each centre in correlation with the performance of the models in that centre. This gives us the opportunity to define centre-specific cut-offs above which an adnexal mass is classified as malignant. Whenever the probability of malignancy is amongst the 15% that lies close to the decision boundary of LR1, the examiner will be obliged to perform one of the second stage tests. In this way, we expect that the adnexal masses of approximately 300 patients will be classified as "difficult" and will need a second stage test.

Work package 2:

Objective 2: **Validation of intravenous ultrasound contrast agents: quantitative analysis of contrast uptake and washout in tumour**

The aim of this work package is to validate the preliminary study. Patients enrolled in task 2.1 will undergo a baseline (unenhanced) and contrast-enhanced examination. Correlations among results from B-mode, colour Doppler and CnTI-SonoVue examinations and the histopathologic diagnosis will be investigated. This work package is further subdivided in 2 tasks:

Task 2.1: Data collection
To validate the existing model on its discriminating power new patients will be enrolled. Each patient will undergo an unenhanced (baseline) and a contrast-enhanced ultrasound evaluation. Ultrasound examinations will be performed with a high-resolution (9.0- to 5.0-MHz) Technos MPX endovaginal probe (Esaote SpA, Genoa, Italy). The contrast-enhanced examination will be performed with the CnTI technology applied to the transvaginal probe (Esaote SpA, Genoa, Italy) and with SonoVue (Bracco International BV, Amsterdam, the Netherlands). The CnTI technology works at very low acoustic pressure (derated pressure =126 kPa) or a very low mechanical index (<0.1). Each patient will receive SonoVue in a bolus dose of 4.8 ml. The examination starts from the injection of the bolus of SonoVue for 3 minutes or until the end of the contrast effect.

Task 2.2: Validation of the model
The presence, the amount of vascularisation (colour score) and the pattern (regular or chaotic) of blood flow detected with colour Doppler and contrast-enhanced images will be investigated to assess whether the use of CnTI technology provides advantages with respect to the unenhanced ultrasound examination in the assessment of gynaecologic diseases, as seen in the preliminary results. Results will be validated using a case-control design. The above tumours for which intravenous contrast agents have been applied are considered as the "cases". In IOTA 1, 1b and 2 a "historical control group" of difficult masses will be composed with two matching factors: 1) the same tumour type and 2) age (<50 versus ≥50). The difference in sensitivity and specificity of the investigator's diagnosis between the cases with intravenous contrast and the control group will be assessed. The value of quantitative assessement of contrast ultrasound as second stage test in difficult tumours will also be compared with other second stage tests that were applied in the same patients.

Work package 3:

Objective 3: ***Validation of proteomic analysis***

The proteomics work package aims to develop a second stage test for distinguishing benign and malignant adnexal masses using liquid chromatography (LC) and MALDI-based mass spectrometry. It will continue seamlessly the work that is currently being done in our pilot study. The pilot study consists of a set of 39 serum samples, 20 benign samples and 19 malignant samples which are being analysed using MALDI-TOF based proteomic profiling. After proper processing of the data, mathematical and probabilistic methods will be used to develop models based on a panel of m/z peaks. The combination of m/z peaks with the highest diagnostic performance will constitute the PILOTMODEL. This workpackage aims to (1) expand the number of samples analysed and (2) validate the PILOTMODEL. This will be accomplished by making use of the state-of-the-art mass spectral analysis capabilities that have recently become available to us at the Katholieke Universiteit Leuven through the foundation of the Interfaculty Centre for Proteomics and Metabolomics (ProMeta).
The number of samples will be increased by 100 and this new set of patients will be called the prospective data set. The additional proteomics experiments will be carried out on set of patients which reflect the distribution of benign and malignant samples. Then, after proper processing of the data, this set of patients will be used to assess the predictive performance of the PILOTMODEL. If unsatisfactory this set of patients can be used to further improve and refine the PILOTMODEL. This entails the identification and verification of the proteins and/or peptides that are part of the diagnostic model with the best discriminatory performance. The identification allows to replace the m/z values by their corresponding biomolecules and allows to decouple the model from mass spectrometry.

Hereby we will make use of mathematical and probabilistic methods that model the data and predict clinically relevant classes. First, pre-processing techniques will be developed and used to allow comparisons between spectra. Next, both univariate and multivariate techniques will be used to develop models that are able to distinguish benign from malignant neoplasms. This will be accomplished by using both standard and advanced statistical and mathematical data analysis techniques (e.g. logistic regression, LS-SVMs, Bayesian networks). This work package is further subdivided in 3 tasks:

Task 3.1: Sample collection for proteomics second stage test
The aim is to test the clinical application of proteomics for the pre-operative classification of ovarian tumours. The following protocol is being used to collect samples where approximately 100 patients with an adnexal mass will be recruited and studied within 35 days before investigative surgery. Medical and family histories will be recorded. A sample of peripheral venous blood will be taken for the proteomic analysis. Findings at surgery and the histological classification of excised tissues as malignant or benign (and by cell type) will be used as outcome measures. Informed consent must be signed before collection of blood sample. Standard blood clotting tubes without any anti-coagulantia are used for the collection of serum samples. Venous blood is collected in an 8-10 ml clotting tube, this will yield ± 3-4 ml serum. Samples are collected by adequately trained medical personnel only (nurse or doctor). Once the blood is taken, the tube is mixed gently end over end. The tubes are placed in a plastic bag (per donor) and placed at 4°C before and during transport to the laboratory. The maximum allowed time between collection and preparation of the serum sample is 3 hours.

Task 3.2: LC-MALDI Mass spectrometry
Mass spectrometric measurements are carried out in the gas phase on ionized analytes. By definition, a mass spectrometer consists of an ion source, a mass analyser that measures the mass-to-charge ratio (m/z) of the ionized analytes, and a detector that registers the number of ions at each m/z value (Aebersold and Mann, 2003). The ion source is known as Matrix Assisted Laser Desorption/Ionization (MALDI). Its function is to volatise and ionise the proteins and peptides for further analysis. It does this by using laser pulses to desorp and ionise the molecules under study out of a dry, crystalline chemical matrix. The mass analyser is based on a Time-Of-Flight tube design (TOF) and allows for single MS measurements to be performed. For identification of molecules two TOF analysers are used in a tandem MS setting which allows for the fragmentation of the proteins under study. For biomacromolecules such as proteins, fragmentation into smaller peptide fragments is a prerequisite for reliable identification.

Task 3.3: Pre-processing and mathematical modeling (*in silico* analysis)
The analysis of mass-spectrometry data is far from straightforward because of its high dimensional nature. It is virtually impossible to manually analyse results and search for interesting diagnostic proteins and/or peptides.

Therefore advanced methods that draw from statistics, machine learning, probability theory and mathematics are necessary to analyse the data that results from MALDI-TOF/TOF. Furthermore, the analysis is subdivided in two steps corresponding to two objectives in this work package: pre-processing and model development.

Pre-processing: The goal of pre-processing is to account for time and mass disturbances between spectra of different samples. Peaks in the mass spectrum correspond to individual proteins or peptides (or fractions thereof) and their peak heights are related to their concentration. When analysing mass spectrometry data for biomarker discovery only a subset of peaks that result from ionisation of biomolecules such as peptides or proteins are biologically significant and of use in applications. In order to detect and locate these peaks, the raw data is subjected to several pre-processing steps: base line correction, smoothing, peak detection and peak alignment. Base line correction and smoothing can be handled using filtering methods and curve fitting techniques. Peak detection and alignment will be handled using wavelet analysis. Wavelets have been widely used to de-noise signals in several number of contexts (e.g. magnetic resonance, ultrasound blood flow and computed tomography) and can be used to extract desirable features from the data. This results in the location of peaks and their quantification by combining elements from signal processing with wavelet analysis.

Model development: The pilot data set will be used to develop a model (i.e. PILOTMODEL) which will subsequently be validated using the prospectively collected data form this project. Both univariate and multivariate data analysis will be used for model development on the pre-processed data. Univariate data analysis will be used to look for single diagnostic peaks. For this purpose, non-parametric statistics will be used (i.e. Wilcoxon rank sum tests). Moreover the predictive performance of each single peak will be estimated using sensitivity, specificity, Area Under the ROC curve (AUC) and likelihood ratios. We expect that a single biomarker will be unsatisfactory to accurately discriminate benign and malignant ovarian cancer. Therefore multivariate analysis will be performed. This will allow to develop a panel of peaks which may have poor predictive performance in a univariate approach but reach significance when combined with other peaks. For this purpose we will use both standard and advanced mathematical techniques. Currently we plan to use stepwise logistic regression, Least-Squares Support Vector Machines and Bayesian network analysis. These three methods have already proven their usefulness in medical and biological data analysis and offer a complementary view on the data.

Work package 4:

Objective 4: ***Validation of new set of tumour markers***

External validaton is needed to test the added value of combinations of novel tumour markers to the standard clinical and ultrasound variables in patients with adnexal tumours. Therefore, two tasks have to be executed:

Task 4.1: Data collection

In tumours needing a 2nd stage test in order to make a reliable preoperative diagnosis, a blood sample is taken and later on a combination of the following markers will be assessed:

1. CA 125 II
2. CA 15-3
3. CA 72-4
4. CEA
5. macrophage colony-stimulating factor (M-CSF)
6. HE4
7. Mesothelin
8. Osteopontin
9. kallikrein(s)

However, if in the meantime more promising markers are proposed, some of these markers may be replaced by others.

Task 4.2: Validation of the model

Similarly as to the validation of 2nd stage tests in WP2, each new tumour marker will be assessed using a case-control study design with tumours where a new marker was present considered as “cases” and difficult masses present in the data of IOTA 1, 1b and 2 that match the “cases” in type of tumour and patient’s age (<50 versus ≥50) considered as “controls”. Each new tumour marker will be validated in terms of possible increased sensitivity or specificity.

Work package 5:

Objective 5: **Validation of 3D power Doppler**

The aim of this study is to evaluate the potential benefit of 3D power Doppler as a second stage examination in order to be able to classify the difficult masses more correctly as benign or malignant.

Task 5.1: Data collection
In centres where a 3D ultrasound equipment is available, 3D power Doppler volumes of the adnexal mass will be collected. The vascularisation index will be calculated and the vascular tree described. This will be performed in the centre where the volumes are collected. Afterwards a copy of all of the stored 3D volumes will be sent to Prof. Lil Valentin (University Hospital, Malmö, Sweden) where she will independently recalculate all of the vascularisation indices. In this way, we will be able to correct for interobserver variability.

Task 5.2: Validation of the model
A case-control study will be performed to validate the use of 3D ultrasound as a second stage test. Masses in IOTA Phase 3 which have been investigated with 3D ultrasound are denoted as the “cases”. A historical control group is constructed using data of difficult masses available in IOTA Phase 1, 1b and 2. Two matching factors will be applied: 1) same tumour type and 2) age (<50 versus ≥50). This will enable us to assess whether 3D ultrasound results in a higher detection rate of malignant masses (higher sensitivity) or a decrease in the number of unnecessary exploratory laparotomies (higher specificity).

Work package 6:

Objective 6: **Validation of a new model, based on grey scale ultrasound and colour Doppler information, specifically designed for difficult tumours.**

The aim is to validate a logistic regression model, based on grey scale ultrasound and colour Doppler, specifically designed for difficult tumours. The main question is: “Can unnecessary exploratory laporotomies be avoided in a number of cases?” Study participants in the prospective set will be offered in a randomised way the standard hospital approaches or standard hospital approaches + the model result. This work package is further subdivided in 2 tasks:

Task 6.1: Data collection
At least 2,000 new patients with persisting adnexal tumours will be examined. This results in a collection of roughly 300 difficult masses. The hypothesis is that adding the prediction by the model, developed for this specific subgroup of tumours, to the decision tree allows a significant reduction in the number of exploratory laparotomies for benign tumours when compared to the standard hospital procedure. Therefore, cases where the clinicians state to be uncertain about the diagnosis, will be randomised in two arms in 1:2 ratios:

- arm (A): standard hospital procedure approach
- arm (B): standard hospital procedure approach + result of difficult tumours model.

So, we will have roughly 100 cases in the control arm (arm A) and 200 cases in the model arm (arm B).

Task 6.2: Validation of the model

In both arms, the sensitivity and specificity will be calculated and compared. An increase of 10% specificity in the model arm (arm B), at an equivalent sensitivity in arm A and B, will be regarded as clinically relevant.

This analysis will be performed twice: 1) overall and 2) stratified by the degree of expertise of the ultrasound examiner. The 2nd analysis is planned due to the fact that less experienced ultrasound examiners might benefit more from the use of such model for difficult tumours than highly experienced investigators.

Expertise in supporting centres

Division SCD, Department of Electrical Engineering (ESAT) of the Katholieke Universiteit Leuven.

SCD's major research objective is to design and build advanced methods for crucial problems in information processing. It builds on the enormous growth in computer power, communication bandwidth and available data and the various needs in society for effective use of these opportunities. The strength of the group is the use of mathematical engineering/engineering mathematics from mathematical fields such as linear and multi-linear algebra, statistics, discrete mathematics, differential geometry, and optimization. In this way, SCD has built up world recognised expertise in bioinformatics, biomedical data processing, signal (audio, communications) processing, cryptography, embedded systems, data mining, neural networks, identification, control, Two research groups with complementary expertise within the Division of SCD, are participating in the project, i.e.

1. The **SCD/BIOMED** team consists of 1 staff member, 3 postdocs, 15 PhD students. Research – fundamental/theoretical as well as application oriented- is performed in the domain of (multi)linear algebra, (non)linear signal analysis, classification and system identification with special focus to the development of numerically reliable and robust algorithms for improving medical diagnostics. In this domain the group has built up an international reputation (more than 150 publications, look http://www.esat.kuleuven.ac.be/sista/). Applications under study are: quantification of metabolite concentrations using in-vivo Magnetic Resonance Spectroscopic (MRS) data and images, quantification of brain oxygenation in neonates using (functional) Near-Infrared Spectroscopy, quantification of cardiovascular dynamics and auto-regulation, heart-rate variability, detection of somato-sensory evoked potentials in EEG, preoperative classification of (brain, ovarian, prostate) tumours and prediction/detection of epileptic seizures based on scalp-EEG monitoring. At present, research focuses on the integrated multimodal and multichannel data processing, analysis and decision support for simultaneously acquired biomedical data such as EEG, ECG, EMG, (functional) MRI, ultrasound, PET and SPECT.
2. The **SCD/BIOI** group is one of the largest bioinformatics groups in Belgium with extensive internationally renowned experience in the integration of mathematical, statistical, and computational methods to analyze biological, biochemical, and biophysical data. The group is specialised in bioinformatics and more particularly in the analysis of large, complex data sets to identify biological and/or clinical relationships between different parameters. A high level of expertise in bioinformatics is a key element to successfully accomplish the goals of the current project proposal. Within the ESAT-SCD group, expertise in all necessary domains is present as well as the necessary hardware and software background. Our research focus is on gene prioritisation, gene network inference, motif detection and disease management, see www.kuleuven.be/bioinformatics for more detailed information. SCD/Bioi operates internationally at the cutting edge of its frontline enabling discipline. The scope of its activities are regional, national and international.

Regarding **proteomics** the research in this project is performed in the Interfaculty Centre for Proteomics and Metabolomics (ProMeta) at the KULeuven. ProMeta has cutting-edge wet-lab and in silico technologies.

UZ Leuven

The Department of Women & Child has a large experience in gynaecologic ultrasound and ovarian cancer treatment. It is the largest referral centre for gynaecologic oncology in Flanders.

A prospective collection of the data on ovarian tumours started in 1994 in UZ Leuven. Since 1996 there is a close cooperation between D. Timmerman and I. Vergote of the University Hospitals of Leuven and the SCD division of ESAT which has resulted in many publications and projects. The Department also initiated the IOTA consortium. The IOTA study is a multicentric cooperation with renowned universities in Lund/Malmö, Leuven, Rome, London, Milan, Monza, Napoli, Lublin, Cagliari, Beijing, Udine and Prague. This international collaboration is technically supported by ESAT-SCD, K.U. Leuven, with software and internet-applications.

Phase 1 of the IOTA study started in 1999 and was concluded in 2002. In this phase data from 1,066 patients with an adnexal tumour were used to construct a large database with potentially important medical parameters. Several new mathematical models were developed. Phase 2 started in October 2005 till October 2007 and further expanded this database with 1,938 patient data from 19 centres.

References

1. Vergote I, De Brabanter J, Fyles A, Bertelsen K, Einhorn N, Sevelda P. Prognostic importance of degree of differentation and cyst rupture in stage I invasive epithelial ovarian carcinoma. Lancet 2001; 357: 176-182
2. Medeiros LR, Fachel JM, Garry R, Stein AT, Furness S. Laparoscopy versus laparotomy for benign ovarian tumours. Cochrane Database Syst Rev. 2005; 20: CD004751
3. Carley ME, Klingele CJ, Gebhart JB, Webb MJ, Wilson TO. Laparoscopy versus laparotomy in the management of benign unilateral adnexal masses. J Am Assoc Gynecol Laparosc 2002; 9: 321-6
4. Hacker NF, Berek JS, Lagasse LD, Primary cytoreductive surgery for epithelial ovarian cancer. Obstet Gynecol 1983; 61:431-420
5. Valentin L. Prospective cross-validation of Doppler ultrasound examination and gray-scale ultrasound imaging for discrimination of benign and malignant pelvic masses. Ultrasound Obstet Gynecol 1999; 14: 273-83
6. Ferrazzi E, Zanetta G, Dordoni D, Berlanda N, Mezzopane R, Lissoni G. Transvaginal ultrasonographic characterization of ovarian masses : a comparison of five scoring systems in a multicenter study. Ultrasound Obstet Gynecol 1997; 10: 192-197
7. Aslam N, Banerjee S, Carr J, Savvas M, Hooper R, Jurkovic D. Prospective evaluation of logistic regression models for the diagnosis of ovarian cancer. Obstet Gynecol 2000; 96: 75-80
8. Mol BW, Boll D, De Kanter M, Heintz P, Sijmons E, Oei G. Distinguishing the benign and malignant adnexal mass: an external validation of prognostic models. Gyn Oncol 2001; 80: 162-167
9. Valentin L. Comparison of Lerner score, Doppler ultrasound examination, and their combination for discrimination between benign and adnexal masses. Ultrasound Obstet Gynecol 2000; 15: 143-147
10. Van Holsbeke C, Van Calster B, Valentin L, Testa AC, Ferrazzi E, Dimou I, Lu C, Moerman P, Van Huffel S, Vergote I, Timmerman D. External validation of mathematical models to distinguish between benign and malignant adnexal tumors: a multicenter study by the international ovarian tumor analysis group. Clin Cancer Res 2007; 13: 4440-7
11. Tekay A, Jouppila P. Validity of pulsatility and resistance indices in classification of adnexal tumors with transvaginal color Doppler ultrasound. Ultrasound Obstet Gynecol 1992; 2:338–344.
12. Timmerman D, Valentin L, Bourne TH, Collins WP, Verrelst H, Vergote I. Terms, definitions and measurements to describe the sonographic features of adnexal tumors: a consensus opinion from the International Ovarian Tumor Analysis (IOTA) Group. Ultrasound Obstet Gynecol 2000;16:500-5. Review.
13. Marret H, Ecochard R, Giraudeau B, Golfier F, Raudrant D, Lansac J. Color Doppler energy prediction of malignancy in adnexal masses using logistic regression models. Ultrasound Obstet Gynecol. 2002; 20: 597-604.
14. Marret H, Sauget S, Giraudeau B, Body G, Tranquart F. Power Doppler vascularity index for predicting malignancy of adnexal masses. Ultrasound Obstet Gynecol. 2005; 25: 508-13
15. Orden M.R., Jurvelin J.S. and Kirkinen P.P.. Kinetics of a US contrast agent in benign and malignant adnexal tumors, Radiology 226 (2003), pp. 405–410.
16. Petricoin EF *et al.* Use of proteomic patterns in serum to identify ovarian cancer. The Lancet 2002, vol 359, 572-577
17. Check E. Proteomics and cancer: Running before we can walk? 2004 Nature 429, 496-497
18. Diamandis EP. OvaCheck: doubts voiced soon after publication 2004 Nature 430, 611
19. Diamandis EP. Proteomic patterns in serum and identification of ovarian cancer Lancet. 2002 Jul 13;360(9327):170
20. Baggerly K, Morris J, Coombes K. Reproducibility of SELDI-TOF protein patterns in serum: comparing datasets from different experiments. Bioinformatics 2004 20(5),777-785
21. Sorace J, Zhan M. A data review and re-assessment of ovarian cancer serum proteomic profiling. BMC Bioinformatics 2003,4:24
22. Rockhill B. Proteomic patterns in serum and identification of ovarian cancer. Lancet. 2002 Jul 13;360(9327):169
23. Elwood M. Proteomic patterns in serum and identification of ovarian cancer. Lancet. 2002 Jul 13;360(9327):170
24. Pearl DC, Proteomic patterns in serum and identification of ovarian cancer. Lancet. 2002 Jul 13;360(9327):169-70
25. Villanueva J, Tempst P. OvaCheck: let's not dismiss the concept. 2004 Nature 430, 611
26. Bast RC Jr, Badgwell D, Lu Z, Marquez R, Rosen D, Liu J, Baggerly KA, Atkinson EN, Skates S, Zhang Z, Lokshin A, Menon U, Jacobs I, Lu K. New tumor markers: CA125 and beyond. Int J Gynecol Cancer. 2005 Nov-Dec;15 Suppl 3:274-81.
27. Timmerman D, Schwärzler P, Collins WP, Claerhout F, Coenen M, Amant F. Subjective assessment of adnexal masses with the use of ultrasonography: an analysis of interobserver variability and experience. Ultrasound Obstet Gynecol 1999; 13: 11-16
28. Valentin L. Pattern recognition of pelvic masses by gray-scale ultrasound imaging: the contribution of Doppler ultrasound. Ultrasound Obstet Gynecol 1999; 14: 338-347
29. Jacobs I, Oram D, Fairbanks J, Turner J, Frost C, Grudzinskas J. A risk of malignancy index incorporating CA 125, ultrasound and menopausal status for the accurate preoperative diagnosis of ovarian cancer. BJOG 1990; 97: 922-929
30. Lu C., Suykens J.A.K., Timmerman D., Vergote I., Van Huffel S. Linear and nonlinear preoperative classification of ovarian tumors. Chapter 11 of Knowledge Based Intelligent System for Health Care, (Ichimura T. and Yoshida K. , ed.), vol. 7 of International Series on Advanced Intelligence, Advanced Knowledge International (Magill, Australia), 2004, 343-382
31. Timmerman D, Bourne T, Tailor A, Collins WP, Verrelst H, Vandenberghe K. A comparison of methods for preoperative discrimination between malignant and benign adnexal masses: The development of a new logistic regression model. Am J Obstet Gynecol 1999; 181: 57-65
32. Timmerman D, Verrelst H, Bourne TH, De Moor B, Collins WP, Vergote I. Artificial neural network models for the preoperative discrimination between malignant and benign adnexal masses. Ultrasound Obstet Gynecol 1999; 13: 17-25

33. Tailor A, Jurkovic D, Bourne T, Collins WP, Campbell S. Sonographic prediction of malignancy in adnexal masses using multivariate logistic regression analysis. Ultrasound Obstet Gynecol 1997; 10: 41-47
34. Valentin L, Sladkevicius P, Marsál K. Limited contribution of Doppler velocimetry to the differential diagnosis of extrauterine pelvic tumors. Obstet Gynecol 1994; 83: 425-433
35. Tekay A, Jouppila P. Controversies in assessment of ovarian tumors with transvaginal color Doppler ultrasound. Acta Obstet Gynecol Scand 1996; 75: 316-329
36. Valentin L. Pattern recognition of pelvic masses by gray-scale ultrasound imaging: the contribution of Doppler ultrasound. UOG 1999; 14: 338-347
37. Mol BW, Boll D, De Kanter M, Heintz P, Sijmons E, Oei G. Distinguishing the benign and malignant adnexal mass: an external validation of prognostic models. Gyn Oncol 2001; 80: 162-167
38. Sladkevicius P, Jokubkiene L, Valentin L. Contribution of morphological assessment of the vessel tree by three-dimensional ultrasound to a correct diagnosis of malignancy in ovarian masses. UOG 2007; 30: 874-82
39. Oral communication on the 15th ISUOG world congress in Vancouver. Sladkevicius P, Jokubkiene L, Valentin L. Assessment of the vascular trees in ovarian tumors using 3D power Doppler ultrasound
40. Cohen L, Escobar P, Scharm C, Glimco B, Fishman D. Three-dimensional power Doppler ultrasound improves the diagnostic accuracy for ovarian cancer prediction. Gynecol Oncol 2002; 84: 352-3
41. Kurjak A, Kupesic S, Sparac V, Kosuta D. Three-dimensional ultrasonographic and power Doppler characterization of ovarian lesions. Ultrasound Obstet Gynecol 2000; 16: 365-71
42. Jokubkiene L, Sladkevicius P, Valentin L. Does three-dimensional power Doppler ultrasound help in discrimination between benign and malignant ovarian masses? Ultrasound Obstet Gynecol 2007; 29: 215-225
43. Guerriero S, Ajossa S, Piras S, Gerada M, Floris S, Garau N, Minerba L, Paoletti AM, Melis GB. Three-dimensional quantification of tumor vascularity as a tertiary test after B-mode and power Doppler evaluation for detection of ovarian cancer. J Ultrasound Med 2007; 26: 1271-8
44. Timmerman D, Testa AC, Bourne T, Ferrazzi E, Ameye L, Konstantinovic ML., Van Calster B., Collins W.P., Vergote I., Van Huffel S., Valentin L., Logistic regression model to distinguish between the benign and malignant adnexal mass before surgery: a multicenter study by the International Ovarian Tumor Analysis Group. J Clin Oncol 2005; 23: 8794-801
45. Valentin L, Ameye L, Jurkovic D, Metzger F, Lécuru F, Van Huffel S, Timmerman D. Which extrauterine pelvic masses are difficult to correctly classify as benign or malignant on the basis of ultrasound findings and is there a way of making a correct diagnosis? UOG 2006; 27: 438-444
46. Van Calster B, Timmerman D, Nabney I, Valentin L, Testa AC5, Van Holsbeke C, Vergote I, Van Huffel S. Using Bayesian Neural Networks with ARD input selection to detect malignant adnexal masses prior to surgery 2007; Neural computing and applications. Accepted for publication.
47. Van Calster B, Timmerman D, Lu C, Suykens J, Valentin L, Van Holsbeke C, Amant F, Vergote I, Van Huffel S. Preoperative diagnosis of ovarian tumors using Bayesian kernel-based methods. UOG 2007; 29: 496-504
48. Testa A, Ferrandina G, Fruscella E, Van Holsbeke C, Ferrazzi E, Leone F, Arduini D, Exacoustos C, Bokor D, Scambia G, Timmermean D. The use of contrasted transvaginal sonography in the diagnosis of gynecologic diseases. J Ultrasound Med 24 (2005), pp. 1267-1278.
49. Testa A.C., Timmerman D., Exacoustos C., Fruscella E., Van Holsbeke C., Bokor D., Arduini D., Scambia G.and Ferrandina G. The role of CnTI-SonoVue in the diagnosis of ovarian masses with papillary projections: a preliminary study. *Ultrasound Obstet Gynecol*, 26: 644-650, 2005.
50. Timmerman D., Van Calster B., Jurkovic D., Valentin L., Testa A.C., Bernard J.P. et al. The inclusion of CA-125 does not improve mathematical models developed to distinguish between benign and malignant adnexal tumors. J Clin Oncol 2007, 25, 4194-4200
51. Van Calster B., Timmerman D., Bourne T., Testa A., Van Holsbeke C., Domali E., Jurkovic D., Neven P., Van Huffel S., Valentin L. Discrimination Between Benign and Malignant Adnexal Masses by Specialist Ultrasound Examination versus Serum CA-125. J Natl Cancer Inst 2007, 99, 1706-1714
52. Van Gorp T., Gevaert O., Van de Plas R., Waelkens E., De Moor B., Timmerman D., Vergote I. Biomarker discovery workflow with MALDI-TOF to distinguish between benign and malignant adnexal masses. Abstracts ESGO meeting, Berlin, 2007.
