# Supplementary material for: Vessel morphology depicted by three‐dimensional power Doppler ultrasound as second‐stage test in adnexal tumors that are difficult to classify: prospective diagnostic accuracy study
Source: Ultrasound Obstet Gynecol. 2021 Feb 1;57(2):324–34. doi: 10.1002/uog.22191 (PMC7898332; doi:10.1002/uog.22191)
Supplement: Supplementary file 2 — Appendix S2 Power Doppler and three‐dimensional (3D) ultrasound settings used in study [file UOG-57-324-s002.pdf]

Supplementary material

Appendix 2.

The power Doppler and three-dimensional (3D) ultrasound settings used.

Settings recommended when acquiring 3D power Doppler volumes using a Voluson 730 expert with a vaginal transducer 5 – 9 MHz

Power 100%

Gain 0.8

Frequency mid

Quality normal

Wall motion filter (WMF) low 1

Pulse repetition Frequency (PRF) 0.6KHz

Submenu

Smooth 5/6

Ensemble 16

Flow res set high

Line dens 7

PD map 5

Balance G > 170

Artefact on

L filter 3

It is important to get a good image of the vascular tree, and in some patients other settings might yield better results. It is important that the settings that detect most small vessels without artefacts (most sensitive settings possible) are used.

Settings recommended when acquiring 3D power Doppler volumes using a GE E8 version 7.03 or 7.05, with a vaginal transducer 5 – 9 MHz

Power 100%

Gain –0.0

Frequency mid

Quality high

Wall motion filter (WMF) mid 1

Pulse repetition Frequency (PRF) 0.6 KHz

Submenu

- Smooth rise 7
- Smooth fall 7
- PD map 5
- Flow res high
- Line dens 7
- Ensemble 21
- L filter 2
- Artefact on
- Balance 205

It is important to get a good image of the vascular tree, and in some patients other settings might yield better results. It is important that the settings that detect most small vessels without artefacts (most sensitive settings possible) are used.

Settings recommended when acquiring 3D power Doppler volumes using a GE E8 version 7.03 or 7.05, with a vaginal transducer 6 – 12 MHz

Power 100%

Gain –8.0

Frequency low

Quality normal

Wall motion filter (WMF) mid 1

Pulse repetition Frequency (PRF) 0.6 KHz

Submenu

- Smooth rise 7
- Smooth fall 7
- PD map 5
- Flow res mid 2
- Line dens 7
- Ensemble 13
- L filter 2
- Artefact on
- Balance 205

It is important to get a good image of the vascular tree, and in some patients other settings might yield better results. It is important that the settings that detect most small vessels without artefacts (most sensitive settings possible) are used.
