# Supplementary material for: Vessel morphology depicted by three‐dimensional power Doppler ultrasound as second‐stage test in adnexal tumors that are difficult to classify: prospective diagnostic accuracy study
Source: Ultrasound Obstet Gynecol. 2021 Feb 1;57(2):324–34. doi: 10.1002/uog.22191 (PMC7898332; doi:10.1002/uog.22191)
Supplement: Supplementary file 4 — Tables S1 and S2 Histological diagnoses of 2403 adnexal tumors, according to: whether tumor was difficult to classify and availability of ultrasound volumes (Table S1) and whether tumor was difficult to classify as benign or malignant (Table S2) [file UOG-57-324-s004.docx]

**Table S1** Histological diagnoses of 2403 adnexal tumors, according to whether tumor was difficult to classify on each assessment and availability of ultrasound volumes

|  |  | | **ALL** | | | | | | | | | | **3D volume analyzed** | | | | | | | |
| --- | --- | --- | --- | --- | --- | --- | --- | --- | --- | --- | --- | --- | --- | --- | --- | --- | --- | --- | --- | --- |
|  | **Total** | | Both US examiner and LR1 not uncertain | | Either US examiner or LR1 uncertain | | US examiner uncertain | | LR1 uncertain | | Both uncertain | | Either US examiner or LR1 uncertain | | US examiner uncertain | | LR1 uncertain | | Both uncertain | |
|  | N=2403 | | N=2027 | | N=376 | | N=168 | | N=259 | | N=51 | | N=138 | | N=79 | | N=87 | | N=28 | |
| **Benign** | **1423** | **(59%)** | **1169** | **(58%)** | **254** | **(68%)** | **111** | **(66%)** | **180** | **(70%)** | **37** | **(73%)** | **100** | **(72%)** | **52** | **(66%)** | **70** | **(80%)** | **22** | **(79%)** |
| Endometrioma | 344 | (14%) | 324 | (16%) | 20 | (5%) | 5 | (3%) | 16 | (6%) | 1 | (2%) | 7 | (5%) | 2 | (3%) | 5 | (6%) | - | - |
| Teratoma | 231 | (10%) | 212 | (10%) | 19 | (5%) | 5 | (3%) | 15 | (6%) | 1 | (2%) | 5 | (4%) | 1 | (1%) | 4 | (5%) | - | - |
| Simple cyst + parasalpingeal cyst | 106 | (4%) | 96 | (5%) | 10 | (3%) | 5 | (3%) | 7 | (3%) | 2 | (4%) | 3 | (2%) | 1 | (1%) | 3 | (3%) | 1 | (4%) |
| Functional cyst | 40 | (2%) | 29 | (1%) | 11 | (4%) | 6 | (4%) | 7 | (3%) | 2 | (4%) | 3 | (2%) | 2 | (3%) | 2 | (2%) | 1 | (4%) |
| Hydrosalpinx + salpingitis | 47 | (2%) | 40 | (2%) | 7 | (2%) | 3 | (2%) | 5 | (2%) | 1 | (2%) | 3 | (2%) | 1 | (1%) | 3 | (3%) | 1 | (4%) |
| Peritoneal pseudocyst | 18 | (<1%) | 14 | (<1%) | 4 | (1%) | 2 | (1%) | 3 | (1%) | 1 | (2%) | 2 | (1%) | 1 | (1%) | 2 | (2%) | 1 | (4%) |
| Abscess | 17 | (<1%) | 14 | (<1%) | 3 | (<1%) | 1 | (<1%) | 2 | (<1%) | - | - | 2 | (1%) | 1 | (1%) | 1 | (1%) | - | - |
| Fibroma | 130 | (5%) | 79 | (4%) | 51 | (14%) | 22 | (13%) | 33 | (13%) | 4 | (8%) | 18 | (13%) | 9 | (11%) | 10 | (11%) | 1 | (4%) |
| Serous cystadenoma | 259 | (11%) | 190 | (9%) | 69 | (18%) | 35 | (21%) | 50 | (19%) | 16 | (31%) | 30 | (22%) | 21 | (27%) | 21 | (24%) | 12 | (43%) |
| Mucinous cystadenoma | 183 | (8%) | 134 | (7%) | 49 | (13%) | 21 | (13%) | 32 | (12%) | 4 | (8%) | 23 | (17%) | 10 | (13%) | 16 | (18%) | 3 | (11%) |
| Rare benign | 48 | (2%) | 37 | (2%) | 11 | (3%) | 6 | (4%) | 10 | (4%) | 5 | (10%) | 4 | (3%) | 3 | (4%) | 3 | (3%) | 2 | (7%) |
|  |  |  |  |  |  |  |  |  |  |  |  |  |  |  |  |  |  |  |  |  |
| **Borderline** | **153** | **(6%)** | **103** | **(5%)** | **50** | **(13%)** | **24** | **(14%)** | **31** | **(12%)** | **5** | **(10%)** | **15** | **(11%)** | **7** | **(9%)** | **9** | **(10%)** | **1** | **(4%)** |
| Stage I | 135 | (6%) | 86 | (4%) | 49 | (13%) | 24 | (14%) | 30 | (12%) | 5 | (10%) | 14 | (10%) | 7 | (9%) | 8 | (9%) | 1 | (4%) |
| Stage II | 6 | (<1%) | 5 | (<1%) | 1 | (<1%) | - | - | 1 | (<1%) | - | - | 1 | (<1%) | - | - | 1 | (1%) | - | - |
| Stage III | 12 | (<1%) | 12 | (<1%) | - |  | - | - | - | - | - | - | - |  | - | - | - | - | - | - |
|  |  |  |  |  |  |  |  |  |  |  |  |  |  |  |  |  |  |  |  |  |
| **Primary invasive** | **701** | **(29%)** | **637** | **(31%)** | **64** | **(17%)** | **29** | **(17%)** | **44** | **(17%)** | **9** | **(18%)** | **22** | **(16%)** | **19** | **(24%)** | **8** | **(9%)** | **5** | **(18%)** |
| Stage I | 128 | (5%) | 103 | (5%) | 25 | (7%) | 12 | (7%) | 17 | (7%) | 4 | (8%) | 7 | (5%) | 6 | (8%) | 3 | (3%) | 2 | (7%) |
| Stage II | 47 | (2%) | 44 | (2%) | 3 | (<1%) | 1 | (<1%) | 2 | (<1%) | - | - | 1 | (<1%) | 1 | (1%) | - | - | - | - |
| Stage III | 397 | (17%) | 378 | (19%) | 19 | (5%) | 8 | (5%) | 13 | (5%) | 2 | (4%) | 8 | (6%) | 6 | (8%) | 3 | (3%) | 1 | (7%) |
| Stage IV | 61 | (3%) | 60 | (3%) | 1 | (<1%) | - | - | 1 | (<1%) | - | - | - | - | - | - | - | - | - | - |
| Rare | 68 | (3%) | 52 | (3%) | 16 | (4%) | 8 | (5%) | 11 | (4%) | 3 | (6%) | 6 | (4%) | 6 | (8%) | 2 | (2%) | 2 | (4%) |
|  |  |  |  |  |  |  |  |  |  |  |  |  |  |  |  |  |  |  |  |  |
| **Metastatic** | **126** | **(5%)** | **118** | **(6%)** | **8** | **(2%)** | **4** | **(2%)** | **4** | **(2%)** | **-** | **-** | **1** | **(<1%)** | **1** | **(1%)** | **-** | **-** | **-** | **-** |

US, ultrasound; LR1, logistic regression model 1; 3D, three-dimensional

**Table S2** Histological diagnoses of 2403 adnexal tumors, according to whether tumor was difficult to classify as benign or malignant

|  | Both US examiner and LR1  not uncertain | | Either US examiner or LR1  uncertain | | P-value |
| --- | --- | --- | --- | --- | --- |
|  | N=2027 | | N=376 | |  |
| **Benign** | **1169** | **(58%)** | **254** | **(68%)** | **<0.001** |
| Endometrioma | 324 | (16%) | 20 | (5%) | <0.001 |
| Teratoma | 212 | (10%) | 19 | (5%) | 0.01 |
| Simple cyst + parasalpingeal cyst | 96 | (5%) | 10 | (3%) | 0.72 |
| Functional cyst | 29 | (1%) | 11 | (4%) | 0.54 |
| Hydrosalpinx + salpingitis | 40 | (2%) | 7 | (2%) | 1 |
| Peritoneal pseudocyst | 14 | (<1%) | 4 | (1%) | 1 |
| Abscess | 14 | (<1%) | 3 | (<1%) | 1 |
| Fibroma | 79 | (4%) | 51 | (14%) | <0.001 |
| Serous cystadenoma | 190 | (9%) | 69 | (18%) | <0.001 |
| Mucinous cystadenoma | 134 | (7%) | 49 | (13%) | <0.001 |
| Rare benign | 37 | (2%) | 11 | (3%) | 0.94 |
|  |  |  |  |  |  |
| **Borderline** | **103** | **(5%)** | **50** | **(13%)** | **<0.001** |
| Stage I | 86 | (4%) | 49 | (13%) | <0.001 |
| Stage II | 5 | (<1%) | 1 | (<1%) | 1 |
| Stage III | 12 | (<1%) | - |  | 0.99 |
|  |  |  |  |  |  |
| **Primary invasive** | **637** | **(31%)** | **64** | **(17%)** | **<0.001** |
| Stage I | 103 | (5%) | 25 | (7%) | 0.98 |
| Stage II | 44 | (2%) | 3 | (<1%) | 0.82 |
| Stage III | 378 | (19%) | 19 | (5%) | <0.001 |
| Stage IV | 60 | (3%) | 1 | (<1%) | 0.008 |
| Rare | 52 | (3%) | 16 | (4%) | 0.77 |
|  |  |  |  |  |  |
| **Metastatic** | **118** | **(6%)** | **8** | **(2%)** | **0.008** |

P-values are corrected for multiple testing with the permutation method (Westfall PH, Wolfinger RD.

Multiple tests with discrete distributions. *Am Stat* 1997; **51**: 3-8)

US, ultrasound; LR1, logistic regression model 1
