# Supplementary material for: Vessel morphology depicted by three‐dimensional power Doppler ultrasound as second‐stage test in adnexal tumors that are difficult to classify: prospective diagnostic accuracy study
Source: Ultrasound Obstet Gynecol. 2021 Feb 1;57(2):324–34. doi: 10.1002/uog.22191 (PMC7898332; doi:10.1002/uog.22191)
Supplement: Supplementary file 5 — Tables S3 and S4 Clinical and ultrasound characteristics of 2403 adnexal tumors, according to: whether tumor was difficult to classify and availability of ultrasound volumes (Table S3) and whether tumor was difficult to classify as benign or malignant (Table S4) [file UOG-57-324-s005.docx]

**Table S3** Clinical and ultrasound characteristics of 2403 adnexal tumors, according to whether tumor was difficult to classify on each assessment and availability of ultrasound volumes

|  |  | | **ALL** | | | | | | | | | | **3D volume analyzed** | | | | | | | |
| --- | --- | --- | --- | --- | --- | --- | --- | --- | --- | --- | --- | --- | --- | --- | --- | --- | --- | --- | --- | --- |
|  | **Total** | | Both US examiner and LR1 not uncertain | | Either US examiner or LR1 uncertain | | US examiner uncertain | | LR1 uncertain | | Both uncertain | | Either US examiner or LR1 uncertain | | US examiner uncertain | | LR1 uncertain | | Both uncertain | |
|  | N=2403 | | N=2027 | | N=376 | | N=168 | | N=259 | | N=51 | | N=138 | | N=79 | | N=87 | | N=28 | |
| **Clinical variables** |  | |  | |  | |  | |  | |  | |  | |  | |  | |  | |
| Age, years | 50 ± 16 | | 49 ± 16 | | 53 ± 16 | | 52 ± 16 | | 53 ± 16 | | 49 ± 14 | | 54 ± 17 | | 54 ± 18 | | 51 ± 16 | | 49 ± 13 | |
| Postmenopausal | 1049 | (44%) | 861 | (42%) | 188 | (50%) | 79 | (47%) | 130 | (50%) | 21 | (41%) | 73 | (53%) | 41 | (52%) | 44 | (51%) | 12 | (43%) |
| Hysterectomy | 142 | (6%) | 115 | (6%) | 27 | (7%) | 15 | (9%) | 19 | (7%) | 7 | (14%) | 9 | (7%) | 6 | (8%) | 7 | (8%) | 4 | (14%) |
| Hormonal replacement therapy | 207 | (9%) | 174 | (9%) | 33 | (9%) | 26 | (15%) | 13 | (5%) | 6 | (12%) | 13 | (9%) | 12 | (15%) | 4 | (5%) | 3 | (11%) |
| Personal history ovarian cancer | 44 | (2%) | 36 | (2%) | 8 | (2%) | 2 | (1%) | 6 | (2%) | - | - | 4 | (3%) | 1 | (1%) | 3 | (3%) | - | - |
| Family history ovarian cancer | 74 | (3%) | 69 | (3%) | 5 | (1%) | 2 | (1%) | 3 | (1%) | - | - | 3 | (2%) | 1 | (1%) | 2 | (2%) | - | - |
| CA125, N available | 1451 | | 1198 | | 253 | | 114 | | 175 | | 36 | | 117 | | 67 | | 73 | | 23 | |
| CA125 | 42 (1 – 14067) | | 52 (1 – 14067) | | 23 (3 – 1948) | | 24 (6 – 906) | | 21 (3 – 1948) | | 18 (6 – 313) | | 20 (4 – 1302) | | 21 (7 – 906) | | 18 (4 – 1302) | | 17 (7 – 313) | |
| **Gray scale ultrasound variables** |  | |  | |  | |  | |  | |  | |  | |  | |  | |  | |
| Largest diameter, mm | 71 (10 – 550) | | 70 (10 – 550) | | 75 (10 – 322) | | 72 (10 – 322) | | 74 (10 – 300) | | 62 (10 – 169) | | 69 (10 – 310) | | 68 (10 – 310) | | 68 (10 – 300) | | 64 (10 – 122) | |
| Bilateral | 518 | (22%) | 465 | (23%) | 53 | (14%) | 16 | (10%) | 41 | (16%) | 4 | (8%) | 22 | (16%) | 9 | (11%) | 16 | (18%) | 3 | (11%) |
| Ascites | 340 | (14%) | 330 | (16%) | 10 | (3%) | 8 | (5%) | 2 | (<1%) | - | - | 5 | (4%) | 5 | (6%) | - | - | - | - |
| **Type of mass** |  |  |  |  |  |  |  |  |  |  |  |  |  |  |  |  |  |  |  |  |
| Unilocular | 600 | (25%) | 585 | (29%) | 15 | (4%) | 3 | (2%) | 12 | (5%) | - | - | 1 | (<1%) | - | - | 1 | (1%) | - | - |
| Unilocular solid | 258 | (11%) | 187 | (9%) | 71 | (19%) | 40 | (24%) | 46 | (18%) | 15 | (29%) | 23 | (17%) | 17 | (22%) | 14 | (16%) | 8 | (29%) |
| Multilocular | 413 | (17%) | 331 | (16%) | 82 | (22%) | 30 | (18%) | 60 | (23%) | 8 | (16%) | 32 | (23%) | 12 | (15%) | 23 | (26%) | 3 | (11%) |
| Multilocular solid | 505 | (21%) | 375 | (19%) | 130 | (35%) | 59 | (35%) | 89 | (34%) | 18 | (35%) | 56 | (41%) | 34 | (43%) | 36 | (41%) | 14 | (50%) |
| Solid | 627 | (26%) | 549 | (27%) | 78 | (21%) | 36 | (21%) | 52 | (20%) | 10 | (20%) | 26 | (19%) | 16 | (20%) | 13 | (15%) | 3 | (11%) |
| Nr locules | 1 (0 to >10) | | 1 (0 to >10) | | 2 (0 to >10) | | 2 (0 to >10) | | 2 (0 to >10) | | 2 (0 to >10) | | 4 (0 to >10) | | 2 (0 to >10) | | 3 (0 to >10) | | 2 (0 to >10) | |
| Pain at US examination | 344 | (14%) | 294 | (15%) | 50 | (13%) | 20 | (12%) | 32 | (12%) | 2 | (4%) | 9 | (7%) | 4 | (5%) | 5 | (6%) | - | - |
| **Echogenicity of cyst fluid** |  |  |  |  |  |  |  |  |  |  |  |  |  |  |  |  |  |  |  |  |
| Anechoic | 589 | (25%) | 485 | (24%) | 104 | (28%) | 39 | (23%) | 77 | (30%) | 12 | (24%) | 36 | (26%) | 17 | (22%) | 27 | (31%) | 8 | (29%) |
| Low level | 516 | (21%) | 392 | (19%) | 124 | (33%) | 63 | (38%) | 82 | (32%) | 21 | (41%) | 47 | (34%) | 32 | (41%) | 28 | (32%) | 13 | (46%) |
| Ground glass | 350 | (15%) | 326 | (16%) | 24 | (6%) | 9 | (5%) | 16 | (6%) | 1 | (2%) | 10 | (7%) | 4 | (5%) | 6 | (7%) | - | - |
| Hemorrhagic | 20 | (<1%) | 16 | (<1%) | 4 | (1%) | 2 | (1%) | 2 | (<1%) | - | - | 3 | (2%) | 2 | (3%) | 1 | (1%) | - | - |
| Mixed | 301 | (13%) | 259 | (13%) | 42 | (11%) | 19 | (11%) | 30 | (12%) | 7 | (14%) | 16 | (12%) | 8 | (10%) | 12 | (14%) | 4 | (14%) |
| No cyst fluid | 627 | (26%) | 549 | (27%) | 78 | (21%) | 36 | (21%) | 52 | (20%) | 10 | (20%) | 26 | (19%) | 16 | (20%) | 13 | (15%) | 3 | (11%) |
| Papillary projections present | 383 | (16%) | 265 | (13%) | 118 | (31%) | 69 | (41%) | 73 | (28%) | 24 | (47%) | 45 | (33%) | 35 | (44%) | 25 | (29%) | 15 | (54%) |
| Flow in papillation | 215 | (56%) | 169 | (64%) | 46 | (39%) | 32 | (46%) | 21 | (29%) | 7 | (29%) | 20 | (44%) | 16 | (46%) | 8 | (32%) | 4 | (27%) |
| Number of papillations | 2 (1 – ≥4) | | 2 (1 – ≥ 4) | | 2 (1 – ≥ 4) | | 2 (1 – ≥ 4) | | 1 (1 – ≥ 4) | | 2 (1 – ≥ 4) | | 2 (1 – ≥4) | | 2 (1 – ≥4) | | 1 (1 – ≥ 4) | | 2 (1 – ≥4) | |
| Height of papillation, mm | 10 (3 – 99) | | 11 (3 – 99) | | 7 (3 – 45) | | 8 (3 – 45) | | 6 (3 – 30) | | 7 (3 – 30) | | 7 (3 – 45) | | 7 (3 – 45) | | 5 (3 – 21) | | 6 (3 – 13) | |
| Mass with solid components | 1390 | (58%) | 1111 | (55%) | 279 | (74%) | 135 | (80%) | 187 | (72%) | 43 | (84%) | 105 | (76%) | 67 | (85%) | 63 | (72%) | 25 | (89%) |
| Largest diameter of largest solid component, mm | 50 (3 – 300) | | 54 (3 – 300) | | 25 (3 – 200) | | 28 (3 – 200) | | 22 (3 – 196) | | 19 (5 – 112) | | 24 (3 – 180) | | 25 (3 – 162) | | 16 (3 – 180) | | 13 (5 – 112) | |
| Incomplete septum | 113 | (5%) | 88 | (4%) | 25 | (7%) | 9 | (5%) | 17 | (7%) | 1 | (2%) | 2 | (1%) | 1 | (1%) | 1 | (1%) | - | - |
| Irregular walls | 957 | (40%) | 740 | (37%) | 217 | (58%) | 113 | (67%) | 141 | (54%) | 37 | (73%) | 77 | (56%) | 55 | (70%) | 44 | (51%) | 22 | (79%) |
| Shadows | 299 | (12%) | 239 | (12%) | 60 | (16%) | 20 | (12%) | 47 | (18%) | 7 | (14%) | 20 | (14%) | 10 | (13%) | 13 | (15%) | 3 | (11%) |
| **Doppler ultrasound variables** |  |  |  |  |  |  |  |  |  |  |  |  |  |  |  |  |  |  |  |  |
| Color Score |  |  |  |  |  |  |  |  |  |  |  |  |  |  |  |  |  |  |  |  |
| Score 1 | 606 | (25%) | 553 | (27%) | 53 | (14%) | 21 | (13%) | 36 | (14%) | 4 | (8%) | 10 | (7%) | 5 | (6%) | 7 | (8%) | 2 | (7%) |
| Score 2 | 762 | (32%) | 604 | (30%) | 158 | (42%) | 64 | (38%) | 115 | (44%) | 21 | (41%) | 44 | (32%) | 24 | (30%) | 31 | (36%) | 11 | (39%) |
| Score 3 | 681 | (28%) | 539 | (27%) | 142 | (38%) | 71 | (42%) | 94 | (36%) | 23 | (45%) | 70 | (51%) | 40 | (51%) | 42 | (48%) | 12 | (43%) |
| Score 4 | 354 | (15%) | 331 | (16%) | 23 | (6%) | 12 | (7%) | 14 | (5%) | 3 | (6%) | 14 | (10%) | 10 | (13%) | 7 | (8%) | 3 | (11%) |

US, ultrasound; LR1, logistic regression model 1 ; 3D, three-dimensional

Results are presented as n (%) or median (min-max) except for age (mean ± SD)

**Table S4** Clinical and ultrasound characteristics of 2403 adnexal tumors, according to whether tumor was difficult to classify as benign or malignant

|  | Both US examiner and LR1 not uncertain | | Either US examiner or LR1 uncertain | | P-value |
| --- | --- | --- | --- | --- | --- |
|  | N=2027 | | N=376 | |  |
| **Clinical variables** |  | |  | |  |
| Age, years | 49 ± 16 | | 53 ± 16 | | <0.001 |
| Postmenopausal | 861 | (42%) | 188 | (50%) | 0.007 |
| Hysterectomy | 115 | (6%) | 27 | (7%) | 0.27 |
| Hormonal replacement therapy | 174 | (9%) | 33 | (9%) | 0.90 |
| Personal history ovarian cancer | 36 | (2%) | 8 | (2%) | 0.67 |
| Family history ovarian cancer | 69 | (3%) | 5 | (1%) | 0.03 |
| CA125, N available | 1198 | | 253 | |  |
| CA125 | 52 (1 – 14067) | | 23 (3 – 1948) | | <0.001 |
| **Gray scale ultrasound variables** |  | |  | |  |
| Largest diameter, mm | 70 (10 – 550) | | 75 (10 – 322) | | 0.006 |
| Bilateral | 465 | (23%) | 53 | (14%) | <0.001 |
| Ascites | 330 | (16%) | 10 | (3%) | <0.001 |
| **Type of mass** |  |  |  |  | <0.001 |
| Unilocular | 585 | (29%) | 15 | (4%) | <0.001 |
| Unilocular solid | 187 | (9%) | 71 | (19%) | <0.001 |
| Multilocular | 331 | (16%) | 82 | (22%) | 0.05 |
| Multilocular solid | 375 | (19%) | 130 | (35%) | <0.001 |
| Solid | 549 | (27%) | 78 | (21%) | 0.04 |
| Nr locules | 1 (0 to >10) | | 2 (0 to >10) | | <0.001 |
| Pain at US examination | 294 | (15%) | 50 | (13%) | 0.54 |
| **Echogenicity of cyst fluid** |  |  |  |  |  |
| Anechoic | 485 | (24%) | 104 | (28%) | 0.52 |
| Low level | 392 | (19%) | 124 | (33%) | <0.001 |
| Ground glass | 326 | (16%) | 24 | (6%) | <0.001 |
| Hemorrhagic | 16 | (<1%) | 4 | (1%) | 0.98 |
| Mixed | 259 | (13%) | 42 | (11%) | 0.94 |
| No cyst fluid | 549 | (27%) | 78 | (21%) | 0.05 |
| Papillary projections present | 265 | (13%) | 118 | (31%) | <0.001 |
| Flow in papillation | 169 | (64%) | 46 | (39%) | <0.001 |
| Number of papillations | 2 (1 – ≥ 4) | | 2 (1 – ≥ 4) | | 0.006 |
| Height of papillation, mm | 11 (3 – 99) | | 7 (3 – 45) | | <0.001 |
| Mass with solid components | 1111 | (55%) | 279 | (74%) | <0.001 |
| Largest diameter of largest solid component, mm | 54 (3-300) | | 25 (3-200) | | <0.001 |
| Incomplete septum | 88 | (4%) | 25 | (7%) | 0.06 |
| Irregular walls | 740 | (37%) | 217 | (58%) | <0.001 |
| Shadows | 239 | (12%) | 60 | (16%) | 0.03 |
| **Doppler ultrasound variables** |  |  |  |  |  |
| Color Score |  |  |  |  | <0.001 |
| Score 1 | 553 | (27%) | 53 | (14%) | <0.001 |
| Score 2 | 604 | (30%) | 158 | (42%) | <0.001 |
| Score 3 | 539 | (27%) | 142 | (38%) | <0.001 |
| Score 4 | 331 | (16%) | 23 | (6%) | <0.001 |

US, ultrasound; LR, logistic regression model 1

Results are presented as n (%) or median (min-max) except for age (mean ± SD)

The P-values presented are corrected for multiple testing using the permutation method (Westfall PH, Wolfinger RD. Multiple tests with discrete distributions. *Am Stat* 1997; **51**: 3-8)
