# Supplementary material for: Vessel morphology depicted by three‐dimensional power Doppler ultrasound as second‐stage test in adnexal tumors that are difficult to classify: prospective diagnostic accuracy study
Source: Ultrasound Obstet Gynecol. 2021 Feb 1;57(2):324–34. doi: 10.1002/uog.22191 (PMC7898332; doi:10.1002/uog.22191)
Supplement: Supplementary file 6 — Table S5 Number of patients and proportion of difficult adnexal tumors contributed by each center [file UOG-57-324-s006.docx]

**Table S5** Number of patients and proportion of difficult adnexal tumors contributed by each center

|  | **ALL** | | | | | | | | | | | | **3D volume analyzed** | | | | | | | |
| --- | --- | --- | --- | --- | --- | --- | --- | --- | --- | --- | --- | --- | --- | --- | --- | --- | --- | --- | --- | --- |
| Center | **Total**  n (%) | | Both US examiner and LR1 not uncertain | | Either US examiner or LR1 uncertain | | US examiner uncertain | | LR1 uncertain | | Both uncertain | | Either US examiner or LR1 uncertain | | US examiner uncertain | | LR1 uncertain | | Both uncertain | |
| BIT | 213 | (9%) | 180 | (9%) | 33 | (9%) | 15 | (9%) | 21 | (8%) | 3 | (6%) | - | - | - | - | - | - | - | - |
| BSP | 37 | (2%) | 31 | (2%) | 6 | (2%) | 2 | (1%) | 4 | (2%) | - | - | 5 | (4%) | 2 | (3%) | 3 | (3%) | - | - |
| CIT | 218 | (9%) | 196 | (10%) | 22 | (6%) | 4 | (2%) | 21 | (8%) | 3 | (6%) | 11 | (8%) | 3 | (4%) | 10 | (11%) | 2 | (7%) |
| FIT | 21 | (<1%) | 20 | (<1%) | 1 | (<1%) | 1 | (<1%) | - | - | - | - | - | - | - | - | - | - | - | - |
| GBE | 228 | (9%) | 192 | (9%) | 36 | (10%) | 19 | (11%) | 24 | (9%) | 7 | (14%) | 4 | (3%) | 2 | (3%) | 3 | (3%) | 1 | (4%) |
| GIT | 6 | (<1%) | 5 | (<1%) | 1 | (<1%) | 1 | (<1%) | - | - | - | - | - | - | - | - | - | - | - | - |
| LBE | 129 | (5%) | 98 | (5%) | 31 | (8%) | 17 | (10%) | 16 | (6%) | 2 | (4%) | 2 | (1%) | 1 | (1%) | 1 | (1%) | - | - |
| LPO | 131 | (5%) | 117 | (6%) | 14 | (4%) | 4 | (2%) | 10 | (4%) | - | - | 2 | (1%) | 1 | (1%) | 1 | (1%) | - | - |
| LSW | 39 | (2%) | 30 | (1%) | 9 | (2%) | 6 | (4%) | 6 | (2%) | 3 | (6%) | 4 | (3%) | 3 | (4%) | 3 | (3%) | 2 | (7%) |
| MIT | 86 | (4%) | 75 | (4%) | 11 | (3%) | - | - | 11 | (4%) | - | - | - | - | - | - | - | - | - | - |
| MSW | 201 | (8%) | 141 | (7%) | 60 | (16%) | 32 | (19%) | 39 | (15%) | 11 | (22%) | 50 | (36%) | 31 | (39%) | 30 | (34%) | 11 | (39%) |
| NIT | 8 | (<1%) | 7 | (<1%) | 1 | (<1%) | - | - | 1 | (<1%) | - | - | - | - | - | - | - | - | - | - |
| OIT | 105 | (4%) | 91 | (4%) | 14 | (4%) | 7 | (4%) | 10 | (4%) | 3 | (6%) | 12 | (9%) | 7 | (9%) | 8 | (9%) | 3 | (11%) |
| PCR | 264 | (11%) | 234 | (12%) | 30 | (8%) | 3 | (2%) | 29 | (11%) | 2 | (4%) | 12 | (9%) | 3 | (4%) | 11 | (13%) | 2 | (7%) |
| RIT | 443 | (18%) | 386 | (19%) | 57 | (15%) | 26 | (15%) | 37 | (14%) | 6 | (12%) | 10 | (7%) | 9 | (11%) | 4 | (5%) | 3 | (11%) |
| SIT | 107 | (4%) | 100 | (5%) | 7 | (2%) | 1 | (<1%) | 6 | (2%) | - | - | 6 | (4%) | 1 | (1%) | 5 | (6%) | - | - |
| SSW | 120 | (5%) | 85 | (4%) | 35 | (9%) | 27 | (16%) | 16 | (6%) | 8 | (16%) | 20 | (14%) | 16 | (20%) | 8 | (9%) | 4 | (14%) |
| UDI | 47 | (2%) | 39 | (2%) | 8 | (2%) | 3 | (2%) | 8 | (3%) | 3 | (6%) | - | - | - | - | - | - | - | - |
| **All centers** | **2403** |  | **2027** |  | **376** |  | **168** |  | **259** |  | **51** |  | **138** |  | **79** |  | **87** |  | **28** |  |

Percentages are calculated per column

US, ultrasound; LR1, logistic regression model 1; 3D, three-dimensional

BIT, Bologna, Italy; BSP, Barcelona, Spain; CIT, European Institute of Oncology, Milan,  Italy; FIT,  Children´s Hospital Buzzi, Milan Italy; GBE, Genk, Belgium; GIT, Instituto Nationale dei Tumori, Naples, Italy; LBE, Leuven, Belgium; LPO, Lublin, Poland; LSW, Lund, Sweden; MIT, Sacco University, Milan, Italy; MSW, Malmoe, Sweden; NIT, Universita degli Studi di Napoli, Naples, Italy; OIT, Monza, Italy; PCR, Prague, Czeck Republic; RIT, Rome, Italy; SIT, Cagliari, Italy; SSW, Stockholm, Sweden; UDI, Udine, Italy
