# Supplementary material for: Vessel morphology depicted by three‐dimensional power Doppler ultrasound as second‐stage test in adnexal tumors that are difficult to classify: prospective diagnostic accuracy study
Source: Ultrasound Obstet Gynecol. 2021 Feb 1;57(2):324–34. doi: 10.1002/uog.22191 (PMC7898332; doi:10.1002/uog.22191)
Supplement: Supplementary file 7 — Tables S6 and S7 Histological diagnoses (Table S6) and clinical and ultrasound characteristics (Table S7) of 376 difficult adnexal tumors, according to whether ultrasound volumes were available [file UOG-57-324-s007.docx]

**Table S6** Histological diagnoses of 376 difficult adnexal tumors, according to whether ultrasound volumes were available

|  | Either ultrasound examiner or LR1 uncertain  N=376 | | | | |
| --- | --- | --- | --- | --- | --- |
|  | Volume not available  N=238 | | Volume available and analyzed  N=138 | | P-value |
| **Benign** | **154** | **(65%)** | **100** | **(72%)** | **0.32** |
| Endometrioma | 13 | (5%) | 7 | (5%) |  |
| Teratoma | 14 | (6%) | 5 | (4%) |  |
| Simple cyst or parasalpingeal cyst | 7 | (3%) | 3 | (2%) |  |
| Functional cyst | 8 | (3%) | 3 | (2%) |  |
| Hydrosalpinx or salpingitis | 4 | (2%) | 3 | (2%) |  |
| Peritoneal pseudocyst | 2 | (<1%) | 2 | (1%) |  |
| Abscess | 1 | (<1%) | 2 | (1%) |  |
| Fibroma | 33 | (14%) | 18 | (13%) |  |
| Serous cystadenoma | 39 | (16%) | 30 | (22%) |  |
| Mucinous cystadenoma | 26 | (11%) | 23 | (17%) |  |
| Rare benign | 7 | (3%) | 4 | (3%) |  |
|  |  |  |  |  |  |
| **Borderline** | **35** | **(15%)** | **15** | **(11%)** | **0.69** |
| Stage I | 35 | (15%) | 14 | (10%) |  |
| Stage II | 0 | 0 | 1 | (<1%) |  |
| Stage III or IV | 0 | 0 | 0 | 0 |  |
|  |  |  |  |  |  |
| **Primary invasive** | **42** | **(18%)** | **22** | **(16%)** | **0.98** |
| Stage I | 18 | (8%) | 7 | (5%) |  |
| Stage II | 2 | (<1%) | 1 | (<1%) |  |
| Stage III | 11 | (5%) | 8 | (6%) |  |
| Stage IV | 1 | (<1%) | 0 | 0 |  |
| Rare | 10 | (4%) | 6 | (4%) |  |
|  |  |  |  |  |  |
| **Metastatic** | **7** | **(3%)** | **1** | **(<1%)** | **0.60** |

The P-values presented have been corrected for multiple testing using the permutation
 method (Westfall PH, Wolfinger RD. Multiple tests with discrete distributions. *Am Stat* 1997; **51**: 3-8)

**Table S7** Clinical and ultrasound characteristics of 376 difficult adnexal tumors, according to whether ultrasound volumes were available

|  | Either ultrasound examiner or LR1 uncertain | | | | |
| --- | --- | --- | --- | --- | --- |
|  | Volume not available  N=238 | | Volume available and analyzed  N=138 | | P-value |
| **Clinical variables** |  | |  | |  |
| Age, years | 52 ± 16 | | 54 ± 17 | | 0.50 |
| Postmenopausal | 115 | (48%) | 73 | (53%) | 0.39 |
| Hysterectomy | 18 | (8%) | 9 | (7%) | 0.84 |
| Hormonal replacement therapy | 20 | (8%) | 13 | (9%) | 0.74 |
| Personal history ovarian cancer | 4 | (2%) | 4 | (3%) | 0.47 |
| Family history ovarian cancer | 2 | (<1%) | 3 | (2%) | 0.36 |
| CA125, number available | 136 | | 117 | |  |
| CA125, U/mL | 29 (3 - 1948) | | 20 (4 - 1302) | | 0.61 |
| **Gray scale ultrasound variables** |  | |  | |  |
| Largest diameter, mm | 80 (14-322) | | 69 (10-310) | | 0.06 |
| Bilateral | 31 | (13%) | 22 | (16%) | 0.44 |
| Ascites | 5 | (2%) | 5 | (4%) | 0.51 |
| Type of mass |  |  |  |  | 0.06 |
| Unilocular | 14 | (6%) | 1 | (<1%) |  |
| Unilocular solid | 48 | (20%) | 23 | (17%) |  |
| Multilocular | 50 | (21%) | 32 | (23%) |  |
| Multilocular solid | 74 | (31%) | 56 | (41%) |  |
| Solid | 52 | (22%) | 26 | (19%) |  |
| Number of locules if multilocular or multilocular solid |  |  |  |  | 0.17 |
| 2 | 19 | (15%) | 10 | (11%) |  |
| 3 | 16 | (13%) | 8 | (9%) |  |
| 4 | 9 | (7%) | 8 | (9%) |  |
| 5-10 | 37 | (30%) | 23 | (26%) |  |
| >10 | 43 | (35%) | 39 | (44%) |  |
| Tender mass at ultrasound examination | 41 | (17%) | 9 | (7%) | 0.003 |
| Echogenicity of cyst fluid |  |  |  |  | 0.65 |
| Anechoic | 68 | (29%) | 36 | (26%) |  |
| Low level | 77 | (32%) | 47 | (34%) |  |
| Ground glass | 14 | (6%) | 10 | (7%) |  |
| Hemorrhagic | 1 | (<1%) | 3 | (2%) |  |
| Mixed | 26 | (11%) | 16 | (12%) |  |
| No cyst fluid | 52 | (22%) | 26 | (19%) |  |
| Papillary projections present | 73 | (31%) | 45 | (33%) | 0.70 |
| Flow in papillation, if papillation present | 26 | (36%) | 20 | (44%) | 0.34 |
| Number of papillations | 2 (1 – ≥4) | | 2 (1 – ≥4) | | 0.92 |
| Height of papillation, mm | 7 (3 – 30) | | 7 (3 – 45) | | 0.27 |
| Mass with solid components | 174 | (73%) | 105 | (76%) | 0.52 |
| Largest diameter of largest solid component, mm | 26 (4-200) | | 24 (3-180) | | 0.45 |
| Incomplete septum | 23 | (10%) | 2 | (1%) | 0.002 |
| Irregular walls | 140 | (59%) | 77 | (56%) | 0.57 |
| Shadows | 40 | (17%) | 20 | (14%) | 0.55 |
| **Doppler ultrasound variables** |  |  |  |  |  |
| Color Score |  |  |  |  | <0.001 |
| Score 1 | 43 | (18%) | 10 | (7%) |  |
| Score 2 | 114 | (48%) | 44 | (32%) |  |
| Score 3 | 72 | (30%) | 70 | (51%) |  |
| Score 4 | 9 | (4%) | 14 | (10%) |  |

LR1, logistic regression model 1

Results are shown as n (%) or median (min – max) except for age (mean ± SD)

No correction for multiple testing because no further testing was done for the subcategories
