# Supplementary material for: Vessel morphology depicted by three‐dimensional power Doppler ultrasound as second‐stage test in adnexal tumors that are difficult to classify: prospective diagnostic accuracy study
Source: Ultrasound Obstet Gynecol. 2021 Feb 1;57(2):324–34. doi: 10.1002/uog.22191 (PMC7898332; doi:10.1002/uog.22191)
Supplement: Supplementary file 8 — Table S8 Ability (with 95% CI) of vessel morphology on 3D power Doppler, subjective ultrasound assessment and IOTA logistic regression model 1 to discriminate correctly between benign and malignant difficult adnexal tumors [file UOG-57-324-s008.docx]

**Table S8** Ability (with 95% CI) of vessel morphology on 3D power Doppler, subjective ultrasound assessment and IOTA logistic regression model 1 to discriminate correctly between benign and malignant difficult adnexal tumors

| **Diagnostic method** | **Sensitivity [95% CI]** | | | **Specificity [95% CI]** | | | **LR+ [95% CI]** | | **LR- [95% CI]** | | **P-value** |
| --- | --- | --- | --- | --- | --- | --- | --- | --- | --- | --- | --- |
| **Either US examiner or LR1 uncertain (N=138)** |  |  |  |  |  |  |  |  |  |  |  |
| Whole tumor vessel morphology |  |  |  |  |  |  |  |  |  |  |  |
| Branching vessels | 89% | (34/38) | [76% to 96%] | 33% | (33/100) | [25% to 43%] | 1.34 | [1.12 to 1.59] | 0.32 | [0.12 to 0.84] | 0.009 |
| Densely packed vessels | 63% | (24/38) | [47% to 77%] | 83% | (83/100) | [74% to 89%] | 3.72 | [2.26 to 6.10] | 0.44 | [0.29 to 0.68] | <0.001 |
| Caliber changes in vessels | 66% | (25/38) | [50% to 79%] | 68% | (68/100) | [58% to 76%] | 2.06 | [1.43 to 2.97] | 0.50 | [0.32 to 0.80] | <0.001 |
| Splashes | 50% | (19/38) | [35% to 65%] | 78% | (78/100) | [69% to 85%] | 2.27 | [1.40 to 3.70] | 0.64 | [0.46 to 0.90] | 0.002 |
| Tortuous vessels | 66% | (25/38) | [50% to 79%] | 70% | (70/100) | [60% to 78%] | 2.19 | [1.50 to 3.20] | 0.49 | [0.31 to 0.77] | <0.001 |
|  |  |  |  |  |  |  |  |  |  |  |  |
| Biopsy vessel morphology |  |  |  |  |  |  |  |  |  |  |  |
| Branching vessels | 84% | (32/38) | [70% to 93%] | 34% | (34/100) | [25% to 44%] | 1.28 | [1.05 to 1.55] | 0.46 | [0.21 to 1.02] | 0.04 |
| Caliber changes in vessels | 79% | (30/38) | [64% to 89%] | 63% | (63/100) | [53% to 72%] | 2.13 | [1.58 to 2.89] | 0.33 | [0.18 to 0.63] | <0.001 |
| Splashes | 53% | (20/38) | [37% to 68%] | 69% | (69/100) | [59% to 77%] | 1.70 | [1.12 to 2.58] | 0.69 | [0.48 to 0.98] | 0.02 |
| Tortuous vessels | 79% | (30/38) | [64% to 89%] | 64% | (64/100) | [54% to 73%] | 2.19 | [1.61 to 2.99] | 0.33 | [0.18 to 0.62] | <0.001 |
| Bridges between vessels | 42% | (16/38) | [28% to 58%] | 81% | (81/100) | [72% to 87%] | 2.22 | [1.28 to 3.84] | 0.72 | [0.54 to 0.95] | 0.007 |
| Subjective assessment | 74% | (28/38) | [58% to 85%] | 74% | (74/100) | [65% to 82%] | 2.83 | [1.94 to 4.15] | 0.36 | [0.21 to 0.61] | <0.001 |
| LR1 (10% risk cutoff) | 92% | (35/38) | [79% to 97%] | 23% | (23/100) | [16% to 32%] | 1.20 | [1.04 to 1.38] | 0.34 | [0.11 to 1.08] | 0.03 |
|  |  |  |  |  |  |  |  |  |  |  |  |
| **US examiner uncertain (N=79)** |  |  |  |  |  |  |  |  |  |  |  |
| Whole tumor vessel morphology |  |  |  |  |  |  |  |  |  |  |  |
| Branching vessels | 89% | (24/27) | [72% to 96%] | 33% | (17/52) | [22% to 46%] | 1.32 | [1.05 to 1.67] | 0.34 | [0.11 to 1.06] | 0.06 |
| Densely packed vessels | 67% | (18/27) | [48% to 81%] | 83% | (43/52) | [70% to 91%] | 3.85 | [2.01 to 7.39] | 0.40 | [0.23 to 0.70] | <0.001 |
| Caliber changes in vessels | 63% | (17/27) | [44% to 78%] | 67% | (35/52) | [54% to 78%] | 1.93 | [1.19 to 3.13] | 0.55 | [0.33 to 0.93] | 0.01 |
| Splashes | 52% | (14/27) | [34% to 69%] | 83% | (43/52) | [70% to 91%] | 3.00 | [1.49 to 6.01] | 0.58 | [0.39 to 0.88] | 0.003 |
| Tortuous vessels | 63% | (17/27) | [44% to 78%] | 63% | (33/52) | [50% to 75%] | 1.72 | [1.09 to 2.73] | 0.58 | [0.34 to 0.99] | 0.02 |
|  |  |  |  |  |  |  |  |  |  |  |  |
| Biopsy vessel morphology |  |  |  |  |  |  |  |  |  |  |  |
| Branching vessels | 85% | (23/27) | [68% to 94%] | 35% | (18/52) | [23% to 48%] | 1.30 | [1.01 to 1.68] | 0.43 | [0.16 to 1.14] | 0.07 |
| Caliber changes in vessels | 78% | (21/27) | [59% to 89%] | 62% | (32/52) | [48% to 74%] | 2.02 | [1.36 to 3.01] | 0.36 | [0.17 to 0.76] | 0.001 |
| Splashes | 59% | (16/27) | [41% to 75%] | 71% | (37/52) | [58% to 82%] | 2.05 | [1.21 to 3.49] | 0.57 | [0.35 to 0.93] | 0.009 |
| Tortuous vessels | 81% | (22/27) | [63% to 92%] | 67% | (35/52) | [54% to 78%] | 2.49 | [1.62 to 3.83] | 0.28 | [0.12 to 0.62] | <0.001 |
| Bridges between vessels | 44% | (12/27) | [28% to 63%] | 79% | (41/52) | [66% to 88%] | 2.10 | [1.07 to 4.11] | 0.71 | [0.49 to 1.02] | 0.03 |
| Subjective assessment | 74% | (20/27) | [55% to 87%] | 60% | (31/52) | [46% to 72%] | 1.83 | [1.23 to 2.73] | 0.44 | [0.22 to 0.86] | 0.004 |
| LR1 (10% risk cutoff) | 89% | (24/27) | [72% to 96%] | 19% | (10/52) | [11% to 32%] | 1.10 | [0.91 to 1.33] | 0.58 | [0.17 to 1.93] | 0.34 |
|  |  |  |  |  |  |  |  |  |  |  |  |
| **LR1 uncertain (N=87)** |  |  |  |  |  |  |  |  |  |  |  |
| Whole tumor vessel morphology |  |  |  |  |  |  |  |  |  |  |  |
| Branching vessels | 94% | (16/17) | [73% to 99%] | 30% | (21/70) | [21% to 42%] | 1.35 | [1.11 to 1.63] | 0.20 | [0.03 to 1.36] | 0.06 |
| Densely packed vessels | 53% | (9/17) | [31% to 74%] | 83% | (58/70) | [72% to 90%] | 3.09 | [1.56 to 6.11] | 0.57 | [0.34 to 0.95] | 0.004 |
| Caliber changes in vessels | 71% | (12/17) | [47% to 87%] | 69% | (48/70) | [57% to 78%] | 2.25 | [1.41 to 3.57] | 0.43 | [0.20 to 0.91] | 0.005 |
| Splashes | 47% | (8/17) | [26% to 69%] | 77% | (54/70) | [66% to 85%] | 2.06 | [1.06 to 4.00] | 0.69 | [0.43 to 1.09] | 0.07 |
| Tortuous vessels | 65% | (11/17) | [41% to 83%] | 71% | (50/70) | [60% to 81%] | 2.27 | [1.36 to 3.77] | 0.49 | [0.26 to 0.96] | 0.01 |
| Biopsy vessel morphology |  |  |  |  |  |  |  |  |  |  |  |
| Branching vessels | 76% | (13/17) | [53% to 90%] | 31% | (22/70) | [22% to 43%] | 1.12 | [0.82 to 1.52] | 0.75 | [0.30 to 1.89] | 0.77 |
| Caliber changes in vessels | 76% | (13/17) | [53% to 90%] | 64% | (45/70) | [53% to 75%] | 2.14 | [1.42 to 3.23] | 0.37 | [0.15 to 0.88] | 0.005 |
| Splashes | 47% | (8/17) | [26% to 69%] | 69% | (48/70) | [57% to 78%] | 1.50 | [0.81 to 2.76] | 0.77 | [0.48 to 1.24] | 0.26 |
| Tortuous vessels | 71% | (12/17) | [47% to 87%] | 60% | (42/70) | [48% to 71%] | 1.77 | [1.16 to 2.69] | 0.49 | [0.23 to 1.05] | 0.03 |
| Bridges between vessels | 35% | (6/17) | [17% to 59%] | 81% | (57/70) | [71% to 89%] | 1.90 | [0.85 to 4.27] | 0.80 | [0.55 to 1.15] | 0.19 |
| Subjective assessment | 82% | (14/17) | [59% to 94%] | 79% | (55/70) | [68% to 87%] | 3.84 | [2.33 to 6.33] | 0.23 | [0.08 to 0.63] | <0.001 |
| LR1 (10% risk cutoff) | 100% | (17/17) | [82% to 100%] | 19% | (13/70) | [11% to 29%] | 1.23 | [1.10 to 1.37] | Not possible to calculate | | 0.06 |
|  |  |  |  |  |  |  |  |  |  |  |  |

LR+, positive likelihood ratio; LR-, negative likelihood ratio; CI, confidence interval; LR1, logistic regression model 1 using the 10% risk cutoff to predict malignancy suggested in [*J Clin Oncol*](https://www.ncbi.nlm.nih.gov/pubmed/?term=journal+of+clinical+oncology+AND+2005+AND+valentin+AND+timmerman) 2005; **23:** 8794-8801 and *Ultrasound Obstet Gynecol* 2010; **36:** 226-234

No corrections have been made for multiple testing because this is an exploratory study
